# Supplementary material for: Vulnerability in research ethics: A systematic review of policy guidelines and documents
Source: PLoS One. 2025 Jul 1;20(7):e0327086. doi: 10.1371/journal.pone.0327086 (PMC12212517; doi:10.1371/journal.pone.0327086)
Supplement: S1 Table — (DOCX) [file pone.0327086.s001.docx]

The following tables collect the excluded documents through the screening process. The documents came from:

- three overview lists: *International Compilation of Human Research Standards* (2024 edition), *Listing of Social-behavioral Research Standards* (2018 edition) from the US Department of Health and Human Services, and *Ethics Legislation, Regulation and Conventions* from the European Commission’s Horizon 2020 programme;
- two major databases: PubMed and Web of Science;
- grey literature search: Google Scholar.

The documents' order depends on the sequence in which they were found.

**S1 Table: List of documents excluded from title screening and text skimming**

| **TITLE** | **REASON FOR EXCLUSION** |
| --- | --- |
| **INTERNATIONAL COMPILATION OF HUMAN RESEARCH STANDARDS (2024 edition)** | |
| PACTR, FAQs. Pan African Clinical Trials Registry. Clinical Trial Registries | document type |
| PACTR, Terms and Conditions. Pan African Clinical Trials Registry. Clinical Trial Registries | document type |
| Provision ANMAT 6677/10: Regulatory Guideline for Good Clinical Practices in Clinical Pharmacological Studies (2010). National Administration of Drugs, Foods, and Medical Devices (ANMAT). Drugs, Biologics, and Devices | language |
| Provision ANMAT 4009/2017: Health Care Institutions: Requirements and Conditions of  Authorization for Conducting Phase I and/or Bioequivalence Clinical Pharmacology Studies. National Administration of Drugs, Foods, and Medical Devices (ANMAT). Drugs, Biologics, and Devices | language |
| Australian States and Territories, National Mutual Acceptance of Scientific and Ethical Review of Multi-Centre Human Research. National Health and Medical Research Council (NHMRC), Australian Research Council (ARC), Australian Institute of Aboriginal and Torres Strait Islander Studies (AIATSIS). General | domain |
| FAQs. National Health and Medical Research Council and the Department of Industry, Innovation, and Science e Australian New Zealand Clinical Trials Registry. Clinical Trials Registry | document type |
| Gene Technology Act 2000 (2016). National Health and Medical Research Council (NHMRC) e Office of the Gene Technology Regulator. Genetic Research | domain |
| Gene Technology Regulations 2001 (2016). National Health and Medical Research Council (NHMRC) e Office of the Gene Technology Regulator. Genetic Research | domain |
| Guidelines under Section 95 of the Privacy Act 1988 (2014). Office of the Australian Information Commissioner. Privacy/Data Protection | duplicate |
| Medicines Australia, Industry Standard Compensation Guidelines (2012). Therapeutic Goods Administration (TGA), Medicines Australia e National Health and Medical Research Council (NHMRC). Research Injury | domain |
| TGA, Australian Regulatory Guidelines for Biologicals (2017). Therapeutic Goods Administration (TGA). Human Biological Materials | domain |
| Effects of resistance training and nutritional support on osteosarcopenia in older, community-dwelling postmenopausal Korean females (ERTO-K study) | duplicate |
| Therapeutic Goods (Medical Devices) Regulations 2002 (2016). Therapeutic Goods Administration (TGA). Drugs, Biologics, and Devices | domain |
| Therapeutic Goods Act 1989 (2016). Therapeutic Goods Administration (TGA). Drugs, Biologics, and Devices | domain |
| Bioethics Commission, various publications. Ministry of Health, Forum of Austrian Ethics Committees e Bioethics Commission. General | document type |
| University Act (2011). Ministry of Health, Forum of Austrian Ethics Committees e Bioethics Commission. General | domain |
| Drugs (Control) Ordinance 1982, Ordinance No. VIII. Bangladesh Directorate of Drug Administration. Drugs, Biologics, and Devices | domain |
| Guidelines for Transfer of Human Biological Materials Abroad for Research Purposes (2004). Bangladesh Medical Research Council, National Research Ethics Committee. Human Biological Materials | domain |
| Standard Operating Procedures (SOPs). Bangladesh Medical Research Council, National Research Ethics Committee. General | domain |
| The Drugs Act (1940). Bangladesh Directorate of Drug Administration. Drugs, Biologics, and Devices | domain |
| Constitution of the Republic of Belarus, Article 25 (2004). Ministry of Health (MOH), National Bioethics Committee eCenter for examinations and tests in health service. General | document type |
| BACB, Opinion No. 58: Financing Expensive Medication. Belgian Advisory Committee on Bioethics (BACB). Drugs, Biologics, and Devices | domain |
| BACB, various. Belgian Advisory Committee on Bioethics (BACB). General | document type |
| CSS, various. Superior Health Council (CSS). Human Biological Materials | document type |
| FAMHP, Various Circulars:. Federal Agency for Medicines and Health Products (FAMHP). General | document type |
| New Political Constitution of the State, Article 44 (2009). Ministry of Health and Sport (MHS) e National Bioethics Committee (NBC). General | domain |
| Anthropological Research Act 45 (1967). Ministry of Health and Wellness. General | domain |
| Drugs and Related Substances Regulations (1993). Ministry of Health and Wellness. Drugs, Biologics, and Devices | domain |
| Guide for a Consent Form (2005). Ministry of Health and Wellness. General | document type |
| WHO Expert Group on Ethics and Governance of Artificial Intelligence for Health | document type |
| WHO Human Genome Editing Recommendations e Registry | Excluded because it's a document regarding Embryos, Stem Cells and Cloning |
| FAQs. Brazilian Clinical Trials Registry. Clinical Trial Registries | document type |
| Resolution CNS No. 251/1997: On Complimentary Rules for Research with New Pharmaceutical Products, Medicines, Vaccines, and Diagnostic Tests. National Health Council (CNS). Drugs, Biologics, and Devices | domain |
| Resolution CNS No. 340/2004: On Research on Human Genetics (2004). National Commission on Research Ethics (CONEP), National Biosafety Technical Commission (CTNBio) e National Health Council (CNS). Genetic Research | Excluded because it's a document regarding Embryos, Stem Cells and Cloning |
| Resolution CNS No. 346/2005 on Multicenter Research. National Health Council (CNS) e National Commission on Research Ethics (CONEP). General | domain |
| Global Repository on National Digital Health Strategies (WHO) | document type |
| European Medicines Agency - Clinical Trials Information System (CTIS) | document type |
| Standards Survey of New Drugs, Medicines, Vaccines, and Diagnostic Tests Involving Human Beings - Resolution CNS No. 251/97. Brazilian Health Surveillance Agency, National Health Council (CNS) e National Commission on Research Ethics (CONEP). Research Injury | domain |
| Act on Transplantation of Organs, Tissues and Cells. Ministry of Healthcare. Human Biological Materials | domain |
| Medical Devices Act. Ministry of Healthcare (MOH) e Bulgarian Drug Agency (BDA). Drugs, Biologics, and Devices | domain |
| Regulation No. 13 of 4 April 2007 for the Terms and Conditions of Informing Bulgarian Citizens on the Activities regarding the Transplantation of Organs, Tissues and Cells. Executive Agency Medical Supervision. Human Biological Materials | domain |
| Research (2018). Bulgarian Commission for Personal Data Protection e Ombudsman. Privacy/Data Protection | language |
| Various. Bulgarian Drug Agency (BDA). Drugs, Biologics, and Devices | document type |
| Ministerial Order No. 079/A/MSP/DS of MINSANTE (1987). Cameroon Bioethics Initiative. General | language |
| Practical Assessment and Management of Vulnerabilities in Older Patients Receiving Systemic Cancer Therapy: ASCO Guideline Update | duplicate |
| African Clinical Trials Community | document type |
| OPC: SOR/2001-6, SOR/2001-7, and SOR/2001-8 (September 29, 2014). Office of the Privacy Commissioner of Canada (OPC), Interagency Advisory Panel on Research Ethics (PRE) e Canadian Institutes of Health Research (CIHR). Privacy/Data Protection | document type |
| Personal Information Protection and Electronic Documents Act, Articles 5 and 7 (2001). Office of the Privacy Commissioner of Canada (OPC), Interagency Advisory Panel on Research Ethics (PRE) e Canadian Institutes of Health Research (CIHR). Privacy/Data Protection | domain |
| Regulations Amending the Food and Drug Regulations (1024 – Clinical Trials) (2001). Health Canada, Therapeutic Products Directorate e Interagency Advisory Panel on Research Ethics (PRE). Drugs, Biologics, and Devices | domain |
| Law for Good Clinical Practice (2004). Ministry of Health, Pharmaceutical Services e Ministry of Health, National Bioethics Committee. Drugs, Biologics, and Devices | language |
| Act. No. 373/2011 on Specific Healthcare Services, As Amended (2018). Ministry of Health, Central Ethics Committee. General | domain |
| Legislation Concerning Medicinal Products of Human Use (Good Clinical Practice) No. 452/2004 Article 11 (8). Ministry of Health, Pharmaceutical Services. Research Injury | language |
| Act No. 378/2007 Collection on Pharmaceuticals, As Amended (2019). Ministry of Health (MOH), State Institute for Drug Control (SUKL). Drugs, Biologics, and Devices | domain |
| Act No. 89/2021 Coll., on Medical Devices. State Institute for Drug Control (SUKL). Drugs, Biologics, and Devices | domain |
| Act No. 90/2021 Coll, on Medical Devices (the “Act on In Vitro Diagnostic Medical Devices”). State Institute for Drug Control (SUKL). Drugs, Biologics, and Devices | duplicate |
| EU Clinical Trials Register. Clinical Trial Registries | document type |
| Law No. 89/2012 Coll. Civil Code. Research Injury | domain |
| Various. Ministry of Health (MOH), State Institute for Drug Control (SUKL). Drugs, Biologics, and Devices | document type |
| Various. State Institute for Drug Control (SUKL). Drugs, Biologics, and Devices | document type |
| Regulation No. 536 on Clinical Trials on Medicinal Products for Human Use (2014). Committees on Medicine Research Ethics (VMK) e Danish Medicines Agency. Drugs, Biologics, and Devices | duplicate |
| Regulation No. 745 on Medical Devices (2017). Committees on Medicine Research Ethics (VMK) e Danish Medicines Agency. Drugs, Biologics, and Devices | domain |
| Constitution of the Arab Republic of Egypt, Article 43. Medical Professionals Union. General | document type |
| Code of Ethics of Estonian Scientists. Estonian Council on Bioethics. General | document type |
| Constitution of the Republic of Estonia, Paragraph 18 (2016). Estonian Council on Bioethics. General | duplicate |
| Human Genes Research Act (RT I 2000, 104, 685) (2014). Genetic Research | domain |
| International Data Transfer (2018). Estonian Data Protection Inspectorat. Privacy/Data Protection | duplicate |
| Medical Devices Act (2004). State Agency of Medicines, Minister of Social Affairs (MSA) e Estonian Health Board. Drugs, Biologics, and Devices | domain |
| Medicinal Products Act, Chapter 5 (2015). State Agency of Medicines, Minister of Social Affairs (MSA) e Estonian Health Board. Drugs, Biologics, and Devices | domain |
| Medicinal Products Act, Section 90. Minister of Social Affairs (MSA) e Estonian Health Insurance Fund. Research Injury | domain |
| Drug Administration and Control Proclamation No. 176/1999, Article 21 (1999). Food, Medicine, and Health Administration and Control Authority. Drugs, Biologics, and Devices | domain |
| Policies, various. Food, Medicine, and Health Administration and Control Authority. Drugs, Biologics, and Devices | document type |
| Proclamations, various. Food, Medicine, and Health Administration and Control Authority. Drugs, Biologics, and Devices | document type |
| Regulations, various. Food, Medicine, and Health Administration and Control Authority. Drugs, Biologics, and Devices | document type |
| Commission Implementing Regulation (EU) 2017/556 of 24 March 2017 on the Detailed  Arrangements for the Good Clinical Practice Inspection Procedures Pursuant to Regulation (EU) No. 536/2014 of the European Parliament and Council. European Commission, DG SANTE: Directorate-General for Health and Food Safety. Drugs, Biologics, and Devices | domain |
| Directive 2004/23/EC on Setting Standards of Quality and Safety for the Donation, Procurement, Testing, Processing, Preservation, Storage, and Distribution of Human Tissues and Cells. European Commission, European Group on Ethics in Science and New Technologies. Human Biological Materials | duplicate |
| Directive 98/79/EC on in vitro Diagnostic Medical Devices (IVD). European Medicines Agency. Drugs, Biologics, and Devices | domain |
| FAQs. EU Clinical Trials Register. Clinical Trial Registries | document type |
| Horizon 2020: How to Complete your Ethics Self –Assessment (2015). European Commission, European Group on Ethics in Science and New Technologies (EGE) e European Commission, Directorate-General for Research and Innovation. General | document type |
| Opinion 3/2019 concerning the Questions and Answers on the interplay between the Clinical Trials Regulation (CTR) and the General Data Protection regulation (GDPR) (2019). European Data Protection Board (EDPB). Privacy/Data Protection | document type |
| Questions and Answers on the European Medicines Agency Policy on Publication of Clinical Data for Medicinal Products for Human Use (2015). European Medicines Agency (EMA). Privacy/Data Protection | document type |
| Recommendation No. R (92) on Genetic Testing and Screening for Health Care Purposes (1992). Council of Europe, Bioethics Unit. Genetic Research | Excluded because it's a document regarding Embryos, Stem Cells and Cloning |
| Recommendation Rec (2006) 4 of the Committee of Ministers to Members States on Research on Biological Materials of Human Origin (2016). Council of Europe, Bioethics Unit. Genetic Research | domain |
| Recommendation Rec (2016) 6 of the Committee of Ministers to Member States on Research on Biological Materials of Human Origin. Council of Europe, Bioethics Unit. Human Biological Materials | domain |
| Various. European Medicines Agency. Drugs, Biologics, and Devices | document type |
| Agreeing on Authorship. Recommendation for Research Publications. Ministry of Social Affairs and Health, National Committee on Medical Research Ethics (TUKIJA), Finnish Advisory Board on Research Integrity (TENK), Finnish Institute for Health and Welfare (THL), Findata e Finnish Medicines Agency Fimea. General | domain |
| Criminal Code of Finland (39/1889, numerous amendments). Ministry of Social Affairs and Health, National Committee on Medical Research Ethics (TUKIJA), Finnish Advisory Board on Research Integrity (TENK), Finnish Institute for Health and Welfare (THL), Findata e Finnish Medicines Agency Fimea. General | domain |
| EU Regulations, In Vitro Diagnostic Medical Devices Regulation 2017/746. National Supervisory Authority for Welfare and Health (VALVIRA). Drugs, Biologics, and Devices | duplicate |
| Government Decree on the National Institute for Health and Welfare (668/2008), latest amendment 1122/2015. Ministry of Social Affairs and Health, National Committee on Medical Research Ethics (TUKIJA), Finnish Advisory Board on Research Integrity (TENK), Finnish Institute for Health and Welfare (THL), Findata e Finnish Medicines Agency Fimea. General | domain |
| Law on Medicines and Pharmaceutical Activities No. 659 and 1586 (2015). Bioethics and Health Law Studies Society. General | domain |
| Various. Bioethics and Health Law Studies Society. Human Biological Materials | document type |
| Various. Office of the Personal Data Protection Inspector. Privacy/Data Protection | document type |
| Ethical challenges in research regarding aging population | domain |
| Understanding sexual behaviors of youth from the lens of caregivers, teachers, local leaders and youth in Homabay County, Kenya | duplicate |
| Various. Social and Psychological Agency. Social-Behavioral Research | document type |
| BÄK, (Model) Professional Code for Physicians in Germany, Article 15 (2018). German Medical Association (BÄK). General | document type |
| German Ethics Council, Opinion on Human Biobanks for Research (2010). German Ethics Council. Human Biological Materials | duplicate |
| Emergency department crowding: An examination of older adults and vulnerability | domain |
| Clinical Guidelines for the Diagnosis and Treatment of Fragility Fractures of the Pelvis | domain |
| German Research Foundation, Statements and Publications. German Society of Human Genetics (GfH) e German Research Foundation (DFG), Permanent Senate Commission on Genetic Research. Genetic Research | domain |
| Medicinal Products Act, Section 40(3) (2020). Research Injury | duplicate |
| Clinical Trials, Biological Products, Devices, and More, Guidelines and Forms, various. Food and Drugs Authority. Drugs, Biologics, and Devices | domain |
| Greek Constitution 1975/1986/2001, Article 5.5. National Bioethics Commission (NBC). Genetic Research | document type |
| Code of Practice on the Identity Card Number and Other Personal Identifiers (2016). Privacy Commissioner for Personal Data, Hong Kong e eHealth Electronic Health Record Sharing System. Privacy/Data Protection | domain |
| Human Reproductive Technology (Amendment) Ordinance 2016. Legislative Council of the Hong Kong Special Administrative Region of the People’s Republic of China. Embryos, Stem Cells, and Cloning | duplicate |
| Biobanks Act No. 110/2000 (2015). Ministry of Health e National Bioethics Committee (NBC). Human Biological Materials | domain |
| Regulation on Clinical Trials of Medicinal Products in Humans No. 443/2004 (2010). Icelandic Health Insurance Agency (MCA). Research Injury | duplicate |
| Association between vaccination rates and COVID-19 health outcomes in the United States: a population-level statistical analysis | domain |
| CDSCO, Drugs and Cosmetics Act (1940 amended up to 31st December, 2016). Central Drugs Standard Control Organization (CDSCO), Office of Drugs Controller General of India (DCGI). Drugs, Biologics, and Devices | domain |
| Clinical Trials Registry – India, FAQs. Indian Council of Medical Research (ICMR). Clinical Trial Registries | document type |
| Recommendations for successful involvement of patient partners in complex intervention research: a collaborative learning process | duplicate |
| DBT, Environmental Protection Act (1986). Department of Biotechnology (DBT). Genetic Research | domain |
| DBT, Recombinant DNA Safety Guidelines (1990). Department of Biotechnology (DBT). Genetic Research | domain |
| DBT, Regulations and Guidelines for Recombinant DNA Research and Biocontainment (2017). Department of Biotechnology (DBT). Genetic Research | domain |
| Govt. of India Office Memorandum (O.M. No.19015/53/1997 - IH Pt.) 19th November, 1997 on Exchange of Human Biological Material for Biomedical Research Purposes. Indian Council of Medical Research (ICMR). Human Biological Materials | domain |
| ICMR and DBT Combined, National Guidelines for Stem Cell Research (2017). Indian Council of Medical Research (ICMR) e Department of Biotechnology (DBT). Embryos, Stem Cells, and Cloning | duplicate |
| Additional Protocol I Relating to the Protection of Victims of International Armed Conflicts, Article 11 (1977). International Commitee of the Red Cross (ICRC). General | domain |
| International Covenant on Civil and Political Rights, Article 7 (1976). Office of the United Nations High Commissioner for Human Rights (OHCHR). General | domain |
| Recommendation Rec(2016) 8 of the Committee of Ministers to Member States on the Processing of Personal Health-Related Data for Insurance Purposes, Including Data Resulting from Genetic Tests (2016). Council of Europe, Bioethics Unit. Genetic Research | domain |
| Managing Ethical Issues in Infectious Disease Outbreaks: Guidance Document (2016). World Health Organization (WHO). General | domain |
| Statement on Gene Therapy Research (2001). Human Genome Organization. Genetic Research | domain |
| Good Participatory Practice: Guidelines for Biomedical HIV Prevention Trials (2011). UNAIDS. General | domain |
| Guidelines for the Conduct of Human Embryonic Stem Cell Research (2006). International Society for Stem Cell Research. Embryos, Stem Cells, and Cloning | duplicate |
| Guidelines for the Safe Transport of Infectious Substances and Diagnostic Specimens (1997). World Health Organization. Human Biological Materials | domain |
| Infectious Substances and Diagnostic Specimens Shipping Guidelines (2005). International Air Transport Association. Human Biological Materials | domain |
| International Declaration on Human Genetic Data: Section 22 of Major Programme III – Social and Human Sciences (2003). UNESCO Bioethics Program. Genetic Research | domain |
| International Ethical Guidelines for Health-related Research Involving Humans (2016), Guideline 14. Council for International Organizations of Medical Sciences. Research Injury | duplicate |
| ISBER Best Practices: Recommendations for Repositories (2019) and Addendums. International Society for Biological and Environmental Repositories. Human Biological Materials | domain |
| Operational Guidance: Information Needed to Support Clinical Trials of Herbal Products (2005). World Health Organization (WHO). Drugs, Biologics, and Devices | domain |
| Statement on DNA Sampling: Control and Access (1998). Human Genome Organization. Genetic Research | domain |
| Statement on Human Genomic Databases (2002). Human Genome Organization. Genetic Research | domain |
| Statement on the Principled Conduct of Genetic Research (1996). Human Genome Organization. Genetic Research | domain |
| Various Archived Documents from the Global Harmonization Task Force (GHTF), replaced by the IMDRF in 2012. International Medical Device Regulators Forum (IMDRF). Drugs, Biologics, and Devices | document type |
| DPC, For Organisations. Data Protection Commissioner (DPC). Privacy/Data Protection | document type |
| DPC, International Transfers. Data Protection Commissioner (DPC). Privacy/Data Protection | document type |
| Ethics of Procuring and Using Organs or Tissue from Infants and Newborns for Transplantation, Research, or Commercial Purposes: Protocol for a Bioethics Scoping Review | duplicate |
| HRB, Health Research Regulations 2018 FAQ. Health Research Board (HRB). Privacy/Data Protection | document type |
| Human Biological Material: Recommendations for Collection, Use, and Storage in Research (2005). Health Products and Regulatory Authority. Human Biological Materials | domain |
| Irish Medicines Board, Guidelines for Pharmacogenetic Research (2006). Health Products and Regulatory Authority. Genetic Research | domain |
| Summary on Clinical Trials Involving Medical Products. Department of Health e Health Products and Regulatory Authority. Drugs, Biologics, and Devices | document type |
| Various. Department of Health e Health Products and Regulatory Authority. Drugs, Biologics, and Devices | document type |
| Genetic Information Law (2000). Ministry of Health. Genetic Research | domain |
| Guidelines, various. The Privacy Protection Authority. Privacy/Data Protection | domain |
| Legislations, various. The Privacy Protection Authority. Privacy/Data Protection | domain |
| Food and Drugs Act (1975). Ministry of Health, Standards and Regulation Division. Drugs, Biologics, and Devices | domain |
| Food and Drugs Regulations (1975). Ministry of Health, Standards and Regulation Division. Drugs, Biologics, and Devices | domain |
| Act on the Protection of Personal Information (2020). Personal Information Protection Commission e Office of Healthcare Policy of the Cabinet Secretariat. Privacy/Data Protection | language |
| NIPH Clinical Trials Search. National Institute of Public Health. Clinical Trial Registries | document type |
| HIV and AIDS Prevention and Control Act, Chapter 14 (2006). National Council for Science and Technology (NCST) e Ministry of Health (MOH). General | domain |
| Kenya National Guidelines for Research and Development of HIV/AIDS Vaccines, page 44 (2005). Ministry of Health (MOH). Human Biological Materials | domain |
| Medicaid Expansion of the Patient Protection and Affordable Care Act and Participation of Patients With Medicaid in Cancer Clinical Trials | duplicate |
| Pharmacy and Poisons Act, Chapter 244 (2009). Pharmacy and Poisons Board. Drugs, Biologics, and Devices | domain |
| Science and Technology Act (2001). National Council for Science and Technology (NCST) e Ministry of Health (MOH). General | domain |
| PAHO, Regional Program on Bioethics, various resources. Pan American Health Organization. General | document type |
| PAHO, Working Group on Good Clinical Practices, various documents. Pan American Health Organization (PAHO). Drugs, Biologics, and Devices | document type |
| Human Genome Research Law (2005). Ministry of Health, Data State Inspectorate e Central Medical Ethics Committee. Genetic Research | domain |
| Law on Pharmacy, Section 26 (2013). State Agency of Medicines e Central Medical Ethics Committee. Drugs, Biologics, and Devices | domain |
| Law on the Development and Use of the National DNA Database (2006). Ministry of Health, Data State Inspectorate e Central Medical Ethics Committee. Genetic Research | domain |
| Law on the Protection of the Body of Deceased Human Beings and the Use of Human Tissues and Organs in Medicine (2008). Central Medical Ethics Committee. Human Biological Materials | domain |
| Regulation (EU) No. 536/2014 of the European Parliament and of the Council of 16 April 2014 on Clinical Trials on Medicinal Products for Human Use (Effective 31 January 2022). Ministry of Health (MOH) e State Health Care Accreditation Agency Under the Ministry of Health (SHCA). Drugs, Biologics, and Devices | duplicate |
| Clinical trials, Regulation (EU) No 536/2014. Health Ministry, Health Directorate, National Research Ethics Committee (CNER) e Division of Pharmacy and Medicines of the Ministry of Health. Drugs, Biologics, and Devices | duplicate |
| Medical Devices, Regulation (EU) 2017/745. Health Ministry, Health Directorate, National Research Ethics Committee (CNER) e Division of Pharmacy and Medicines of the Ministry of Health. Drugs, Biologics, and Devices | domain |
| Vulnerability, social value and the equitable sharing of benefits from research: beyond the placebo and access debates | domain |
| Constitution of Malawi, Article 19(5) (1994). National Commission for Science and Technology (NCST), National Health Sciences Research Committee (NHSRC), College of Medicine Research and Ethics Committee (COMREC) e Ministry of Health. General | document type |
| National Regulatory Requirement and Position on Accessing, Collection, Storage, and Use of Human Biological Specimens for Research (2014). National Commission for Science and Technology. Human Biological Materials | domain |
| Pharmacy, Medicines, and Poisons Act, Act 15 of 1988. Pharmacy, Medicines, and Poisons Board of Malawi. Drugs, Biologics, and Devices | domain |
| Safer not to know? Shaping liability law and policy to incentivize adoption of predictive AI technologies in the food system | domain |
| Presidential Decree on 30th March 1974. National Commission for Science and Technology (NCST), National Health Sciences Research Committee (NHSRC), College of Medicine Research and Ethics Committee (COMREC) e Ministry of Health. General | domain |
| Procedures and Guidelines for Access and Collection of Genetic Resources in Malawi (2002). National Research Council of Malawi (NRCM). Genetic Research | domain |
| Act 130, Human Tissues Act (1974): Section 2 Removal of parts of bodies for therapeutic purpose. National Committee for Clinical Research (NCRC) e Laws of Malaysia. Attorney General’s Chambers of Malaysia (AGC). Human Biological Materials | domain |
| Act 678. Biosafety Act 2007. Malaysian Medical Council, Laws of Malaysia. Attorney General’s Chambers of Malaysia (AGC), Medical Development Division, Ministry of Health (MOH) e Ministry of Energy and Natural Resources. Genetic Research | domain |
| Act 699, DNA Identification Act 2009. Malaysian Government Gazette of 3 September 2009. National Committee for Clinical Research (NCRC) e Laws of Malaysia. Attorney General’s Chambers of Malaysia (AGC). Human Biological Materials | domain |
| Act 795 Access to Biological Resources and Benefit Sharing Act (2017). National Committee for Clinical Research (NCRC) e Laws of Malaysia. Attorney General’s Chambers of Malaysia (AGC). Human Biological Materials | domain |
| Biosafety (Approval and Notification) Regulations 2010. Malaysian Medical Council, Laws of Malaysia. Attorney General’s Chambers of Malaysia (AGC), Medical Development Division, Ministry of Health (MOH) e Ministry of Energy and Natural Resources. Genetic Research | domain |
| Malaysian Guideline on the Use of Human Biological Sample for Research (2015). National Committee for Clinical Research (NCRC) e Laws of Malaysia. Attorney General’s Chambers of Malaysia (AGC). Human Biological Materials | domain |
| Medical Treatment Law, Section 34 (2014). State Agency of Medicines. Drugs, Biologics, and Devices | domain |
| Medical Device (Exemption) Order 2016. Ministry of Health Malaysia, National Pharmaceutical Regulatory Agency (NPRA), National Committee for Clinical Research (NCRC), Clinical Research Malaysia (CRM), Ministry of Health e Society of Clinical Research Professionals Malaysia (SCRPM). Drugs, Biologics, and Devices | domain |
| Medical Device Act 2012. Medical of Health Malaysia, National Pharmaceutical Regulatory Agency (NPRA), National Committee for Clinical Research (NCRC), Clinical Research Malaysia (CRM), Ministry of Health e Society of Clinical Research Professionals Malaysia (SCRPM). Drugs, Biologics, and Devices | domain |
| Medical Device Authority Act 2012. Ministry of Health Malaysia, National Pharmaceutical Regulatory Agency (NPRA), National Committee for Clinical Research (NCRC), Clinical Research Malaysia (CRM), Ministry of Health e Society of Clinical Research Professionals Malaysia (SCRPM). Drugs, Biologics, and Devices | domain |
| Medical Device Guidance Document Notification of Exemption from Registration of Medical Devices For The Purpose Of Clinical Research Or Performance Evaluation (Medical Device Guidance) (2017). Ministry of Health Malaysia, National Pharmaceutical Regulatory Agency (NPRA), National Committee for Clinical Research (NCRC), Clinical Research Malaysia (CRM), Ministry of Health e Society of Clinical Research Professionals Malaysia (SCRPM). Drugs, Biologics, and Devices | domain |
| Medical Device Regulations 2012. Ministry of Health Malaysia, National Pharmaceutical Regulatory Agency (NPRA), National Committee for Clinical Research (NCRC), Clinical Research Malaysia (CRM), Ministry of Health e Society of Clinical Research Professionals Malaysia (SCRPM). Drugs, Biologics, and Devices | domain |
| MMC, Medical Genetics and Genetic Services. MMC Guidelines 010/2006. Malaysian Medical Council. Genetic Research | domain |
| NMRR, User Manual. National Medical Research Register (NMRR). Clinical Trial Registries | document type |
| User’s Guide to the Access to Biological Resources and Benefit Sharing Act 2017 (Act 795). Malaysian Medical Council, Laws of Malaysia. Attorney General’s Chambers of Malaysia (AGC), Medical Development Division, Ministry of Health (MOH) e Ministry of Energy and Natural Resources. Genetic Research | domain |
| Legal regulation of biomedical research: key principles and their implementation | duplicate |
| Standards, various. Health Authority - Abu Dhabi. General | document type |
| Various Guidelines for Institutional Biosafety Committees. Malaysian Medical Council, Laws of Malaysia. Attorney General’s Chambers of Malaysia (AGC), Medical Development Division, Ministry of Health (MOH) e Ministry of Energy and Natural Resources. Genetic Research | domain |
| Various. Bioethics Committee. General | document type |
| Body Organ Donation Law (2004). Human Biological Materials | domain |
| National Drug Law (1992). Ministry of Health, Food and Drug Administration. Drugs, Biologics, and Devices | domain |
| Various, CCMO Directives. Central Committee for Research Involving Human Subjects (CCMO). General | document type |
| Various, Codes of Conduct. Central Committee for Research Involving Human Subjects (CCMO). General | document type |
| Various, Decrees and Ministerial Regulations. Central Committee for Research Involving Human Subjects (CCMO). General | document type |
| Various, Laws. Central Committee for Research Involving Human Subjects (CCMO). General | document type |
| FAQs. Australian New Zealand Clinical Trials Registry. Clinical Trial Registries | document type |
| Community health workers for health systems resilience during COVID-19 | duplicate |
| Hazardous Substances and New Organisms Act 1996 (2012). Environmental Protection Authority e Health Research Council (HRC), Gene Technology Advisory Committee. Genetic Research | domain |
| Human Tissue Act 2008. Ministry of Health (MOH), Health Research Council (HRC) Ethics Committee, Te Puni Kokiri (TPK), Office of the Health and Disability Commissioner (HDC) e Ministry of Business, Innovation and Employment. Human Biological Materials | domain |
| Medsafe, Medicines Regulations 1984. New Zealand Medicines and Medical Devices Safety Authority (Medsafe). Drugs, Biologics, and Devices | domain |
| MOH, Guidelines for the Use of Human Tissue for Future Unspecified Research Purposes (2007). Ministry of Health (MOH). Human Biological Materials | domain |
| NEAC, National Ethical Standards, various. National Ethics Advisory Committee (NEAC). General | document type |
| Current evidence on the association of tongue strength with cognitive decline in older adults and the known risk factors of frailty, sarcopenia and nutritional health: a scoping review protocol | domain |
| NEAC, Publications and Resources, various. National Ethics Advisory Committee (NEAC). General | document type |
| Official Information Act 1982 (2012). Privacy Commissioner. Privacy/Data Protection | domain |
| Frequently Asked Questions. National Health Research Ethics Committee. Clinical Trial Registries | document type |
| Guides and Forms, various. National Health Research Ethics Committee. General | document type |
| Policy Statement on Storage of Human Samples in Biobanks and Biorepositories in Nigeria (2013). National Health Research Ethics Committee. Human Biological Materials | duplicate |
| Ethical Guidelines for Collection, Usage, Storage, and Export of Human Biological Materials (HBM). National Bioethics Committee. Human Biological Materials. | domain |
| Guidelines For Healthcare Professionals Interaction with Pharmaceutical Trade and Industry (PPI Guidelines). National Bioethics Committee. Drugs, Biologics, and Devices | domain |
| FDA, Circular 2015-026: Adoption of the ICH Harmonized Tripartite Guideline, Quality of Biotechnological Products: Stability Testing of Biotechnological/Biological Products Q5C. Food and Drug Administration (FDA). Drugs, Biologics, and Devices | domain |
| PHREB, Orders and Memoranda, various. Philippine Health Research Ethics Board (PHREB). General | document type |
| Law 12/2005. Ministry of Health. Genetic Research | language |
| Clinical trials, various. Ministry of Public Health, Health Research Governance Department. General | document type |
| Ethical applications of digital community-based research with Black immigrant and refugee youth and families | duplicate |
| IRB Registration and Assurance. Ministry of Public Health, Health Research Governance Department. General | document type |
| Law on Drugs No. 58/08. Ministry of Health and Social Welfare of Republic of Srpska. Drugs, Biologics, and Devices | domain |
| Directive 2010/53/EU of the European Parliament and of the Council of 7 July 2010 on Standards of Quality and Safety of Human Organs Intended for Transplantation. Ministry of Health (MOH). Human Biological Materials | domain |
| Constitution of the Russian Federation, Article 21 (1993). Ministry of Healthcare of the Russian Federation (MOH),Federal Service on Surveillance in Healthcare (Roszdravnadzor) e Russian Committee for Bioethics. General | document type |
| Federal Law No. 61FZ “On Circulation of Medicines” (2011). Ministry of Healthcare of the Russian Federation (MOH), Association of Clinical Trials Organizations e Federal Agency for Technical Regulation and Metrology (GOST). Drugs, Biologics, and Devices | domain |
| Law on Medicines and Medical Devices. Ministry of Health (MOH) e Medicines and Medical Devises Agency of Serbia. General | domain |
| Various rules for medical devices. Ministry of Health (MOH) e Medicines and Medical Devises Agency of Serbia. General | domain |
| Various rules for medicinal products. Ministry of Health (MOH) e Medicines and Medical Devises Agency of Serbia. General | domain |
| Application Guidelines (2017). Sierra Leone Ethics and Scientific Review Committee. General | document type |
| Bioethics Advisory Committee, Human Tissue Research (2002). Ministry of Health (MOH) e Bioethics Advisory Committee (BAC). Human Biological Materials | domain |
| Directive on the Use of Cell, Tissue and Gene Therapy Products Manufactured In-House by Healthcare Institutions (2020). Health Sciences Authority of Singapore (HSA), Ministry of Health (MOH), National Environment Agency (NEA), Centre For Radiation Protection And Nuclear Science. Drugs, Biologics, and Devices | duplicate |
| Genetic Testing and Genetic Research (2005). Bioethics Advisory Committee (BAC). Genetic Research | domain |
| Safeguarding in practice: anticipating, minimising and mitigating risk in teenage pregnancy research in urban informal settlements in Nairobi, Kenya | domain |
| Guidance on Prohibition against Commercial Trading of Human Tissue (2017). Ministry of Health (MOH) e Bioethics Advisory Committee (BAC). Human Biological Materials | domain |
| Health Products (Medical Device) Regulations 2010. Health Sciences Authority of Singapore (HSA), Ministry of Health (MOH), National Environment Agency (NEA), Centre For Radiation Protection And Nuclear Science. Drugs, Biologics, and Devices | domain |
| Human Biomedical Research (Tissue Banking) Regulations 2019. Ministry of Health (MOH) e Bioethics Advisory Committee (BAC). Human Biological Materials | duplicate |
| International Council for Harmonisation of Technical Requirements for Pharmaceuticals for Human Use (ICH), ICH E6(R2) Good Clinical Practice Guideline, 2016, Health Sciences Authority of Singapore (HSA), Ministry of Health (MOH), National Environment Agency (NEA), Centre For Radiation Protection And Nuclear Science. Drugs, Biologics, and Devices | domain |
| Radiation Protection (Non-Ionising Radiation) Regulations 1991. Health Sciences Authority of Singapore (HSA), Ministry of Health (MOH), National Environment Agency (NEA), Centre For Radiation Protection And Nuclear Science. Drugs, Biologics, and Devices | domain |
| Radiation Protection Act 2007. Health Sciences Authority of Singapore (HSA), Ministry of Health (MOH), National Environment Agency (NEA), Centre For Radiation Protection And Nuclear Science. Drugs, Biologics, and Devices | domain |
| Constitution of South Africa, Section 12 (2) (1996). Department of Health (DH), Medical Research Council of South Africa (MRC), Human Sciences Research Council (HSRC) e South African Health Products Regulatory Authority. General | document type |
| FAQs. South African National Clinical Trials Register. Clinical Trials Registry | document type |
| General Regulations Made in Terms of the Medicines and Related Substances Act, 1965 (2003). Department of Health (DH) e Health Products Regulatory Authority. Drugs, Biologics, and Devices | domain |
| Medicines and Related Substances Control Act, 101 of 1965. Department of Health (DH) e Health Products Regulatory Authority. Drugs, Biologics, and Devices | domain |
| MRC, Various Guideline Documents. Medical Research Council of South Africa. General | domain |
| The ethics of infant and early childhood mental health practice | duplicate |
| Regulations Regarding General Control of Human Bodies, Tissues, Blood Products and Gametes, 2 March 2012. Department of Health (DH). Human Biological Materials | domain |
| Regulations Relating to Artificial Insemination of Persons (2016). Department of Health (DH). Human Biological Materials | domain |
| Regulations Relating to Blood and Blood Products, 2 March 2012. Department of Health (DH). Human Biological Materials | domain |
| Regulations Relating to the Use of Human Biological Material, 2 March 2012. Department of Health (DH). Human Biological Materials | domain |
| Ethics in Health Research: Principles, Processes and Structures, Section 3.3.7(i) (2015). Department of Health (DH). Social-Behavioral Research | duplicate |
| Social Media, Public Health Research, and Vulnerability: Considerations to Advance Ethical Guidelines and Strengthen Future Research | domain |
| Enforcement Decree of the Medical Device Act No. 1580 (2019.12.23). Ministry of Food and Drug Safety (MFDS). Drugs, Biologics, and Devices | domain |
| Enforcement Decrees to Personal Information Protection Act No. 30892 (2020.02.04). Ministry of the Interior and Safety (MOIS), Ministry of Health and Welfare (MOHW) e Personal Information Protection Commission (PIPC). Privacy/Data Protection | domain |
| Layered vulnerability and researchers' responsibilities: learning from research involving Kenyan adolescents living with perinatal HIV infection | duplicate |
| Medical Device Act No. 16402 (2019.04.23). Ministry of Food and Drug Safety (MFDS). Drugs, Biologics, and Devices | language |
| Pharmaceutical Affairs Act No. 16250 (2019.01.15). Ministry of Food and Drug Safety (MFDS). Drugs, Biologics, and Devices | domain |
| FAQs. Sri Lanka Clinical Trials Registry. Clinical Trial Registries | document type |
| Guidelines, various. Cosmetics, Devices, and Drugs Regulatory Authority, Subcommittee on Clinical Trials. Drugs, Biologics, and Devices | domain |
| Legislation, various. Cosmetics, Devices, and Drugs Regulatory Authority, Subcommittee on Clinical Trials. Drugs, Biologics, and Devices | domain |
| Defining and assessing psychological frailty in older adults | duplicate |
| NHREC protocol application form. Federal Ministry of Health. General | document type |
| Transmission to Third Countries (2018). Swedish Authority for Privacy Protection. Privacy/Data Protection | domain |
| Ethical considerations in HIV prevention trials. Federal Office of Public Health (FOPH), Federal Office of Public Health, Portal for Human Research (FOPH), National Advisory Commission on Biomedical Ethics (NEK-CNE) e Swiss Association of Research Ethics Committees. General | domain |
| Federal Act of 15 December 2000 on Medicinal Products and Medical Devices (Therapeutic Products Act, TPA), RS 812.21, Articles 53-54. Swiss Agency for Therapeutic Products (Swissmedic) e Federal Office of Public Health (FOPH). Drugs, Biologics, and Devices | domain |
| Frailty Assessment in Vascular OUtpatients Review (FAVOUR) protocol: single-centre prospective cohort study comparing feasibility and prognostic value of commonly used frailty assessment tools | duplicate |
| Federal Act of 30 September 2011 on Research Involving Human Beings (Human Research Act, HRA), RS 810.30. Swiss Agency for Therapeutic Products (Swissmedic) e Federal Office of Public Health (FOPH). Research Injury | language |
| Federal Act of 8 October 2004 on Human Genetic Testing (HGTA), RS 810.12. Federal Office of Public Health (FOPH). Genetic Research | domain |
| Federal Constitution of the Swiss Confederation of 18 April, 1999, RS 101, Article 118b. Federal Office of Public Health (FOPH), Federal Office of Public Health, Portal for Human Research (FOPH), National Advisory Commission on Biomedical Ethics (NEK-CNE) e Swiss Association of Research Ethics Committees. General | document type |
| Ordinance of 20 September 2013 on Clinical Trials in Human Research (Clinical Trials Ordinance ClinO), RS 810.305, Articles 7, 10-13, 25, and 71, and Annexes 2-3. Swiss Agency for Therapeutic Products (Swissmedic) e Federal Office of Public Health (FOPH). Research Injury | language |
| Ordinance of 20 September 2013 on Clinical Trials in Human Research with the Exception of Clinical Trials (Human Research Ordinance, HRO), RS 810.301. Federal Office of Public Health (FOPH), Federal Office of Public Health, Portal for Human Research (FOPH), National Advisory Commission on Biomedical Ethics (NEK-CNE) e Swiss Association of Research Ethics Committees. General | language |
| Ordinance of 20 September 2013 on Human Research with the Exception of Clinical Trials (Human Research Ordinance HRO), RS 810.301, Article 7 (2014). Swiss Agency for Therapeutic Products (Swissmedic) e Federal Office of Public Health (FOPH). Drugs, Biologics, and Devices | duplicate |
| Determinants and acceptability of HIV self-testing among vulnerable groups in sub-Saharan Africa | duplicate |
| Ordinance of 20 September 2013 on Organizational Aspects of the Human Research Act (HRA Organisational Ordinance, OrgO-HRA), RS 810.308. Federal Office of Public Health (FOPH), Federal Office of Public Health, Portal for Human Research (FOPH), National Advisory Commission on Biomedical Ethics (NEK-CNE) e Swiss Association of Research Ethics Committees. General | language |
| Swissmedic Guide to the Regulation of Medical Devices. Swiss Agency for Therapeutic Products (Swissmedic). Drugs, Biologics, and Devices | domain |
| Administrative Regulations on the Establishment of Human Biobanks (2011). Ministry of Health and Welfare (MOHW), Food and Drug Administration (FDA) e Ministry of Science and Technology. Genetic Research | duplicate |
| Human Biobank Management Act (2012). Ministry of Health and Welfare. Human Biological Materials | duplicate |
| Pharmaceutical Affairs Act (2018). Ministry of Health and Welfare (MOHW) e Taiwan Food and Drug Administration (FDA). Drugs, Biologics, and Devices | domain |
| Pharmaceutical Affairs Act Enforcement Rules (2016). Ministry of Health and Welfare (MOHW) e Taiwan Food and Drug Administration (FDA). Drugs, Biologics, and Devices | domain |
| Regulation on Bioavailability and Bioequivalence Studies (2015). Ministry of Health and Welfare (MOHW) e Taiwan Food and Drug Administration (FDA). Drugs, Biologics, and Devices | domain |
| Regulations for Drug Safety Monitoring (2013). Ministry of Health and Welfare (MOHW) e Taiwan Food and Drug Administration (FDA). Drugs, Biologics, and Devices | domain |
| COSTECH, Database, Funded Projects. Tanzania Commission for Science and Technology (COSTECH). Clinical Trials Registry | document type |
| Medical devices, various. Tanzania Medicines and Medical Devises Authority. Drugs, Biologics, and Devices | document type |
| Barriers to Accessing and Engaging in HIV Preventive Care and Pre-Exposure Prophylaxis Experienced by Transgender Women in Florida | duplicate |
| Tanzania Commission for Science and Technology, Act No. 7 of 1986. Ministry of Health (MOH), National Institute for Medical Research (NIMR), National Health Research Ethics Committee (NHREC) e Tanzania Commission for Science and Technology (COSTECH). General | domain |
| Tanzania Food, Drugs, and Cosmetics Act, Sections 61, 66, 67, and 69 (2003). Tanzania Medicines and Medical Devises Authority. Drugs, Biologics, and Devices | domain |
| Addressing the ethical problem of underdiagnosis in the post-pandemic Canadian healthcare system | duplicate |
| FAQs. Thai Clinical Trials Registry. Clinical Trial Registries | document type |
| Laws and Regulations, various. Food and Drug Administration, Medical Device Control Division. Drugs, Biologics, and Devices | domain |
| MCT, Acts and Rules, various. Medical Council of Thailand (MCT). Drugs, Biologics, and Devices | document type |
| NCRT, Regulation on the Permission of Foreign Researchers (1982). National Research Council of Thailand (NCRT). General | domain |
| UWI, Research Ethics, various. University of the West Indies (UWI), St. Augustine. General | document type |
| Good Clinical Practice Guidelines for Advanced Therapy Medicinal Products (2011). Ministry of Health. Human Biological Materials | domain |
| Clinical Trial Application Forms. National Drug Authority. Drugs, Biologics, and Devices | document type |
| Uganda National Council for Science and Technology Act of 1990 (CAP 209). Uganda National Council for Science and Technology (UNCST). General | domain |
| Constitution of Ukraine Art. 28 (1996). Ukrainian Ministry of Health. General | document type |
| Criminal Code of Ukraine 2001, Article 141 and 142. Ukrainian Ministry of Health. General | domain |
| Ionising Radiation (Medical Exposure) (Northern Ireland) Regulations (2018). Department of Health, Social Services and Public Safety e Office for Research Ethics Committees Northern Ireland. General | domain |
| DH, NHS Indemnity Arrangements for Clinical Negligence Claims in the NHS. Department of Health (DH). Research Injury | domain |
| Human Tissue Act (2004) (Applies to England, Wales, and Northern Ireland. Section 45 also applies in Scotland.). Human Tissue Authority (HTA) e Medical Research Council (MRC). Human Biological Materials | domain |
| MHRA, Medicines for Human Use (Clinical Trials) Regulations, Statutory Instrument No. 1031, Regulation 15(5)(i)(j)(k) and Schedule 3 Part 1, Paragraphs 1(g) and 3(c) (2004). Medicines and Healthcare Products Regulatory Agency (MHRA). Research Injury | domain |
| Ethical analysis of cadaver supply and usage processes for research within the scope of the Helsinki Declaration | domain |
| MRC, Human Tissue and Biological Samples for Use in Research (2014). Medical Research Council (MRC). Human Biological Materials | domain |
| Statutory Instrument 2006 No. 1260: The Human Tissue Act 2004 (Ethical Approval, Exceptions from Licensing and Supply of Information about Transplants) Regulations (2006) (Applies to England, Wales, and Northern Ireland.). Human Tissue Authority (HTA) e Medical Research Council (MRC). Human Biological Materials | domain |
| Genetically Modified Organisms (Contained Use) Regulations (Northern Ireland) 2015. Medicines and Healthcare Products Regulatory Agency (MHRA), Administration of Radioactive Substances Advisory Committee (ARSAC) (UK), Department of Environment, Food & Rural affairs (DEFRA), Health and Safety Executive (HSE), Association of the British Pharmaceutical Industry (ABPI), National Institute for Health Research e Health Research Authority (HRA). Drugs, Biologics, and Devices | domain |
| Disparities in Hand Surgery Exist in Unexpected Populations | duplicate |
| Genetically Modified Organisms (Contained Use) Regulations 2014. Medicines and Healthcare Products Regulatory Agency (MHRA), Administration of Radioactive Substances Advisory Committee (ARSAC) (UK), Department of Environment, Food & Rural affairs (DEFRA), Health and Safety Executive (HSE), Association of the British Pharmaceutical Industry (ABPI), National Institute for Health Research e Health Research Authority (HRA). Drugs, Biologics, and Devices | domain |
| Ethics, mental health and freedoms - The contribution of regional ethical reflection spaces | language |
| Genetically Modified Organisms (Deliberate Release) Regulations 2002. Medicines and Healthcare Products Regulatory Agency (MHRA), Administration of Radioactive Substances Advisory Committee (ARSAC) (UK), Department of Environment, Food & Rural affairs (DEFRA), Health and Safety Executive (HSE), Association of the British Pharmaceutical Industry (ABPI), National Institute for Health Research e Health Research Authority (HRA). Drugs, Biologics, and Devices | domain |
| HRA, Medical Devices Guidance. Health Research Authority (HRA). Drugs, Biologics, and Devices | domain |
| ICO, Guide to the General Data Protection Regulation (2018). Information Commissioner’s Office. Privacy/Data Protection | document type |
| ICO, International Transfers (2018). Information Commissioner’s Office. Privacy/Data Protection | document type |
| ISRCTN, FAQs. Clinical Trial Registries | document type |
| Medical Devices (Amendment) Regulations 2008 No. 2936. Medicines and Healthcare Products Regulatory Agency (MHRA) e Health Research Authority (HRA). Drugs, Biologics, and Devices | domain |
| Medical Devices Regulations (2002). Medicines and Healthcare Products Regulatory Agency (MHRA) e Health Research Authority (HRA). Drugs, Biologics, and Devices | domain |
| Medicines Act (1968). Medicines and Healthcare Products Regulatory Agency (MHRA), Administration of Radioactive Substances Advisory Committee (ARSAC) (UK), Department of Environment, Food & Rural affairs (DEFRA), Health and Safety Executive (HSE), Association of the British Pharmaceutical Industry (ABPI), National Institute for Health Research e Health Research Authority (HRA). Drugs, Biologics, and Devices | domain |
| DHSC, Ionising Radiation (Medical Exposure) Regulations (2017). Department of Health and Social Care (DHSC). General | domain |
| Section 251 and the Confidentiality Advisory Group (CAG). Confidentiality Advisory Group (CAG). Privacy/Data Protection | document type |
| Human Tissue (Scotland) Act 2006. Healthcare Improvement Scotland. Human Biological Materials | domain |
| All Common Rule agencies, 45 CFR 46 and applicable subparts. Social-Behavioral Research | document type |
| FDA, Application of Current Statutory Authorities to Human Somatic Cell Therapy Products and Gene Therapy Products. October 14, 1993. 58 FR 53248. FDA, Office of In Vitro Diagnostic Device Evaluation and Safety, FDA, Center for Biologics Research and Evaluation (CBER). Genetic Research | domain |
| FDA, CBER-Specific, Various. FDA, Office of In Vitro Diagnostic Device Evaluation and Safety, FDA, Center for Biologics Research and Evaluation (CBER). Genetic Research | document type |
| FDA, Guidance on Informed Consent for In Vitro Diagnostic Device Studies Using Leftover Human Specimens That are Not Individually Identifiable (2006). FDA, Office of In Vitro Diagnostic Device Evaluation and Safety, FDA, Center for Biologics Research and Evaluation (CBER). Genetic Research | domain |
| FDA, In Vitro Diagnostic (IVD) Device Studies, FAQs (2010). FDA, Office of In Vitro Diagnostic Device Evaluation and Safety, FDA, Center for Biologics Research and Evaluation (CBER). Genetic Research | document type |
| Genetic Information Nondiscrimination Act (GINA) (2008). FDA, Office of In Vitro Diagnostic Device Evaluation and Safety, FDA, Center for Biologics Research and Evaluation (CBER), HHS, Office for Human Research Protections (OHRP), HHS, NIH, Office of Science Policy, Biosafety, Biosecurity, and Emerging Biotechnology Policy Division e HHS, Office for Civil Rights (OCR). Genetic Research | domain |
| HHS, Food and Drug Administration (FDA). General | domain |
| HHS, OCR, Various. Privacy/Data Protection | document type |
| National Science Foundation, FAQs and Vignettes. Social-Behavioral Research | document type |
| KDOQI Commentary on the KDIGO 2022 Update to the Clinical Practice Guideline for Diabetes Management in CKD | domain |
| NIH Guidelines for Research Involving Recombinant or Synthetic Nucleic Acid Molecules (2019). HHS, NIH, Office of Science Policy, Biosafety, Biosecurity, and Emerging Biotechnology Policy Division. Genetic Research | domain |
| A paradigm shift in disaster management: Incorporating a human rights-based approach to disaster risk reduction | duplicate |
| NIH, HIPAA Resources, various. Privacy/Data Protection | document type |
| OCR, HIPAA Privacy Rule Provisions Implementing GINA Requirements at 45 CFR 160.103; 45 CFR 164.502(a)(5)(i); 45 CFR 164.514(g); and 45 CFR 164.520(b)(1)(iii)(C). HHS, Office for Civil Rights (OCR). Genetic Research | domain |
| OHRP, Guidance on the Genetic Information Nondiscrimination Act: Implications for Investigators and Institutional Review Boards (2009). HHS, Office for Human Research Protections (OHRP). Genetic Research | domain |
| OHRP, Research on Transplantation of Fetal Tissue, Public Law 103-43 (1993). HHS, Office for Human Research Protections (OHRP). Genetic Research | duplicate |
| Other relevant standards by U.S. Federal departments and agencies include: Agency for International Development (Protection of Human Subjects in Research Supported by USAID: A Mandatory Reference for ADS Chapter 200 (2015)), Central Intelligence Agency (Executive Order 12333, adopting 45 CFR 46 Subparts A, B, C, and D), Department of Defense, Directorate of Human Research Protections (DOHRP) (United States Code Title 10, Section 980: Limitation on Use of Humans as Experimental Subjects e DoDI 3216.02 (2011)), Department of Education (Protection of Pupil Rights Amendment (1974), Family Educational Rights and Privacy Act (1974), 34 CFR 98 (1984), 34 CFR 99 (2000), 34 CFR 350.4c (1991) e 34 CFR 356.3(c) (1991)), Department of Energy (DOE Order 443.1B e DOE Order 481.1), Department of Homeland Security (Public Law 108-458, Section 8306, DHS Directive 026-04, Human Subjects Research (2007)), Bureau of Prisons (28 CFR 22 Privacy Regulation (1976), 42 U.S.C. § 3789g Confidentiality of Information (1984), 28 CFR 46 (1991), Subpart A), Department of Veterans Affairs (Office of Research Oversight (ORO), Office of Research and Development, 38 CFR 17.85 (1998), VA, Policies, Human Research, various), Environmental Protection Agency, Program in Human Research Ethics (Subpart A: Basic EPA Policy for Protection of Subjects in Human Research Conducted or Supported by EPA (Common Rule), Subpart B: Prohibition of Research Conducted or Supported by EPA Involving Intentional Exposure of Human Subjects who are Children or Pregnant or Nursing Women (2006), Subpart C: Observational Research: Additional Protections for Pregnant Women and Fetuses Involved as Subjects in Observational Research Conducted or Supported by EPA (2006), Subpart D: Observational Research: Additional Protections for Children Involved as Subjects in Observational Research Conducted or Supported by EPA (2006), Subpart K: Basic Ethical Requirements for Third-Party Human Research for Pesticides Involving Intentional Exposure of Non-pregnant, Non-nursing Adults (2013), Subpart L: Prohibition of Third-Party Research Involving Intentional Exposure to a Pesticide of Human Subjects who are Children or Pregnant or Nursing Women (2013), Subpart M: Requirements for Submission of Information on the Ethical Conduct of Completed Human Research (2013), Subpart O: Administrative Actions for Noncompliance (2013), Subpart P: Review of Proposed and Completed Human Research (2013), Subpart Q: Standards for Assessing Whether to Rely on the Results of Human Research in EPA Actions (2013), Scientific and Ethical Approaches for Observational Exposure Studies (2008)), EPA Order 1000.17A: Policy and Procedures on Protection of Human Subjects in EPA Conducted or Supported Research (2016). General | domain |
| European Respiratory Society statement on frailty in adults with chronic lung disease | duplicate |
| FDA, Biologics, Guidance, various. Food and Drug Administration. Drugs, Biologics, and Devices | domain |
| FDA, Drugs, Guidance, various. Food and Drug Administration. Drugs, Biologics, and Devices | domain |
| Food, Drug, and Cosmetic Act, 21 USC Sections 355 and 371 (2012). Food and Drug Administration. Drugs, Biologics, and Devices | domain |
| Personal Information Protection Act (2015). Ministry of Justice. Privacy/Data Protection | domain |
| Department of Veterans Affairs, FAQ. Food and Drug Administration, National Institutes of Health ClinicalTrials e Office of Research Oversight (ORO). Clinical Trial Registries | document type |
| Surveillance towards preventing paediatric incidence of respiratory syncytial virus attributable respiratory tract infection in primary and secondary/tertiary healthcare settings in Merseyside, Cheshire and Bristol, UK | duplicate |
| An integrative decision-making framework to guide policies on regulating ChatGPT usage | domain |
| FAQs on ClinicalTrials.gov. Food and Drug Administration, National Institutes of Health ClinicalTrials e Office of Research Oversight (ORO). Clinical Trial Registries | document type |
| Food and Drug Administration Amendments Act, Section 801 (2007). Food and Drug Administration, National Institutes of Health ClinicalTrials e Office of Research Oversight (ORO). Clinical Trial Registries | domain |
| Assessing Motor Function in Frail Older Adults in Their Home Settings: Challenges, Strategies and Recommendations | duplicate |
| Food and Drug Administration Modernization Act, Section 113 (1997). Food and Drug Administration, National Institutes of Health ClinicalTrials e Office of Research Oversight (ORO). Clinical Trial Registries | domain |
| Model rules of ethical conduct of the Agency for the Development of the Pharmaceutical Industry under the Ministry of Health of the Republic of Uzbekistan (Order No. 90 of July 26, 2018). Center for Expertise and standardization of medicines, medical devices and medical equipment, Ministry of Health, National Ethics Committee e Scientific Boards of Medical Institutes. Drugs, Biologics, and Devices | language |
| Law on Pharmacy (No. 34/2005/QH11), Chapter II (Section III, Article 20), Chapter VIII (Articles 54 and 59) (2005). Ministry of Health. Drugs, Biologics, and Devices | domain |
| Medicines and Allied Substances Act, Part VI: Regulation of Clinical Trials, 2013. Zambia Medicines Regulatory: Authority. Drugs, Biologics, and Devices | domain |
| Constitution of Zimbabwe of 2013, Section 57. Registrar General e Zimbabwe National Statistics Agency. Privacy/Data Protection | document type |
| Medicines and Allied Substances Control Act, Various Regulations. Medicines Control Authority of Zimbabwe. Drugs, Biologics, and Devices | domain |
| National Biotechnology Authority Act, Chapter 14:31 (2006). National Biotechnology Authority of Zimbabwe. Genetic Research | domain |
| Various. Research Council of Zimbabwe. Human Biological Materials | document type |
| African Evaluation Association, African Evaluation Guidelines. Research ethics and social sciences | domain |
| Australian Society of Criminology, Code of Ethics. Research ethics and social sciences | document type |
| Australian Computer Society, Code of Ethics. Research ethics and social sciences | document type |
| Academy of Criminal Justice Science, Code of Ethics. Research ethics and social sciences | document type |
| Canadian Information Processing Society (CIPS), Code of Ethics and Standards of Conduct. Research ethics and social sciences | document type |
| Canadian Psychological Association (2000), Canadian Code of Ethics for Psychologists. Research ethics and social sciences | document type |
| Emphasizing Patient-Centricity Through a Tailored Training Program to Empower Patients, Advocates, and Ethics Committees in Good Clinical Practice | domain |
| Ontario College of Social Workers and Social Service Workers (2000). Code of Ethics and Standards of Practice. Research ethics and social sciences | document type |
| Additional protocol to Convention 108 regarding supervisory authorities and transborder data flows (ETS No. 181). Research involving data processing | domain |
| Commission Decision 2002/364/EC7 on common technical specifications for in-vitro diagnostic medical devices. Research involving medical intervention | domain |
| Commission Directive 2006/17/EC of 8 February 2006 implementing Directive 2004/23/EC of the European Parliament and of the Council as regards certain technical requirements for the donation, procurement and testing of human tissues and cells. Research involving medical intervention | domain |
| Commission Directive 2006/86/EC of 24 October 2006 implementing Directive 2004/23/EC of the European Parliament and of the Council as regards traceability requirements, notification of serious adverse reactions and events and certain technical requirements for the coding, processing, preservation, storage and distribution of human tissues and cells. Research involving medical intervention | domain |
| Commission Recommendation No 2003/556/EC on guidelines for the development of national strategies and best practices to ensure the coexistence of genetically modified crops with conventional and organic farming. Research involving genetic modification | domain |
| Commission Recommendation on a code of conduct for responsible nanosciences and nanotechnologies research. The safety of researchers | domain |
| Convention for the Protection of Human Rights and Fundamental Freedoms (1950). Research involving medical intervention | duplicate |
| Council Directive 96/29/Euratom of 13 May 1996 laying down basic safety standards for the protection of the health of workers and the general public against the dangers arising from ionising radiation. The safety of researchers | domain |
| Council of Europe Recommendation (84) 16 of the Committee of Ministers to member states concerning notification of work involving recombinant deoxyribonucleic acid (DNA). The safety of researchers | domain |
| Council Recommendation of 29 June 1998 on the suitability of blood and plasma donors and the screening of donated blood in the European Community (OJ L 203, 21.7.1998). Research involving medical intervention | domain |
| Directive 2000/14/EC of the European Parliament and of the Council of 8 May 2000 on the approximation of the laws of the Member States relating to the noise emission in the environment by equipment for use outdoors. The safety of researchers | domain |
| The power of connected clinical teams: from loneliness to belonging | domain |
| Directive 2000/54/EC of the European Parliament and of the Council of 18 September 2000 on the protection of workers from risks related to exposure to biological agents at work (seventh individual directive within the meaning of Ar ticle 16(1) of Directive 89/391/EEC ). Research involving data processing | domain |
| Directive 2001/18/EC on the deliberate release into the environment of genetically modified organisms and repealing Council Directive 90/220/EEC. Research involving genetic modification | domain |
| Directive 2002/58/EC of the European Parliament and of the Council of 12 July 2002 concerning the processing of personal data and the protection of privacy in the electronic communications sector. Research involving data processing | domain |
| Directive 2002/98/EC of the European Parliament and of the Council of 27 January 2003 setting standards of quality and safety for the collection, testing, processing, storage and distribution of human blood and blood components. Research involving medical intervention | domain |
| Enforcement Decrees to Personal Information Protection Act No. 30892 (2020.02.04). Ministry of the Interior and Safety (MOIS), Ministry of Health and Welfare (MOHW) e Personal Information Protection Commission (PIPC). Privacy/Data Protection | document type |
| Directive 2003/10/EC of 6 February 2003 on the minimum health and safety requirements regarding the exposure of workers to the risks arising from physical agents (noise) (Seventeenth individual Directive within the meaning of Article 16(1) of Directive 89/391/EEC ). The safety of researchers | domain |
| Directive 2003/122/Euratom of 22 December 2003 on the control of high-activity sealed radioactive sources and orphan sources. The safety of researchers | domain |
| The fragility index in randomized clinical trials supporting clinical practice guidelines for acute coronary syndrome: measuring robustness from a different perspective | duplicate |
| Implementation and evaluation of ultra-low dose CT in early cystic fibrosis lung disease | domain |
| Directive 2004/108/EC of the European Parliament and of the Council of 15 December 2004 on the approximation of the laws of the Member States relating to electromagnetic compatibility and repealing Directive 89/336/EEC. The safety of researchers | domain |
| Directive 2004/40/EC of 29 April 2004 on the minimum health and safety requirements regarding the exposure of the workers to risks arising from electromagnetic fields and waves (18th individual directive within the meaning of Art. 16(1) of directive 89/391/EEC ). The safety of researchers | domain |
| Directive 2006/24/EC of the European Parliament and of the Council of 15 March 2006 on the retention of data generated or processed in connection with the provision of publicly available electronic communications services or of public communications networks and amending Directive 2002/58/EC. Research involving data processing | domain |
| Directive 2006/25/EC on the minimum health and safety requirements regarding the exposure of the workers to risks arising from physical agents (artificial optical radiation, 19th individual directive within the meaning of Article 16(1) of Directive 89/391/EEC ). The safety of researchers | domain |
| Directive 2009/71/Euratom of 25 June 2009 establishing a Community framework for the nuclear safety of nuclear installations. The safety of researchers | domain |
| Directive 90/219/EEC on the contained use of genetically modified micro-organisms. Research involving genetic modification | domain |
| Directive 96/29/Euratom of 13 May 1996 laying down basic safety standards for the protection of the health of workers and the general public against the dangers arising from ionizing radiation. The safety of researchers | domain |
| Development of cancer surveillance guidelines in ataxia telangiectasia: A Delphi-based consensus survey of international experts | duplicate |
| Directive 97/66/EC of the European Parliament and of the Council of 15 December 1997 concerning the processing of personal data and the protection of privacy in the telecommunications sector. Research involving data processing | domain |
| Directive 98/81/EC amending Directive 90/219/EEC on the contained use of genetically modified micro-organisms. Research involving genetic modification | domain |
| European Commission - DG ENTERPISE - Detailed guidance on the European database of Suspected Unexpected Serious Adverse Reactions (EudraVigilance -Clinical Trial Module) (revision 1) as required by Article 11, Article 17 and Article 18 of Directive 2001/20/EC , 2004. Research involving medical intervention | domain |
| European Federation of Professional Psychological Associations (1995), Meta code of ethics. Research ethics and social sciences | document type |
| Recommendation (79) 5 of the Committee of Ministers of the Council of Europe to Member States concerning international exchange and transportation of human substances, adopted on 14 March 1979. Research involving medical intervention | domain |
| Ethics challenges in implementing the International Health Regulations in Pakistan during the COVID-19 pandemic | domain |
| Recommendation (81) 1 of the Committee of Ministers to member states on regulations for automated medical data banks. Research involving medical intervention | domain |
| Recommendation (90) 13 of the Committee of Ministers to member states on prenatal genetic screening, prenatal genetic diagnosis and associated genetic counselling. Research involving medical intervention | duplicate |
| Recommendation (93) 4 of the Committee of Ministers to member states concerning clinical trials involving the use of components and fractionated products derived from human blood or plasma. Research involving medical intervention | domain |
| Recommendation (94) 1 of the Committee of Ministers to member states on human tissue banks. Research involving medical intervention | domain |
| Recommendation (94) 11 of the Committee of Ministers to member states on screening as a tool of preventive medicine. Research involving medical intervention | domain |
| Recommendation R(94) 1 of the Committee of Ministers of the Council of Europe on Human Tissue Banks adopted on 14 March 1994. Research involving medical intervention | domain |
| Regulation (EC) No 1084/2003 on the examination of variations to the terms of a marketing authorisation for medicinal products for human use and veterinary medicinal products granted by a competent authority of a Member State. Research involving medical intervention | domain |
| Regulation (EC) No 1085/2003 on the examination of variations to the terms of a marketing authorisation for medicinal products for human use and veterinary medicinal products falling within the scope of Council Regulation (EEC ) No 2309/93. Research involving medical intervention | domain |
| Regulation (EC) No 1829/2003 on genetically modified food and feed. Research involving genetic modification | domain |
| Regulation (EC) No 1830/2003 on traceability and labelling of genetically modified organisms and the traceability of food and feed products produced f rom genetically modified organisms and amending Directive 2001/18/EC. Research involving genetic modification | domain |
| Estimated distribution of malaria cases among children in sub-Saharan Africa by specified age categories using data from the Global Burden of Diseases 2019 | duplicate |
| Regulation (EC) No 1946/2003 on transboundary movements of genetically modified organisms. Research involving genetic modification | domain |
| Regulation (EC) No 641/2004 on detailed rules for the implementation of Regulation (EC ) No 1829/2003 of the European Parliament and of the Council as regards the application for the authorisation of new genetically modified food and feed. Research involving genetic modification | domain |
| Resolution (78) 29 on harmonisation of legislation of member states relating to removal, grafting and transplantation of human substances. Research involving medical intervention | domain |
| Resolutions and Recommendations of the Committee of Ministers on bioethical matters. Research involving medical intervention | document type |
| ESOMAR Arbitration service. Research ethics and social sciences | document type |
| ESOMAR. How to commission research. Research ethics and social sciences | document type |
| ESOMAR. Maintaining distinctions between marketing research and direct marketing. Research ethics and social sciences | domain |
| ESOMAR. Marketing & opinion research using the Internet. Research ethics and social sciences | domain |
| ESOMAR. Mystery shopping. Research ethics and social sciences | domain |
| ESOMAR. Opinion Polls. Research ethics and social sciences | document type |
| ESOMAR. Tape & video recording & client observation of interviews and group discussions. Research ethics and social sciences | domain |
| Motivators and Barriers to COVID-19 Research Participation at the Onset of the COVID-19 Pandemic in Black Communities in the USA | duplicate |
| Article 29 Working Group - Opinion 5/2005 on the use of location data with a view to providing value-added services, November 2005. Research involving data processing | domain |
| Article 29 Working Group - Opinion 6/2000 on the Human Genome and Privacy, July 2000. Research involving data processing | domain |
| Article 29 Working Group - Recommendation 1/99 on Invisible and Automatic Processing of Personal Data on the Internet Performed by Software and Hardware, February 1999. Research involving data processing | domain |
| Article 29 Working Group - Working document on biometrics, March 2004. Research involving data processing | domain |
| Article 29 Working Group - Working document on data protection issues related to RFID technology, January 2005. Research involving data processing | domain |
| Article 29 Working Group - Working Document on Trusted Computing Platforms and in particular on the work done by the Trusted Computing Group (TCG group), January 2004. Research involving data processing | domain |
| Council for International Organizations of Medical Sciences (CIOMS) in collaboration with the World Health Organization (WHO). International Ethical Guidelines for Biomedical Research Involving Human Subjects (Geneva 2002). Research involving medical intervention | duplicate |
| EGE Opinion n° 11 - 21/07/1998 - Ethical aspects of human tissue banking. Research involving medical intervention | domain |
| EGE Opinion n° 15 - 14/11/2000 - Ethical aspects of human stem cell research and use. Research involving medical intervention | duplicate |
| EGE Opinion n° 4 - 13/12/1994 - The ethical implications of gene therapy. Research involving medical intervention | domain |
| EGE Opinion n°17 - 04/02/2003 - Ethical aspects of clinical research in developing countries. Research involving medical intervention | duplicate |
| EGE Opinion n°18 - 28/07/2003 - Ethical aspects of genetic testing in the workplace. Research involving medical intervention | domain |
| EGE Opinion n°19 - 16/03/2004 - Ethical aspects of umbilical cord blood banking. Research involving medical intervention | duplicate |
| International Conference on Harmonisation of Technical Requirements for Registration of Pharmaceutical for Human Use -ICH Harmonised Tripartite Guideline - Guideline for Good Clinical Practice E6(R1). Research involving medical intervention | domain |
| International Guidelines for Ethical Review of Epidemiological Studies issued by the Council for International Organizations of Medical Sciences (CIOMS). Research involving medical intervention | domain |
| International Sociological Association, Code of Ethics. Research ethics and social sciences | document type |
| International Statistical Institute, Declaration on Professional Ethics. Research ethics and social sciences | document type |
| The Declaration of Inuyama-Human Genome Mapping, Genetic Screening and Gene Therapy issued by the Council for International Organizations of Medical Sciences (CIOMS). Research involving medical intervention | domain |
| UNAIDS guidance document on 'Ethical considerations in HIV preventive vaccine research'. Research involving medical intervention | domain |
| UNESCO International Declaration on Human Genetic Data 2003. Research involving data processing | domain |
| UNESCO Recommendation on the Status of Scientific Researchers of 20 November 1974. Research involving medical intervention | domain |
| Working Document on Online Availability of Electronic Health Records by the International Working Group on Data Protection in Telecommunications. Research involving data processing | domain |
| World Medical Association, Declaration of Helsinki, Ethical Principles for Medical Research involving human subjects (Amended 2000). Research ethics and social sciences | duplicate |
| MRS Code of Conduct and related guidelines. Research ethics and social sciences | document type |
| Guidance Note: How to Apply the MRS Code of Conduct in Employee Research. Research ethics and social sciences | document type |
| Association of Social Science Researchers (New Zealand), Code of Ethics. Research ethics and social sciences | document type |
| 25/5/2000 Optional Protocol to the Convention on the Rights of the Child on the sale of children, child prostitution and child pornography. Research involving children | domain |
| ESOMAR. Internet Privacy Policies and privacy statements. Research ethics and social sciences | domain |
| New Zealand Psychological Society, Code of Ethics. Research ethics and social sciences | document type |
| New Zealand Society of Criminology, Code of Ethics. Research ethics and social sciences | document type |
| New Zealand Statistical Association, Code of Conduct. Research ethics and social sciences | document type |
| Sociological Association of Aotearo - New Zealand, Code of Ethics. Research ethics and social sciences | document type |
| NESH (2001) Guidelines for the inclusion of women in medical research: gender as a variable in all medical research. National Committee for Research Ethics in the Social Sciences and the Humanities, Norway. Research involving medical intervention | language |
| The Fragility of Scientific Rigour and Integrity in "Sped up Science": Research Misconduct, Bias, and Hype and in the COVID-19 Pandemic | duplicate |
| Centre for Research Ethics Göteborg University. Research ethics and social sciences | document type |
| Association of Social Anthropologists of the UK and the Commonwealth. Ethical Guidelines for Good Research Practice. Research ethics and social sciences | domain |
| British Association for Applied Linguistics, Recommendations on Good Practice in  Applied Linguistics. Research ethics and social sciences | domain |
| British Educational Research Association (1992), Ethical Guidelines. Research ethics and social sciences | domain |
| British National Union of Journalists, Code of Conduct. Research ethics and social sciences | document type |
| Evaluating Options and Ethics in Pediatric Dentistry due to Declining Access to Hospital Operating Rooms | domain |
| Commission Decision 2000/520 of 26 July 2000 pursuant to Directive 95/46/EC of the European Parliament and of the Council on the adequacy of the protection provided by the safe harbour privacy principles and related frequently asked questions issued by the US Department of Commerce. Official Journal L 215 , 25/08/2000. Research involving data processing | domain |
| British Psychological Society (2000) Code of Conduct, Ethical Principles and Guidelines, Leicester, BPS. Research ethics and social sciences | document type |
| British Society of Criminology, Code of Research Ethics. Research ethics and social sciences | document type |
| Federal Law #FZ 323 “On Foundations of Protection of Citizen’s Health in the Russian Federation” (2011). Ministry of Healthcare of the Russian Federation (MOH),Federal Service on Surveillance in Healthcare (Roszdravnadzor) e Russian Committee for Bioethics. General | domain |
| British Sociological Association (2003) Statement of Ethical Practice, Durham, BSA. Research ethics and social sciences | document type |
| Society for Applied Anthropology, Statement of Ethical and Professional Responsibilities. Research ethics and social sciences | document type |
| UK Internet Service Providers Association (ISPA), Code of Practice 2002. Research ethics and social sciences | document type |
| British Sociological Association. Authorship guidelines for academic papers. Research ethics and social sciences | domain |
| British Sociological Association. Good professional conduct. Research ethics and social sciences | document type |
| Revised Ethical Guidelines for Educational Research (2004). Research ethics and social sciences | domain |
| Developing the evidence and associated service models to support older adults living with frailty to manage their pain and to reduce its impact on their lives: protocol for a mixed-method, co-design study | duplicate |
| Association of Internet Researchers, Ethical Decision-making and Internet Research. Research ethics and social sciences | domain |
| American Anthropological Association Statement on Ethnography and Institutional Review Boards, 2004. Research ethics and social sciences | domain |
| Ethical guidelines for human research on children and adolescents | domain |
| American Anthropological Association, Code of Ethics. Research ethics and social sciences | document type |
| American Association of University Professors, Statement on Professional Ethics. Research ethics and social sciences | document type |
| Rethinking Vulnerability as a Radically Ethical Device: Ethical Vulnerability Analysis and the EU's "Migration Crisis" | domain |
| American Historical Association, Statement on Standards of Professional Conduct 2003. Research ethics and social sciences | document type |
| American Political Science Association, Guide to Professional Ethics in Political Science. Research ethics and social sciences | domain |
| American Psychological Association (2002). Ethical Principles of Psychologists and Code of Conduct 2002. Research ethics and social sciences | document type |
| American Society for Public Administration, Code of Ethics. Research ethics and social sciences | document type |
| American Sociological Association, Code of Ethics. Research ethics and social sciences | document type |
| American Statistical Association, Ethical Guidelines for Statistical Practice. Research ethics and social sciences | domain |
| APSA Guide to Professional Ethics, Rights and Freedoms, 2008. Research ethics and social sciences | domain |
| American Evaluation Society. Guiding Principles for Evaluators. Research ethics and social sciences | domain |
| Inclusion of functional measures and frailty in the development and evaluation of medicines for older adults | duplicate |
| Association for Computing Machinery (ACM), Code of Ethics and Professional Conduct. Research ethics and social sciences | document type |
| American Evaluation Society. Personnel Evaluation Standards. Research ethics and social sciences | domain |
| International Covenant on Civil and Political Rights. New York (1966). Research involving medical intervention | domain |
| International Covenant on Economic, Social and Cultural Rights. New York (1966). Research involving medical intervention | domain |
| NAFSA: Association of International Educators, Washington DC (formerly National Association of Foreign Student Advisers), Code of Ethics. Research ethics and social sciences | document type |
| National Association for the Practice of Anthropology (NAPA), Ethical Guidelines for Practitioners. Research ethics and social sciences | domain |
| American Evaluation Society. Educational Program Evaluation Standards. Research ethics and social sciences | domain |
| National Association of Social Workers, Code of Ethics. Research ethics and social sciences | document type |
| Ethical Conduct for Research Involving Humans. Chapter 2. Scope and Approach (2014) | duplicate |
| Protection of Human Subjects, Exempt Research (2009) | duplicate |
| Protection of Human Subjects, Expedited Research (1998) | duplicate |
| Frequently Asked Questions and Vignettes | document type |
| Ethical Guidelines for Internet Research (2014) | duplicate |
| Good Research Practice: Observational Studies Conducted Through Participating, Observing, and Recording (2011) | duplicate |
| Implementing Regulations of the Law of Ethics of Research on Living Creatures. Categories of Social-Behavioral Research That do not Require Continuing Review (2016) | duplicate |
| ESCMID-ECMM guideline: diagnosis and management of invasive aspergillosis in neonates and children | domain |
| Guidelines for non-transplant chemotherapy for treatment of systemic AL amyloidosis: EHA-ISA working group | domain |
| Nutritional Assessment in Older Adults : MNA® 25 years of a Screening Tool and a Reference Standard for Care and Research; What Next? | domain |
| The International Xenotransplantation Association consensus statement on conditions for undertaking clinical trials of porcine islet products in type 1 diabetes--chapter 7: Informed consent and xenotransplantation clinical trials | domain |
| Management of older and frail patients with multiple myeloma in the Portuguese routine clinical practice: Deliberations and recommendations from an expert panel of hematologists | domain |
| Toward a geriatric approach to patients with advanced age and cardiovascular diseases: position statement of the EuGMS Special Interest Group on Cardiovascular Medicine | domain |
| Israel Ad Hoc COVID-19 Committee: Guidelines for Care of Older Persons During a Pandemic | domain |
| The Italian document: decisions for intensive care when there is an imbalance between care needs and resources during the COVID-19 pandemic | domain |
| Codesign and implementation of an equity-promoting national health literacy programme for people living with inflammatory bowel disease (IBD): a protocol for the application of the Optimising Health Literacy and Access (Ophelia) process | domain |
| Guidelines for the Provision and Assessment of Nutrition Support Therapy in the Pediatric Critically Ill Patient: Society of Critical Care Medicine and American Society for Parenteral and Enteral Nutrition | domain |
| AGS Position Statement: Making Medical Treatment Decisions for Unbefriended Older Adults | domain |
| Dignity in people with dementia: A concept analysis | domain |
| Perinatal-Neonatal Management of COVID-19 Infection - Guidelines of the Federation of Obstetric and Gynaecological Societies of India (FOGSI), National Neonatology Forum of India (NNF), and Indian Academy of Pediatrics (IAP) | domain |
| Medical Expulsive Therapy for Ureterolithiasis: The EAU Recommendations in 2016 | domain |
| COVID-19 vaccination in children and adolescents aged 5 years and older undergoing treatment for cancer and non-malignant haematological conditions: Australian and New Zealand Children's Haematology/Oncology Group consensus statement | domain |
| Assessing whether COVID-19 patients will benefit from critical care, and an objective approach to capacity challenges during a pandemic: An Intensive Care Society clinical guideline | domain |
| Decree No. 2020-407 of April 22, 2020, Regulating Clinical Trials | language |
| Practical guidance for the management of aromatase inhibitor-associated bone loss | domain |
| Easier said than done: World Health Organization recommendations for prevention of mother-to-child transmission of HIV-areas of concern | domain |
| Normative Standards for HRpQCT Parameters in Chinese Men and Women | domain |
| Carotid Artery Wall Imaging: Perspective and Guidelines from the ASNR Vessel Wall Imaging Study Group and Expert Consensus Recommendations of the American Society of Neuroradiology | domain |
| Advanced (Stage D) Heart Failure: A Statement From the Heart Failure Society of America Guidelines Committee | domain |
| Knowledge Gaps in Cardiovascular Care of Older Adults: A Scientific Statement from the American Heart Association, American College of Cardiology, and American Geriatrics Society: Executive Summary | domain |
| Guidelines for non-transplant chemotherapy for treatment of systemic AL amyloidosis: EHA-ISA working group | domain |
| Canadian Cardiovascular Society/Canadian Association of Interventional Cardiology/Canadian Society of Cardiac Surgery. Position Statement on Revascularization Multivessel Coronary Artery Disease | domain |
| SEOM clinical guidelines for diagnosis and treatment of metastatic colorectal cancer (2018) | domain |
| Human Rights and Inclusion of Vulnerable Groups in Health and Well-Being Policy Documents Relevant to Children and Young People in Ireland | domain |
| ESPEN guideline on clinical nutrition in the intensive care unit | domain |
| Evidence-Based Policy Making: Assessment of the American Heart Association’s Strategic Policy Portfolio | domain |
| Use of Adjuvant Bisphosphonates and Other Bone-Modifying Agents in Breast Cancer: A Cancer Care Ontario and American Society of Clinical Oncology Clinical Practice Guideline | domain |
| Italian association of clinical endocrinologists (AME) position statement: drug therapy of osteoporosis | domain |
| UK clinical guideline for the prevention and treatment of osteoporosis | domain |
| American Society for Pain Management Nursing Position Statement with Clinical Practice Guidelines: Authorized Agent Controlled Analgesia | domain |
| ESPEN guidelines on definitions and terminology of clinical nutrition | domain |
| Clinical Practice Guideline for Postoperative Rehabilitation in Older Patients With Hip Fractures | domain |
| International Clinical Practice Guidelines for Sarcopenia (ICFSR): Screening, Diagnosis and Management | domain |
| AGS Position Statement: Resource Allocation Strategies and Age-Related Considerations in the COVID-19 Era and Beyond | domain |
| Multi-Disciplinary Care Planning of Ovarian Cancer in Older Patients: General Statement—A Position Paper from SOFOG-GINECO-FRANCOGYN-SFPO | domain |
| How to manage recurrent falls in clinical practice: Guidelines of the French society of geriatrics and gerontology | domain |
| Order No. 1250 on the Organization of the National System of Pharmacovigilance (2015) | language |
| Japanese Society for Cancer of the Colon and Rectum (JSCCR) guidelines 2016 for the treatment of colorectal cancer | domain |
| Diagnosis, Treatment, and Prevention of Urinary Tract Infections in Post-Acute and Long-Term Care Settings: A Consensus Statement From AMDA’s Infection Advisory Subcommittee | domain |
| American Society of Clinical Oncology Policy Statement: Opportunities in the Patient Protection and Affordable Care Act to Reduce Cancer Care Disparities | domain |
| American Medical Society for Sports Medicine Position Statement on Concussion in Sport | domain |
| Orthogeriatric co‑management for the care of older subjects with hip fracture: recommendations from an Italian intersociety consensus | domain |
| Assessing whether COVID-19 patients will benefit from critical care, and an objective approach to capacity challenges during a pandemic: An Intensive Care Society clinical guideline | domain |
| Regulating vulnerability: policy approaches for preventing violence and abuse of people with disability in Australian service provision settings | domain |
| Treatment guidelines of metastatic colorectal cancer in older patients from the French Society of Geriatric Oncology (SoFOG) | domain |
| OARSI guidelines for the non-surgical management of knee, hip, and polyarticular osteoarthritis | domain |
| The Belgian Bone Club 2020 guidelines for the management of osteoporosis in postmenopausal women | domain |
| Health Disparities in Patients with Pulmonary Arterial Hypertension: A Blueprint for Action. An Official American Thoracic Society Statement | domain |
| Normative Standards for HRpQCT Parameters in Chinese Men and Women | domain |
| Income Protection for Vulnerable Groups During the Pandemic in Brazil and Chile: The Relevance of Policy Trajectories and Governance Arrangements | duplicate |
| Recommendations on the management of fragility fracture risk in women younger than 70 years | domain |
| [World Allergy Organization Guidelines for the Assessment and Management of Anaphylaxis](https://www.webofscience.com/wos/woscc/full-record/WOS:000288018400005) | domain |
| Postpartum management for gestational diabetes mellitus: Policy and practice implications | domain |
| Best clinical practice guidance for local analgesia in paediatric dentistry. An EAPD policy document | domain |
| Clinical Practice Guidelines for the Prevention and Management of Pain, Agitation/Sedation, Delirium, Immobility, and Sleep Disruption in Adult Patients in the ICU | domain |
| Evidence for anti-osteoporosis therapy in acute fracture situations—Recommendations of a multidisciplinary workshop of the International Society for Fracture Repair | domain |
| [Neurodevelopmental evaluation for school-age children with congenital heart disease: recommendations from the cardiac neurodevelopmental outcome collaborative](https://doi.org/10.1017/S1047951120003546) | domain |
| Guidance to develop individual dose recommendations for patients on chronic hemodialysis | domain |
| Recommendations for a Brief International Cognitive Assessment for Multiple Sclerosis (BICAMS) | domain |
| Health Supervision in the Management of Children and Adolescents With IBD: NASPGHAN Recommendations | domain |
| Recommendations for managing cutaneous disorders associated with advancing age | domain |
| Recommendations for diagnosing and managing individuals with glutaric aciduria type 1: Third revision | domain |
| Recommendations for the prevention, diagnosis, and treatment of chronic hepatitis B and C in special population groups (migrants, intravenous drug users and prison inmates) | domain |
| The American Society of Colon and Rectal Surgeons Clinical Practice Guidelines for the Perioperative Evaluation and Management of Frailty Among Older Adults Undergoing Colorectal Surgery | domain |
| The concept of ‘vulnerability’ in research ethics: an in-depth analysis of policies and guidelines | duplicate |
| Guidelines for the management of osteoporosis and fragility fractures | domain |
| Heat as emergency, heat as chronic stress: policy and institutional responses to vulnerability to extreme heat | domain |
| Assessing Vulnerability in Smart Contracts: The Role of Code Complexity Metrics in Security Analysis | duplicate |
| [Guidelines for Translating Frameworks, Methods, Tools and Principles of Local Innovations for Marginalised and Vulnerable Communities–2023](https://open-research-europe.ec.europa.eu/articles/4-36/v1?src=rss) | duplicate |
| Ethical and Policy Aspects of Frailty | domain |
| Designing out vulnerability, building in respect: violence, safety and sex work policy | document type |
| Two Norms Collide: EU Policy on Fragile and Conflict-Affected Countries | domain |
| Design and evaluation of highly accurate smart contract code vulnerability detection framework | domain |
| The Minnesota Vulnerable Adult Act Policy Analysis of 1995 Reforms | document type |
| Peri-operative ovarian cancer guidelines: anesthesia, intra- and post-operative volume and replacement, post-operative pain management, frailty scores/management of the fragile patient | domain |
| Fragility Index Analysis of the 2018 Clinical Practice Guidelines on Tranexamic Acid Use in Total Joint Arthroplasty | duplicate |
| EMQN best practice guidelines for the molecular genetic testing and reporting of fragile X syndrome and other fragile X-associated disorders | domain |

567 excluded documents
