# Supplementary material for: Vulnerability in research ethics: A systematic review of policy guidelines and documents
Source: PLoS One. 2025 Jul 1;20(7):e0327086. doi: 10.1371/journal.pone.0327086 (PMC12212517; doi:10.1371/journal.pone.0327086)
Supplement: S2 Table — (DOCX) [file pone.0327086.s002.docx]

**S2 Table: Documents excluded from keywords search (no keyword found, or keywords not used as a recurrent concept in the text and in a context relevant to the four research questions)**

| **TITLE** | **VULN-** | **FRAG-** | **FRAIL-** | **NO KEYWORDS** | **NOTES** |
| --- | --- | --- | --- | --- | --- |
| **INTERNATIONAL COMPILATION OF HUMAN RESEARCH STANDARDS (2024 EDITION)** | | | | | |
| AIATSIS, Guidelines for Ethical Research in Australian Indigenous Studies (2012). National Health and Medical Research Council (NHMRC), Australian Research Council (ARC), Australian Institute of Aboriginal and Torres Strait Islander Studies (AIATSIS). General |  |  |  | X |  |
| Australian Clinical Trial Handbook (2018). Therapeutic Goods Administration (TGA). Drugs, Biologics, and Devices |  |  |  | X |  |
| Australian Privacy Principles Guidelines (Combined, 2019). Office of the Australian Information Commissioner. Privacy/Data Protection | X |  |  |  | Not relevant |
| Australian Regulatory Guidelines for Medical Devices (ARGMD) (2011). Therapeutic Goods Administration. Drugs, Biologics, and Devices |  |  |  | X |  |
| Guidelines Approved under Section 95A of the Privacy Act 1988 (2014). Office of the Australian Information Commissioner. Privacy/Data Protection |  |  |  | X |  |
| National Health and Medical Research Council Act 1992 (2014). National Health and Medical Research Council (NHMRC), Australian Research Council (ARC), Australian Institute of Aboriginal and Torres Strait Islander Studies (AIATSIS). General |  |  |  | X |  |
| National Health and Medical Research Regulation 2016. National Health and Medical Research Council (NHMRC), Australian Research Council (ARC), Australian Institute of Aboriginal and Torres Strait Islander Studies (AIATSIS). General |  |  |  | X |  |
| NHMRC, Australian Code for the Responsible Conduct of Research (2018). National Health and Medical Research Council (NHMRC), Australian Research Council (ARC), Australian Institute of Aboriginal and Torres Strait Islander Studies (AIATSIS). General | X |  |  |  | Not relevant |
| NHMRC, Ethical guidelines for research with Aboriginal and Torres Strait Islander Peoples (2018). National Health and Medical Research Council (NHMRC), Australian Research Council (ARC), Australian Institute of Aboriginal and Torres Strait Islander Studies (AIATSIS). General |  |  |  | X |  |
| Privacy Act 1988 (2016). Office of the Australian Information Commissioner. Privacy/Data Protection |  |  |  | X |  |
| Privacy in Australian States and Territories. Office of the Australian Information Commissioner. Privacy/Data Protection |  |  |  | X |  |
| Privacy Regulation 2013 (2016). Office of the Australian Information Commissioner. Privacy/Data Protection |  |  |  | X |  |
| EU General Data Protection Regulation (2016). Privacy/Data Protection | X |  |  |  | Not relevant |
| Act on the Protection of Natural Persons with Regard to the Processing of Personal Data (30 July 2018). Belgian Data Protection Authority. Privacy/Data Protection |  |  |  | X |  |
| Guidelines for Application for Research Permit (2004). Ministry of Health and Wellness. General |  |  |  | X |  |
| Resolution ANVISA 09/15 - Regulations for Clinical Trials with Drugs. Brazilian Health Surveillance Agency (ANVISA). Drugs, Biologics, and Devices |  |  |  | X |  |
| Resolution CNS No. 301, 16th March 2002: Regarding Placebos. National Health Council (CNS). Drugs, Biologics, and Devices |  |  |  | X |  |
| Resolution CNS No. 304/2000: Rules on Research Involving Human Beings – Area of Indigenous Peoples. National Health Council (CNS) e National Commission on Research Ethics (CONEP). General |  |  |  | X |  |
| Resolution RDC No. 9, 20 February 2015 Regarding Regulation for Realization of Clinical Trials of Medication in Brazil. National Health Council (CNS), Brazilian Health Surveillance Agency (ANVISA), Federal Council of Medicine (CFM) e National Commission on Research Ethics (CONEP). Drugs, Biologics, and Devices |  |  |  | X |  |
| Law on Medical Research Involving Humans (2022) | x |  |  |  | Not relevant |
| Consent (2018). Bulgarian Commission for Personal Data Protection e Ombudsman. Privacy/Data Protection |  |  |  | X |  |
| Law for Protection of Personal Data (2018). Bulgarian Commission for Personal Data Protection e Ombudsman. Privacy/Data Protection |  |  |  | X |  |
| Ordinance No. 10 (2008). Bulgarian Drug Agency (BDA). Drugs, Biologics, and Devices | X |  |  |  | Not relevant |
| Personal Data Protection Act. Bulgarian Commission for Personal Data Protection e Ombudsman. Privacy/Data Protection |  |  |  | X |  |
| Operational Guidelines for Ethics Committees in Charge of the Evaluation of Biomedical Research. Cameroon Bioethics Initiative. General |  |  |  | X |  |
| Correctional Service of Canada: Commissioner’s Directive - Research: DCOO9 (2017). Interagency Advisory Panel on Research Ethics (PRE), National Defence and the Canadian Armed Forces e Correctional Service of Canada.General |  |  |  | X |  |
| Health Canada, Good Clinical Practice, Various. Health Canada, Therapeutic Products Directorate e Interagency Advisory Panel on Research Ethics (PRE). Drugs, Biologics, and Devices |  |  |  | X |  |
| Medical Devices Regulations (SOR/98-282) (1998). Health Canada, Medical Device. Drugs, Biologics, and Devices |  |  |  | X |  |
| National Defence and the Canadian Armed Forces, Research Involving Human Subjects (1998). Interagency Advisory Panel on Research Ethics (PRE), National Defence and the Canadian Armed Forces e Correctional Service of Canada.General |  |  |  | X |  |
| Privacy Act, Sections 7-8 (1983). Office of the Privacy Commissioner of Canada (OPC), Interagency Advisory Panel on Research Ethics (PRE) e Canadian Institutes of Health Research (CIHR). Privacy/Data Protection |  |  |  | X |  |
| The Safeguarding and Protection of Patients’ Rights Law (2004). General |  |  |  | X |  |
| Act No. 110/2019 Coll., On Personal Data Processing. Office for Personal Data Protection. Privacy/Data Protection |  |  |  | X |  |
| Act No. 130/2002 Collection on Research and Development Support, as Amended (2018). Ministry of Health, Central Ethics Committee. General |  |  |  | X |  |
| Act No. 372/2011 on Healthcare Services, As Amended (2019). Ministry of Health, Central Ethics Committee. General |  |  |  | X |  |
| CIHR Best Practices for Protecting Privacy in Health Research (2005). Office of the Privacy Commissioner of Canada (OPC), Interagency Advisory Panel on Research Ethics (PRE) e Canadian Institutes of Health Research (CIHR). Privacy/Data Protection | X |  |  |  | Not relevant |
| Decree No. 226/2008 on Good Clinical Practices and on Detailed Conditions for Evaluation of Pharmaceutical Products. Ministry of Health (MOH), State Institute for Drug Control (SUKL). Drugs, Biologics, and Devices |  |  |  | X |  |
| International Data Transfer (2018). Office for Personal Data Protection. Privacy/Data Protection |  |  |  | X |  |
| Guidelines for Applications for Authorisation of Clinical Trials of Medical Products in Humans(2021). Committees on Medicine Research Ethics (VMK) e Danish Medicines Agency. Drugs, Biologics, and Devices |  |  |  | X |  |
| Conditions and Procedure for Conducting Clinical Trials of Medicinal Products. Regulation No. 23 of the Minister of Social Affairs of (2005). Minister of Social Affairs (MSA) e Estonian Health Insurance Fund. Research Injury |  |  |  | X |  |
| Council of Europe Committee on Bioethics Guide for research ethics committee members. Council of Europe, Bioethics Unit. Research Injury | X |  |  |  | Not relevant |
| AMA Policies and Code of Medical Ethics' Opinions Related to Health Care for Patients Who Are Immigrants, Refugees, or Asylees | X |  |  |  | Not relevant |
| MSA, Conditions and Procedure for Conducting Clinical Trials of Medicinal Products. Regulation No. 23 (2005). State Agency of Medicines, Minister of Social Affairs (MSA). Drugs, Biologics, and Devices |  |  |  | X |  |
| MSA, Rules of Procedure of Medical Ethics Committee for Clinical Trials, a List of Data to be Submitted for Obtaining Approval, Procedure for Adoption of Resolutions and Format of Application for Obtaining Approval (2005). State Agency of Medicines, Minister of Social Affairs (MSA). Drugs, Biologics, and Devices |  |  |  | X |  |
| Personal Data Protection Act (2016). Estonian Data Protection Inspectorat. Privacy/Data Protection |  |  |  | X |  |
| Article 29 Working Party Documentation. Council of Europe, Data Protection and Cybercrime Division. Privacy/Data Protection |  |  |  | X |  |
| Clinical Trials Directive 2001/20/EC. European Commission, DG SANTE: Directorate-General for Health and Food Safety.Research Injury | X |  |  |  | Not relevant |
| Convention for the Protection of Individuals with Regard to Automatic Processing of Personal Data (1981). Council of Europe, Data Protection and Cybercrime Division. Privacy/Data Protection |  |  |  | X |  |
| Directive 2001/20/EC on the Approximation of the Laws, Regulations and Administrative  Provisions of the Member States Relating to the Implementation of Good Clinical Practice in the Conduct of Clinical Trials on Medicinal Products for Human Use. European Commission, DG SANTE: Directorate-General for Health and Food Safety. Drugs, Biologics, and Devices | X |  |  |  | Not relevant |
| Directive 2005/28/EC Laying Down Principles and Detailed Guidelines for Good Clinical Practice as Regards Investigational Medicinal Products for Human Use, as Well as the Requirements for Authorization of the Manufacturing or Importation of Such Products. European Commission, DG SANTE: Directorate-General for Health and Food Safety. Drugs, Biologics, and Devices |  |  |  | X |  |
| Directive 2007/47/EC of the European Parliament and of the Council of 5 September 2007  Amending Council Directive 90/385/EEC on Approximation of the Laws of the Member States Relating to Active Implantable Medical Devices. European Medicines Agency. Drugs, Biologics, and Devices | X |  |  |  | Not relevant |
| Directive 93/42/EEC Concerning Medical Devices. European Medicines Agency. Drugs, Biologics, and Devices | X |  |  |  | Not relevant |
| EudraLex Volume 10: Clinical Trials. European Commission, DG SANTE: Directorate-General for Health and Food Safety. Drugs, Biologics, and Devices | X |  |  |  | Not relevant |
| European Commission, Research and Innovation, Law and Regulations. European Commission, European Group on Ethics in Science and New Technologies (EGE) e European Commission, Directorate-General for Research and Innovation. General |  |  |  | X |  |
| European Medicines Agency policy on publication of clinical data for medicinal products for human use. European Medicines Agency (EMA). Privacy/Data Protection |  |  |  | X |  |
| External Guidance on the Implementation of the European Medicines Agency Policy on the Publication of Clinical Data for Medicinal Products for Human Use (2016). European Medicines Agency (EMA). Privacy/Data Protection |  |  |  | X |  |
| Guidelines on consent under Regulation 2016/679, WP259 rev.01. European Data Protection Board (EDPB). Privacy/Data Protection | X |  |  |  | Not rilevant |
| Guidelines on Good Clinical Practice Specific to Advanced Therapy Medicinal Products. European Commission, European Group on Ethics in Science and New Technologies. Human Biological Materials |  |  |  | X |  |
| Protocol amending the Convention for the Protection of Individuals with regard to Automatic Processing of Personal Data (2018). Council of Europe, Data Protection and Cybercrime Division. Privacy/Data Protection | X |  |  |  | Not rilevant |
| Recommendation CM/Rec(2019)2 of the Committee of Ministers to member States on the protection of health-related data (2019). Council of Europe, Data Protection and Cybercrime Division. Privacy/Data Protection |  |  |  | X |  |
| Recommendation No. R (97) 5 on the Protection of Medical Data (1997). Council of Europe, Data Protection and Cybercrime Division. Privacy/Data Protection |  |  |  | X |  |
| Research Ethics Policy and Guidelines. University of the West Indies – Cave Hill / Ministry of Health. General | X |  |  |  | Not relevant |
| Regulation (EC) No. 1394/2007 on Advanced Therapy Medicinal Products and Amending Directive 2001/83/EC and Regulation (EC) No. 726/2004. European Medicines Agency. Genetic Research |  |  |  | X |  |
| Regulation (EU) 2016/679 of the European Parliament and of the Council of 27 April 2016 on the Protection of Natural Persons with Regard to the Processing of Personal Data and on the Free Movement of Such Data, and Repealing Directive 95/46/EC (General Data Protection Regulation). European Data Protection Board (EDPB). Privacy/Data Protection | X |  |  |  | Not relevant |
| Transfers of Personal Data to Third Countries: Applying Articles 25 and 26 of the EU Data Protection Directive (1998). European Data Protection Board (EDPB). Privacy/Data Protection | X |  | X |  | Not relevant |
| Working Document on Adequacy Referential (2018). European Data Protection Board (EDPB). Privacy/Data Protection |  |  |  | X |  |
| Act on Data Protection (1050/2018). Ministry of Social Affairs and Health, National Committee on Medical Research Ethics (TUKIJA), Finnish Advisory Board on Research Integrity (TENK), Finnish Institute for Health and Welfare (THL), Findata e Finnish Medicines Agency Fimea. General |  |  |  | X |  |
| Decree on Clinical Trials on Medicinal Products No. 841/2010. Finnish Medicines Agency (FIMEA), Ministry of Social Affairs and Health (MSAH), National Committee on Medical Research Ethics (TUKIJA) e Regional Medical Ethics Committees. Drugs, Biologics, and Devices |  |  |  | X |  |
| Finnish Medicines Agency Administrative Regulation on Clinical Trials on Medicinal Products (8/2019). Finnish Medicines Agency (FIMEA), Ministry of Social Affairs and Health (MSAH), National Committee on Medical Research Ethics (TUKIJA) e Regional Medical Ethics Committees. Drugs, Biologics, and Devices |  |  |  | X |  |
| Medical Research Act No. 488/1999 (Amended 295/2004, 794/2010, 143/2015 and one related to a Government Proposal to the Parliament HE 18/2020vp in relation to the application of EU Clinical Trials Regulation 536/2014) upcoming). Ministry of Social Affairs and Health, National Committee on Medical Research Ethics (TUKIJA), Finnish Advisory Board on Research Integrity (TENK), Finnish Institute for Health and Welfare (THL), Findata e Finnish Medicines Agency Fimea. General |  |  |  | X |  |
| Pharmaceutical Injuries Insurance: General Terms and Conditions (2017). Finnish Patient Insurance Centre e Pharmaceutical Injuries Insurance. Research Injury |  |  |  | X |  |
| The Ethical Principles of Research with Human Participants and Ethical Review in the Human Sciences in Finland (2019). Ministry of Social Affairs and Health, National Committee on Medical Research Ethics (TUKIJA), Finnish Advisory Board on Research Integrity (TENK), Finnish Institute for Health and Welfare (THL), Findata e Finnish Medicines Agency Fimea. General |  |  |  | X |  |
| Law on Data Protection (2018). Office of the Personal Data Protection Inspector. Privacy/Data Protection |  |  |  | X |  |
| Order of Health Minister about Implementation of “ICH: E6 Good Clinical Practice: Consolidated Guidance” (1996). State Regulatory Agency for Medical and Pharmaceutical Activities (LEPL) of the Ministry of Internally Displaced Persons from the Occupied Territories, Labor, Health and Social Affairs of Georgia. Drugs, Biologics, and Devices |  |  |  | X |  |
| Regulation about the Rules and Conditions of Issuing of the Approval of Clinical Trials Approved #176 (2005). State Regulatory Agency for Medical and Pharmaceutical Activities (LEPL) of the Ministry of Internally Displaced Persons from the Occupied Territories, Labor, Health and Social Affairs of Georgia. Drugs, Biologics, and Devices |  |  |  | X |  |
| Guidelines on Ethical Issues in Public Health Surveillance (2017). World Health Organization (WHO). General | X |  |  |  | Not relevant |
| Medicinal Products Act, Division 6 (2020). Federal Institute for Drugs and Medical Devices (BfArM), Paul-Ehrlich-Institut (PEI) e Federal Ministry of Health (BMG). Drugs, Biologics, and Devices |  |  |  | X |  |
| Act 851, Public Health Act, 2012. Food and Drugs Authority. Drugs, Biologics, and Devices |  |  |  | X |  |
| Applications for Clinical Trials as Defined Under Section 150-166 (Part 8) of the Public Health Act 2012, Act 851. Food and Drugs Authority. Drugs, Biologics, and Devices |  |  |  | X |  |
| Medical Research Involving Human Subjects Regulations (2007). Ministry of Health. General | X |  |  |  | Not relevant |
| Code of Practice on Human Resource Management (2016). Privacy Commissioner for Personal Data, Hong Kong e eHealth Electronic Health Record Sharing System. Privacy/Data Protection | X |  |  |  | Not relevant |
| Personal Data (Privacy) Ordinance (2018). Privacy Commissioner for Personal Data, Hong Kong e eHealth Electronic Health Record Sharing System. Privacy/Data Protection |  |  |  | X |  |
| Act on Health Insurance No. 112/2008 (2012). Icelandic Health Insurance Agency (MCA). Research Injury |  |  |  | X |  |
| Act on Medical Devices No. 16/2001 (2011). Ministry of Health. Drugs, Biologics, and Devices |  |  |  | X |  |
| Act on Patient Insurance No. 111/2000 (2011). Icelandic Health Insurance Agency (MCA). Research Injury |  |  |  | X |  |
| Act on Scientific Research in the Health Sector No. 44/2014. Ministry of Health e National Bioethics Committee (NBC). General | X |  |  |  | Not relevant |
| MCA, Regulation on Clinical Trials of Medicinal Products in Humans No. 443/2004 (2010). Icelandic Medicines Agency (MCA). Drugs, Biologics, and Devices |  |  |  | X |  |
| CDSCO, New Drugs and Clinical Trials Rules (2019). Central Drugs Standard Control Organization (CDSCO), Office of Drugs Controller General of India (DCGI). Drugs, Biologics, and Devices | X |  |  |  | Not relevant |
| Clinical Trials Registry – India. Indian Council of Medical Research (ICMR). Clinical Trial Registries |  |  |  | X |  |
| NACO, Data Protection Guidelines of the National AIDS Control Programme. National AIDS Control Organization (NACO). Privacy/Data Protection |  |  |  | X |  |
| Guidelines on Good Clinical Practice (2001). National Agency of Drug and Food Control. Drugs, Biologics, and Devices |  |  |  | X |  |
| Indonesian Health Act No. 23/1992 Section on Health Research, Article 69. Ministry of Health, National Institute of Health Research and Development. General |  |  |  | X |  |
| Ethical Issues in Patient Safety Research: Interpreting Existing Guidance (2013). World Health Organization (WHO). General | X |  |  |  | Not relevant |
| Geneva Convention Relative to the Treatment of Prisoners of War, Articles 13 and 130 (1950). International Commitee of the Red Cross (ICRC). General |  |  |  | X |  |
| Global Code of Conduct for Research in Resource-Poor Settings (2018). TRUST Project. General |  |  |  | X |  |
| Resolution WHA 58.34 (2005). World Health Organization – International Clinical Trials Registry Platform. Clinical Trial Registries |  |  |  | X |  |
| Clinical Investigation of Medical Devices for Human Subjects -- Good Clinical Practice. Standard Number 14155:2011. International Standards Organization. Drugs, Biologics, and Devices | X |  |  |  | Not relevant |
| Clinical Trial Registration. International Committee of Medical Journal Editors. Clinical Trial Registries |  |  |  | X |  |
| Code of Conduct and Ethical Guidelines for Social Science Research. UNESCO. Social-Behavioral Research |  |  |  | X |  |
| Declaration of Taipei (2016). World Medical Association. Human Biological Materials |  |  |  | X |  |
| Brazilian and Portuguese guidelines for protecting vulnerable children against violence in the COVID-19 pandemic | X |  |  |  | Not relevant |
| Statement Regarding Use of ISO 14155:2011 “Clinical Investigation of Medical Devices for Human Subjects-Good Clinical Practice” (2015). International Medical Device Regulators Forum (IMDRF). Drugs, Biologics, and Devices |  |  |  | X |  |
| Trial Registration. Iranian Registry of Clinical Trials. Clinical Trial Registries |  |  |  | X |  |
| Data Protection Act 2018. Data Protection Commissioner (DPC) e Health Research Board (HRB). Privacy/Data Protection |  |  |  | X |  |
| European Communities (Clinical Trials on Medicinal Products for Human Use) Amendment 2004 (S.I. No. 190 of 2004). Department of Health e Health Products and Regulatory Authority. Drugs, Biologics, and Devices |  |  |  | X |  |
| Health Service Executive National Consent Policy. Department of Health. General | X |  | X |  | Not relevant |
| Operational Procedures for Research Ethics Committees: Guidance 2004. Department of Health. General | X |  |  |  | Not relevant |
| Guidelines for Clinical Trials in Human Subjects (2006). Ministry of Health, Pharmaceutical Administration. Drugs, Biologics, and Devices |  |  |  | X |  |
| Ministry of Health Guidelines for the Conduct of Research on Human Subjects (2010). Ministry of Health, Ethics and Medico-Legal Affairs Panel. General | X |  |  |  | Not relevant |
| Amendment to the Cabinet Order to Enforce the Act on the Protection of Personal Information (2016). Personal Information Protection Commission e Office of Healthcare Policy of the Cabinet Secretariat. Privacy/Data Protection |  |  |  | X |  |
| Enforcement Rules for the Act on the Protection of Personal Information (2016). Personal Information Protection Commission e Office of Healthcare Policy of the Cabinet Secretariat. Privacy/Data Protection |  |  |  | X |  |
| Cabinet Regulation No. 289: Regulations Regarding the Procedures for Conduct of Clinical Trials and Non-interventional Trials of Medicinal Products, Labelling of Investigational Medicinal Products and the Procedures for Assessment of Conformity of Clinical Trial of Medicinal Products with the Requirements of Good Clinical Practice. State Agency of Medicines e Central Medical Ethics Committee. Drugs, Biologics, and Devices |  |  |  | X |  |
| Cabinet Regulation No. 446: Procedures for Using Patient Data in a Specific Research Study (2015). Data State Inspectorate. Privacy/Data Protection |  |  |  | X |  |
| Cabinet Regulation No. 891: Procedures for the Clinical Trial of Medical Devices Intended for Human Use (2010). State Agency of Medicines. Drugs, Biologics, and Devices |  |  |  | X |  |
| Law on the Rights of Patients, Section 10 (2013). Data State Inspectorate. Privacy/Data Protection |  |  |  | X |  |
| Personal Data Processing Law (2014). Data State Inspectorate. Privacy/Data Protection |  |  |  | X |  |
| Ethics Committee Guidelines: Procedures for Researchers, Section 1 (2011). Ministry of Health and Social Welfare. General |  |  |  | X |  |
| Operational Guidelines of the National Research Ethics Board (2019). Ministry of Health and Social Welfare. General |  |  |  | X |  |
| Act of 1 August 2018 on the Organisation of the National Data Protection Commission, Articles 63-65. National Data Protection Commission. Privacy/Data Protection |  |  |  | X |  |
| Circular on Human Biological Samples and Participants Recompense in Research Involving Human Subjects (2019). National Commission for Science and Technology. Human Biological Materials |  |  |  | X |  |
| A Guide To Conducting Clinical Trials in Malaysia (2016). Ministry of Health Malaysia, National Pharmaceutical Regulatory Agency (NPRA), National Committee for Clinical Research (NCRC), Clinical Research Malaysia (CRM), Ministry of Health e Society of Clinical Research Professionals Malaysia (SCRPM). Drugs, Biologics, and Devices |  |  |  | X |  |
| Act 709: Personal Data Protection Act (2010): Section 38, 39 and 40. Department of Personal Data Protection. Privacy/Data Protection |  |  |  | X |  |
| ASM, The Malaysian Code of Responsible Conduct in Research (2020). Academy of Sciences Malaysia (ASM). General | X |  |  |  | Not relevant |
| Guidelines on Ethical Issues in the provision of Medical Genetics Services in Malaysia (2019). Malaysian Medical Council, Laws of Malaysia. Attorney General’s Chambers of Malaysia (AGC), Medical Development Division, Ministry of Health (MOH) e Ministry of Energy and Natural Resources. Genetic Research | X |  |  |  | Not relevant |
| Malaysian Guideline for Application of Clinical Trial Import License and Clinical Trial Exemption, 7th Edition (2021). Ministry of Health Malaysia, National Institutes of Health, Medical Review and Ethics Committee (MREC), Malaysian Industry-Government Group For High Technology (MIGHT) e Academy of Sciences Malaysia (ASM). General |  |  |  | X |  |
| Clinical Practice Guidelines for Managing Frailty in Community-Dwelling Korean Elderly Adults in Primary Care Settings | X |  |  |  | Not relevant |
| Malaysian Guideline for Phase I Unit Inspection and Accreditation Program (2018). Ministry of Health Malaysia, National Pharmaceutical Regulatory Agency (NPRA), National Committee for Clinical Research (NCRC), Clinical Research Malaysia (CRM), Ministry of Health e Society of Clinical Research Professionals Malaysia (SCRPM). Drugs, Biologics, and Devices |  |  |  | X |  |
| Malaysian Guidelines of Good Clinical Practice (2020). Ministry of Health Malaysia, National Institutes of Health, Medical Review and Ethics Committee (MREC), Malaysian Industry-Government Group For High Technology (MIGHT) e Academy of Sciences Malaysia (ASM). General |  |  |  | X |  |
| NMRR, Guidelines, various. National Medical Research Register (NMRR). Clinical Trial Registries |  |  |  | X |  |
| Occupational Safety and Health Act 1994: Section 32. Ministry of Health Malaysia, National Pharmaceutical Regulatory Agency (NPRA), Attorney General’s Chambers of Malaysia (AGC), Department of Occupational Safety and Health (DOSH), Ministry of Human Resources e National Committee for Clinical Research (CRC). Research Injury |  |  |  | X |  |
| The Malaysian Code of Responsible Conduct in Research (2020). Malaysian Industry-Government Group For High Technology (MIGHT) e Ministry of Health Malaysia, Institute for Health Behavioural Research (IPTK). Social-Behavioral Research |  |  |  | X |  |
| Data Protection Act, 2002. Office of the Information and Data Protection Commissioner. Privacy/Data Protection |  |  |  | X |  |
| Guidance Notes on Good Clinical Practice (2018). Medicines Authority. Drugs, Biologics, and Devices |  |  |  | X |  |
| Medicines Act, 2003. Medicines Authority. Drugs, Biologics, and Devices |  |  |  | X |  |
| Product Safety Act, 2001. Medicines Authority, Malta Competition and Consumer Affairs Authority, Technical Regulations Division. Drugs, Biologics, and Devices |  |  |  | X |  |
| Subsidiary Legislation, 427.44, Medical Devices Regulations, 2010. Medicines Authority, Malta Competition and Consumer Affairs Authority, Technical Regulations Division. Drugs, Biologics, and Devices |  |  |  | X |  |
| Subsidiary Legislation, 458.43, Clinical Trials Regulations, 2004. Medicines Authority. Drugs, Biologics, and Devices |  |  |  | X |  |
| Subsidiary Legislation, 458.47, Good Clinical Practice and Requirements for Manufacturing or Import Authorisation of Investigational Medicinal Products (Human Use) Regulations, 2004. Medicines Authority. Drugs, Biologics, and Devices |  |  |  | X |  |
| CCMO Memorandum, Definition of Medical Research. Ministry of Health, Welfare, and Sport (VWS), Central Committee for Research Involving Human Subjects (CCMO) e Medicines Evaluation Board (MEB). Drugs, Biologics, and Devices |  |  |  | X |  |
| CCMO, Decree of 2014 containing rules for compulsory insurance in medical research involving human subjects and explanatory memorandum:. Ministry of Health, Welfare and Sport. Research Injury |  |  |  | X |  |
| CCMO, Memorandum Behavioural Research. National Ethics Council for Social and Behavioural Sciences. Social-Behavioral Research |  |  |  | X |  |
| Ethical Code (2018). National Ethics Council for Social and Behavioural Sciences. Social-Behavioral Research | X |  |  |  | Not relevant |
| Human Tissue and Medical Research: Code of Conduct for responsible use (2011). Central Committee for Research Involving Human Subjects (CCMO). Human Biological Materials | X |  |  |  | Not relevant |
| Council of Europe Committee on Bioethics Guide for research ethics committee members. Council of Europe, Bioethics Unit. Research Injury | X |  |  |  | Not relevant |
| Accident Compensation Act 2001. Health Research Council (HRC) Ethics Committee, National Ethics Advisory Committee (NEAC), Ministry of Health (MOH), Health and Disability Commissioner (HDC), Health and Disability Ethics Committees e Ministry of Business, Innovation and Employment. General |  |  |  | X |  |
| Conducting Medical Device Clinical Trials in New Zealand, various. New Zealand Medicines and Medical Devices Safety Authority (Medsafe). Drugs, Biologics, and Devices |  |  |  | X |  |
| HDC, The Code of Health and Disability Services Consumers’ Rights (the Code of Rights) (2004). Health and Disability Commissioner (HDC). General |  |  |  | X |  |
| Health Act 1956 (2012). Ministry of Health (MOH), Health Research Council (HRC) Ethics Committee, Te Puni Kokiri (TPK), Office of the Health and Disability Commissioner (HDC) e Ministry of Business, Innovation and Employment. Human Biological Materials | X |  | X |  | Not relevant |
| Health and Disability Commissioner Act 1994. Health Research Council (HRC) Ethics Committee, National Ethics Advisory Committee (NEAC), Ministry of Health (MOH), Health and Disability Commissioner (HDC), Health and Disability Ethics Committees e Ministry of Business, Innovation and Employment. General |  |  |  | X |  |
| Health Information Privacy Code 1994. Privacy Commissioner. Privacy/Data Protection |  |  |  | X |  |
| Health Research Council Act 1990, Sections 24 and 25. Health Research Council (HRC) Ethics Committee, National Ethics Advisory Committee (NEAC), Ministry of Health (MOH), Health and Disability Commissioner (HDC), Health and Disability Ethics Committees e Ministry of Business, Innovation and Employment. General |  |  |  | X |  |
| HRC, The Role of Ethics, various. Health Research Council (HRC) Ethics Committee. General |  |  |  | X |  |
| Medicines (Database of Medical Devices) Regulations (2003). New Zealand Medicines and Medical Devices Safety Authority (Medsafe). Drugs, Biologics, and Devices |  |  |  | X |  |
| Medicines Act 1981 (2012). New Zealand Medicines and Medical Devices Safety Authority (Medsafe), Medicines New Zealand e Health Research Council (HRC), Standing Committee on Therapeutic Trials. Drugs, Biologics, and Devices |  |  |  | X |  |
| Medsafe, Good Clinical Research Practice and Obtaining Approval for Clinical Trials (2013). New Zealand Medicines and Medical Devices Safety Authority (Medsafe). Drugs, Biologics, and Devices |  |  |  | X |  |
| MOH, Standard Operating Procedures for Health and Disability Ethics Committees (2012). Ministry of Health (MOH). General | X |  |  |  | Not relevant |
| New Zealand Bill of Rights Act. Health Research Council (HRC) Ethics Committee, National Ethics Advisory Committee (NEAC), Ministry of Health (MOH), Health and Disability Commissioner (HDC), Health and Disability Ethics Committees e Ministry of Business, Innovation and Employment. General |  |  |  | X |  |
| New Zealand Public Health and Disability Act 2000, Section 16. Health Research Council (HRC) Ethics Committee, National Ethics Advisory Committee (NEAC), Ministry of Health (MOH), Health and Disability Commissioner (HDC), Health and Disability Ethics Committees e Ministry of Business, Innovation and Employment. General |  |  |  | X |  |
| Privacy Act 1993 (2012). Public Records Act (2005). Privacy Commissioner. Privacy/Data Protection |  |  |  | X |  |
| National Health Act (2014). National Health Research Ethics Committee. General | X |  |  |  | Not relevant |
| Guidelines for Research Ethics in Science and Technology (2016). Norwegian Directorate of Health e Norwegian Biotechnology Advisory Board e National Committee for Medical and Health Research Ethics (NEM), Regional Committees for Medical and Health Research Ethics (REK) e National Committee for Research Ethics in Science and Technology (NENT). Genetic Research |  |  |  | X |  |
| Guidelines for Research Ethics on Human Remains. National Committee for Medical and Health Research Ethics (NEM), Regional Committees for Medical and Health Research Ethics (REK), National Committee for Research Ethics in Science and Technology (NENT) e National Committee for Research Ethics on Human Remains. General |  |  |  | X |  |
| Law regarding Ethics and Integrity in Research (2006). National Committee for Medical and Health Research Ethics (NEM), Regional Committees for Medical and Health Research Ethics (REK), National Committee for Research Ethics in Science and Technology (NENT) e National Committee for Research Ethics on Human Remains. General |  |  |  | X |  |
| Data Privacy Act Implementing Rules and Regulations (2016). Privacy/Data Protection | X |  |  |  | Not relevant |
| FDA, Guidelines: Regulation of Clinical Trials in the Philippines. Food and Drug Administration (FDA). Drugs, Biologics, and Devices | X |  |  |  | Not relevant |
| Memorandum: Registration and Accreditation of all Ethics Review Committees in the Philippines (2015). Philippine Health Research Ethics Board (PHREB), Department of Science and Technology (DOST), Department of Health (DOH), Commission of Higher Education (CHED) e National Commission on Indigenous Peoples (NCIP). General |  |  |  | X |  |
| PNHRS Act Implementing Rules and Regulations. Philippine Health Research Ethics Board (PHREB), Department of Science and Technology (DOST), Department of Health (DOH), Commission of Higher Education (CHED) e National Commission on Indigenous Peoples (NCIP). General |  |  |  | X |  |
| Republic Act No. 10173: Data Privacy Act of 2012. Privacy/Data Protection | X |  |  |  | Not relevant |
| Republic Act No. 10532: An Act Institutionalizing the Philippine National Health Research System (2013). Philippine Health Research Ethics Board (PHREB), Department of Science and Technology (DOST), Department of Health (DOH), Commission of Higher Education (CHED) e National Commission on Indigenous Peoples (NCIP). General |  |  |  | X |  |
| Human Research Policies & Regulations, various. Ministry of Public Health, Health Research Governance Department. General |  |  |  | X |  |
| Clinical Trials Act (2016). Drugs, Biologics, and Devices | X |  |  |  | Not relevant |
| Rulebook on the Contents of the Application, and/or Documentation on the Approval of Clinical Trials for Medicines and Medical Devices, as well as the Method of Implementation for Clinical Trials of Medicines and Medical Devices, Official Gazette of RS, 64/2011, 91/2013, 60/2016, and 9/2018. Ministry of Health (MOH) e Medicines and Medical Devises Agency of Serbia. General | X |  |  |  | Not relevant |
| Search approved clinical trials. Medicines and Medical Devises Agency of Serbia. Clinical Trial Registries |  |  |  | X |  |
| Guidelines, Clinicial Trials and Forms various. Ministry of Health e Pharmacy Board of Sierra Leone. Drugs, Biologics, and Devices |  |  |  | X |  |
| Guide on the Requirement of Appropriate Consent for the Conduct of HBR and Handling of Human Tissue (2019). Ministry of Health (MOH) e Bioethics Advisory Committee (BAC). Human Biological Materials | X |  |  |  | Not relevant |
| Health Products (Clinical Trials) Regulations 2016. Health Sciences Authority of Singapore (HSA), Ministry of Health (MOH), National Environment Agency (NEA), Centre For Radiation Protection And Nuclear Science. Drugs, Biologics, and Devices |  |  |  | X |  |
| Health Products (Therapeutic Products as Clinical Research Materials) Regulations 2016. Health Sciences Authority of Singapore (HSA), Ministry of Health (MOH), National Environment Agency (NEA), Centre For Radiation Protection And Nuclear Science. Drugs, Biologics, and Devices |  |  |  | X |  |
| Health Products Act 2007. Health Sciences Authority of Singapore (HSA), Ministry of Health (MOH), National Environment Agency (NEA), Centre For Radiation Protection And Nuclear Science. Drugs, Biologics, and Devices |  |  |  | X |  |
| Healthcare Sector Specific Guidelines Promulgated by PDPC. Personal Data Protection Commission (PDPC). Privacy/Data Protection |  |  |  | X |  |
| Human Biomedical Research Act 2015. Ministry of Health (MOH) e Bioethics Advisory Committee (BAC). General | X |  |  |  | Not relevant |
| Medical (Therapy, Education, and Research) Act 1972. Ministry of Health (MOH) e Bioethics Advisory Committee (BAC). Human Biological Materials |  |  |  | X |  |
| Medicines (Clinical Trials) Regulations (2016). Ministry of Health (MOH) e Health Sciences Authority. Research Injury |  |  |  | X |  |
| Medicines (Medicinal Products as Clinical Research Materials) Regulations 2016. Health Sciences Authority of Singapore (HSA), Ministry of Health (MOH), National Environment Agency (NEA), Centre For Radiation Protection And Nuclear Science. Drugs, Biologics, and Devices |  |  |  | X |  |
| Medicines Act 1975. Health Sciences Authority of Singapore (HSA), Ministry of Health (MOH), National Environment Agency (NEA), Centre For Radiation Protection And Nuclear Science. Drugs, Biologics, and Devices |  |  |  | X |  |
| Personal Data Protection Act 2012. Ministry of Health (MOH), Personal Data Protection Commission (PDPC) e Bioethics Advisory Committee (BAC). Privacy/Data Protection |  |  |  | X |  |
| Resources on Human Biomedical Research Act. Ministry of Health (MOH) e Bioethics Advisory Committee (BAC). General |  |  |  | X |  |
| Singapore Guidance on Good Clinical Practice Compliance Inspection Framework (2021). Health Sciences Authority of Singapore (HSA), Ministry of Health (MOH), National Environment Agency (NEA), Centre For Radiation Protection And Nuclear Science. Drugs, Biologics, and Devices |  |  |  | X |  |
| Singapore Guideline for Good Clinical Practice (2016). Ministry of Health (MOH) e Health Sciences Authority. Research Injury |  |  |  | X |  |
| Act no. 18/2018 On Personal Data Protection and Amending and Supplementing Certain Acts (2018). Office for Personal Data Protection. Privacy/Data Protection |  |  |  | X |  |
| Act No. 428/2002 Coll. on Protection of Personal Data, as amended by Act No. 90/2005 Coll. Office for Personal Data Protection. Privacy/Data Protection |  |  |  | X |  |
| Oviedo Convention on Human Rights and Biomedicine. Council of Europe, Bioethics Unit. |  |  |  | X |  |
| Additional Protocol on Biomedical Research (2005). General | X |  |  |  | Not relevant |
| Additional Protocol to the Convention for the Protection of Human Rights and Dignity of the Human Being with regard to the Application of Biology and Medicine, on the Prohibition of Cloning Human Beings (1998). Human Biological Materials |  |  |  | X |  |
| Additional Protocol to the Convention on Human Rights and Biomedicine Concerning Transplantation of Organs and Tissues of Human Origin (2002). Human Biological Materials |  |  |  | X |  |
| Additional Protocol to the Convention on Human Rights and Biomedicine concerning Genetic Testing for Health Purposes (2008). Human Biological Materials |  |  |  | X |  |
| National Health Act No. 61, Chapter 9 (2003). Department of Health (DH), Medical Research Council of South Africa (MRC), Human Sciences Research Council (HSRC) e South African Health Products Regulatory Authority. General | X |  |  |  | Not relevant |
| Bioethics and Safety Act No. 16372 (2019.04.23). Ministry of Health and Welfare (MOHW). General | X |  |  |  | Not relevant |
| Enforcement Decree of Bioethics and Safety Act No. 30141 (2019.10.22). Ministry of Health and Welfare (MOHW). General | X |  |  |  | Not relevant |
| Personal Information Protection Act No.16930 (2020.02.). Ministry of Health and Welfare e Ministry of the Interior and Safety. Social-Behavioral Research |  |  |  | X |  |
| Accreditation Guidelines for Research Ethics Committees in Sudan (2017). Federal Ministry of Health. General |  |  |  | X |  |
| National Guidelines for Ethical Conduct of Research Involving Human Subjects (2008). Federal Ministry of Health. General |  |  |  | X |  |
| Good Research Practice (2017). Swedish Ethical Review Authority, Ethics Review Appeal Board e Swedish Research Council. General |  |  |  | X |  |
| Good Research Practice: Observational Studies Conducted Through Participating, Observing, and Recording (2017). Swedish Research Council. Social-Behavioral Research |  |  |  | X |  |
| Federal Act of 19 June 1992 on Data Protection (FADP), RS 235.1. Federal Data Protection and Information Commissioner (FDPIC). Privacy/Data Protection |  |  |  | X |  |
| Swiss Clinical Trial Organisation, Guidelines for Good Operational Practice (GGOP) (2017). Federal Office of Public Health (FOPH), Federal Office of Public Health, Portal for Human Research (FOPH), National Advisory Commission on Biomedical Ethics (NEK-CNE) e Swiss Association of Research Ethics Committees. General |  |  |  | X |  |
| ESRC Research Ethics Framework (REF). Research ethics and social sciences | X |  |  |  | Not relevant |
| ESRC, Framework for Research Ethics (2015). Economic and Social Research Council. Social-Behavioral Research | X |  |  |  | Not relevant |
| Enforcement Rules of the Personal Data Protection Act (2016). Ministry of Justice. Privacy/Data Protection |  |  |  | X |  |
| FDA, Regulation for Good Clinical Practice (2014). Food and Drug Administration (FDA). Research Injury | X |  |  |  | Not relevant |
| Medical Care Act (2018). Ministry of Health and Welfare (MOHW). General |  |  |  | X |  |
| Regulations for Good Clinical Practice (2014). Ministry of Health and Welfare (MOHW) e Taiwan Food and Drug Administration (FDA). Drugs, Biologics, and Devices | X |  |  |  | Not relevant |
| Regulations Governing the Organization and Operation of the Human Research Ethics Review Board (2018). Ministry of Health and Welfare (MOHW). General |  |  |  | X |  |
| Regulations on Human Trials (2009). Ministry of Health and Welfare. Human Biological Materials | X |  |  |  | Not relevant |
| Regulations on Human Trials (2016). Ministry of Health and Welfare (MOHW). General |  |  |  | X |  |
| National Institute for Medical Research, Act of Parliament No. 23, of 1979. Ministry of Health (MOH), National Institute for Medical Research (NIMR), National Health Research Ethics Committee (NHREC) e Tanzania Commission for Science and Technology (COSTECH). General |  |  |  | X |  |
| NIMR, Research Policies, Guidelines, and Regulations. National Institute for Medical Research (NIMR). General | X |  |  |  | Not relevant |
| Medical Professions Act (2009), Articles 47-50. National Research Council of Thailand (NCRT), Medical Council of Thailand (MCT) e Forum for Ethical Review Committees in Thailand (FERCIT). General |  |  |  | X |  |
| Ministerial Regulations, various. Office of the Information Commission. Privacy/Data Protection |  |  |  | X |  |
| NCRT, Guidance for Foreign researcher Conducting Research in Thailand. National Research Council of Thailand (NCRT). General |  |  |  | X |  |
| GCP Guideline (2015). Turkey Pharmaceuticals and Medical Devices Agency (Turkish) (TITCK), Clinical Research Association (CRA)e Ministry of Health (MoH). Drugs, Biologics, and Devices |  |  |  | X |  |
| Personal Data Protection Law. Personal Data Protection Authority. Privacy/Data Protection |  |  |  | X |  |
| Human Medicine Guidelines, including Guidelines for the Conduct of Drug Related Clinical Trials (2019). National Drug Authority. Drugs, Biologics, and Devices |  |  |  | X |  |
| National Drug Policy and Authority Act Regulations. National Drug Authority. Drugs, Biologics, and Devices |  |  |  | X |  |
| National Guidelines for the Conduct of Research During the COVID-19 Pandemic. Uganda National Council for Science and Technology (UNCST). General | X |  |  |  | Not relevant |
| Research Registration and Clearance Policy and Guidelines (2016). Uganda National Council for Science and Technology (UNCST). General |  |  |  | X |  |
| Convention for the Protection of Individuals with Regard to Automatic Processing of Personal Data (2010). State Service of Ukraine on Personal Data Protection e Ukrainian Parliament Commissioner for Human Rights. Privacy/Data Protection |  |  |  | X |  |
| Health Care Law, Article 45 (1992). Ukrainian Ministry of Health. General | X |  |  |  | Not relevant |
| Healthcare Guidelines. Health Authority - Abu Dhabi. General | X |  |  |  | Not relevant |
| ABHI, Clinical Investigations Compensation Guidelines (2014). Association of the British Healthcare Industry (ABHI). Research Injury |  |  |  | X |  |
| ABPI, Clinical Trial Compensation Guidelines (2014). Association of the British Pharmaceutical Industry (ABPI). Research Injury |  |  |  | X |  |
| ABPI, Insurance and Compensation in the Event of Injury in Phase I Clinical Trials (2012). Association of the British Pharmaceutical Industry (ABPI). Research Injury |  |  |  | X |  |
| Good Practice in Research: Internet-Mediated Research (2016). Economic and Social Research Council e UK Research Integrity Office. Social-Behavioral Research |  |  |  | X |  |
| HTA, Guidance for Professionals. Human Tissue Authority (HTA). Human Biological Materials |  |  |  | X |  |
| Statutory Instrument 2006 No. 1659: The Human Tissue Act 2004 (Persons who Lack Capacity to Consent and Transplants) Regulations (2006) (Different provisions apply to England, Wales, Northern Ireland, and/or Scotland). Human Tissue Authority (HTA) e Medical Research Council (MRC). Human Biological Materials |  |  |  | X |  |
| Amendment Regulations (SI 2006/1928). Medicines and Healthcare Products Regulatory Agency (MHRA), Administration of Radioactive Substances Advisory Committee (ARSAC) (UK), Department of Environment, Food & Rural affairs (DEFRA), Health and Safety Executive (HSE), Association of the British Pharmaceutical Industry (ABPI), National Institute for Health Research e Health Research Authority (HRA). Drugs, Biologics, and Devices |  |  |  | X |  |
| Amendment to the Medicines for Human Use (Clinical Trials) Regulations 2004 and Adults with Incapacity (Scotland) Act 2000 to Facilitate Clinical Research in Emergency Settings (SI 2006/2984). Medicines and Healthcare Products Regulatory Agency (MHRA), Administration of Radioactive Substances Advisory Committee (ARSAC) (UK), Department of Environment, Food & Rural affairs (DEFRA), Health and Safety Executive (HSE), Association of the British Pharmaceutical Industry (ABPI), National Institute for Health Research e Health Research Authority (HRA). Drugs, Biologics, and Devices |  |  |  | X |  |
| Clinical Trials for Medical Devices. Medicines and Healthcare Products Regulatory Agency (MHRA) e Health Research Authority (HRA). Drugs, Biologics, and Devices |  |  |  | X |  |
| Data Protection Act (2018). Information Commissioner’s Office, Health Research Authority (HRA) e Medical Research Council (MRC). Privacy/Data Protection |  |  |  | X |  |
| HRA, Clinical Trials of Investigational Medicinal Products (CTIMPs). National Institute for Health Research e Health Research Authority (HRA). Drugs, Biologics, and Devices |  |  |  | X |  |
| HRA, Consent in Research (2018). Health Research Authority (HRA). Privacy/Data Protection |  |  |  | X |  |
| HRA, GDPR Guidance. Health Research Authority (HRA). Privacy/Data Protection |  |  |  | X |  |
| HRA, Transparency: Researchers’ Responsibilities. Health Research Authority (HRA). Clinical Trial Registries |  |  |  | X |  |
| Medicines for Human Use (Clinical Trials) Regulations, Statutory Instrument No. 1031 (2004). Medicines and Healthcare Products Regulatory Agency (MHRA), Administration of Radioactive Substances Advisory Committee (ARSAC) (UK), Department of Environment, Food & Rural affairs (DEFRA), Health and Safety Executive (HSE), Association of the British Pharmaceutical Industry (ABPI), National Institute for Health Research e Health Research Authority (HRA). Drugs, Biologics, and Devices |  |  |  | X |  |
| MRC, Using Information About People in Health Research (2017). Medical Research Council (MRC). Privacy/Data Protection | X |  |  |  | Not relevant |
| National Institute for Health Research, Clinical Trials Toolkit. Medicines and Healthcare Products Regulatory Agency (MHRA), Administration of Radioactive Substances Advisory Committee (ARSAC) (UK), Department of Environment, Food & Rural affairs (DEFRA), Health and Safety Executive (HSE), Association of the British Pharmaceutical Industry (ABPI), National Institute for Health Research e Health Research Authority (HRA). Drugs, Biologics, and Devices |  |  |  | X |  |
| Notify MHRA About a Clinical Investigation for a Medical Device. Medicines and Healthcare Products Regulatory Agency (MHRA). Drugs, Biologics, and Devices |  |  |  | X |  |
| SI 2008 No.941 The Medicines for Human Use (Clinical Trials) and Blood Safety and Quality Amendment Regulations 2008. Medicines and Healthcare Products Regulatory Agency (MHRA), Administration of Radioactive Substances Advisory Committee (ARSAC) (UK), Department of Environment, Food & Rural affairs (DEFRA), Health and Safety Executive (HSE), Association of the British Pharmaceutical Industry (ABPI), National Institute for Health Research e Health Research Authority (HRA). Drugs, Biologics, and Devices |  |  |  | X |  |
| DHSC, Care Act (2014). Department of Health and Social Care (DHSC). General |  |  |  | X |  |
| DHSC, Health and Social Care Act (2012). Department of Health and Social Care (DHSC). General |  |  |  | X |  |
| DHSC, Mental Capacity Act (2005). Department of Health and Social Care (DHSC). General |  |  |  | X |  |
| HRA, Governance Arrangements for Research Ethics Committees (2018). Health Research Authority (HRA). General |  |  |  | X |  |
| HRA, Guidance. Health Research Authority (HRA). General |  |  |  | X |  |
| HRA, Integrated Research Application System. Health Research Authority (HRA). General |  |  |  | X |  |
| HRA, Research Governance Framework for Health and Social Care UK Policy Framework for Health and Social Care Research (2018). Health Research Authority (HRA). General |  |  |  | X |  |
| MRC, Good Research Practice: Principles and Guidelines (2012). Medical Research Council (MRC). General |  |  |  | X |  |
| MRC, Medical Research Involving Adults Who Cannot Consent (2007). Medical Research Council (MRC). General | X |  |  |  | Not relevant |
| MRC, Research Involving Human Participants in Developing Societies (2004). Medical Research Council (MRC). General |  |  |  | X |  |
| Health Service (Control of Patient Information) Regulations 2002 (HS (CPI) Regs). Health Research Authority (HRA) (England) e Confidentiality Advisory Group (CAG). Privacy/Data Protection |  |  |  | X |  |
| HRA, Research Data and Tissue Resources. Health Research Authority (HRA). Privacy/Data Protection |  |  |  | X |  |
| Adults with Incapacity (Ethics Committee) Amendment Regulations (2002). NHSScotland, Chief Scientist Office (CSO) e NHS Research Scotland. General |  |  |  | X |  |
| Adults with Incapacity Act 2000, Section 51. NHSScotland, Chief Scientist Office (CSO) e NHS Research Scotland. General |  |  |  | X |  |
| CSO, Research Governance Framework for Health and Community Care (2006). NHSScotland, Chief Scientist Office (CSO). General | X |  |  |  | Not relevant |
| Research Governance Framework for Health and Social Care in Wales Second Edition (2009). Health and Care Research Wales. General | X |  |  |  | Not relevant |
| Agency for Healthcare Research and Quality (AHRQ), Confidentiality in AHRQ-Supported Research (2018). Privacy/Data Protection |  |  |  | X |  |
| Confidential Information Protection and Statistical Efficiency Act (CIPSEA) (2002). Privacy/Data Protection |  |  |  | X |  |
| OHRP, Human Research Protections Guidance, various - (2018). Department of Health and Human Services (HHS), Office for Civil Rights (OCR), Health Insurance Portability and Accountability Act (HIPAA)(1996). General |  |  |  | X |  |
| Department of Health and Human Services (HHS), Office for Human Research Protections (OHRP). - 45 CFR 46, Subparts A (the Common Rule), B, C, D, and E; |  |  |  | X |  |
| Department of Justice, Privacy Act, 5 U.S.C. § 552a (1974). Privacy/Data Protection |  |  |  | X |  |
| E-Government Act of 2002, Public Law 107-347. Privacy/Data Protection | X |  |  |  | Not relevant |
| Guidance, various. Department of Health and Human Services, Office for Human Research Protections (OHRP). Human Biological Materials |  |  |  | X |  |
| Health Information Technology for Economic and Clinical Health (HITECH) Act (2009). Privacy/Data Protection | X |  |  |  | Not relevant |
| HHS, OCR, 21st Century Cures Act Research Guidance on Activities Preparatory to Research (2017). Privacy/Data Protection |  |  |  | X |  |
| HHS, OCR, 21st Century Cures Act Research Guidance on Streamlining Authorization (2018). Privacy/Data Protection |  |  |  | X |  |
| HIPAA Breach Notification Rule, 45 CFR §164.400-414. Privacy/Data Protection |  |  |  | X |  |
| HIPAA Privacy Rule, 45 CFR parts 160 and 164, Subparts A and C (2002). Privacy/Data Protection |  |  |  | X |  |
| HIPAA Security Rule, 45 CFR parts 160, 162, and 164 (2009). Privacy/Data Protection |  |  |  | X |  |
| NIH Policy on Certificates of Confidentiality (2017). Privacy/Data Protection |  |  |  | X |  |
| Subpart A of the HHS regulations for the protection of research participants at 45 CFR 46 is often referred to as the Common Rule because various Federal departments and agencies have adopted the same regulations. General | X |  |  |  | Not relevant |
| 21st Century Cures Act, Section 3024 (2016). Food and Drug Administration. Drugs, Biologics, and Devices |  |  |  | X |  |
| Department of Health and Human Services, Sections 116(a)(6) and (7) of the Common Rule. Research Injury |  |  |  | X |  |
| Department of Veterans Affairs, 38 CFR 17.85: Treatment of Research-Related Injuries to Human Subjects. Research Injury |  |  |  | X |  |
| Department of Veterans Affairs, Handbook 1200.5, Appendix F, Paragraph 2a(11). Research Injury |  |  |  | X |  |
| FDA, Good Clinical Practice and Human Subject Protection in FDA-Regulated Clinical Trials. Food and Drug Administration. Drugs, Biologics, and Devices |  |  |  | X |  |
| FDA, Regulations, Good Clinical Practice and Clinical Trials, various. Food and Drug Administration. Drugs, Biologics, and Devices |  |  |  | X |  |
| Clinical Trials Regulation and Results Information Submission, 42 CFR 11 (2016). Food and Drug Administration, National Institutes of Health ClinicalTrials e Office of Research Oversight (ORO). Clinical Trial Registries |  |  |  | X |  |
| NIH Policy on the Dissemination of NIH-Funded Clinical Trial Information (2016). National Institutes of Health ClinicalTrials. Clinical Trial Registries | X |  |  |  | Not relevant |
| OHRP, Clinical Trial Informed Consent Form Posting (45 CFR 46.116(h)). Food and Drug Administration, National Institutes of Health ClinicalTrials e Office of Research Oversight (ORO). Clinical Trial Registries |  |  |  | X |  |
| Circular – Guidelines for Clinical Trials on Drugs (C-ClinDrugTrial), Articles 2, 4, 5, 9, 17, 18, 31, and 39 (2012). Ministry of Health. Drugs, Biologics, and Devices |  |  |  | X |  |
| Decision No. 111/QD-BYT – On Promulgation of Regulation on Organization and Operation of Council of Ethics in Biomedical Research at Grass-Roots Level, Chapter I (Articles 3 and 4), Chapter II, and Chapter III (2013). Ministry of Health (MOH). General | X |  |  |  | Not relevant |
| Decision No. 460/QD-BYT – On the Promulgation of Regulations on Organization and Operation of Ethical Evaluation Committee in Biomedical Research of the Ministry of Health, Period 2012-2017, Chapters I-III (2012). Ministry of Health (MOH). General |  |  |  | X |  |
| Decision No. 799/QD-BYT of the Minister of Health on the Promulgation of the Guidelines on Good Clinical Practice of Clinical Trials (2008). Ministry of Health. Drugs, Biologics, and Devices | X |  |  |  | Not relevant |
| Guidelines for Clinical Trials of Drugs, Chapter III, Articles 10, 16, and 17 (2012). Ministry of Health. Drugs, Biologics, and Devices |  |  |  | X |  |
| National Health Research Act (2013). Ministry of Health. General |  |  |  | X |  |
| Access to Information and Protection of Privacy Act, Chapter 10:27. Registrar General e Zimbabwe National Statistics Agency. Privacy/Data Protection |  |  |  | X |  |
| Research Act (1986). Medical Research Council of Zimbabwe. General |  |  |  | X |  |
| Research Act (2001). Research Council of Zimbabwe. Human Biological Materials |  |  |  | X |  |
| Ethical Practitioners' Association of Canada (EPAC ), Ethical Standards. Research ethics and social sciences |  |  |  | X |  |
| University of Toronto Social Sciences and Humanities Research Ethics Board (SSH REB) Guidelines for Ethical Conduct in Participant Observation. Research ethics and social sciences |  |  |  | X |  |
| Article 6 of Decision No 1982/2006/EC of the European Parliament and of the Council of 18 December 2006 concerning the Seventh Framework Programme of the European Community for research, technological development and demonstration activities (2007-2013). Research Ethics and the European Commission's Ethics Review | X |  |  |  | Not relevant |
| European Convention for the Protection of Human Rights and Fundamental Freedoms (ECHR). Research involving data processing |  |  |  | X |  |
| Charter of Fundamental Rights (2000). Research involving medical intervention |  |  |  | X |  |
| Commission communication on the Charter of fundamental rights of the European Union (2000). Research involving medical intervention |  |  |  | X |  |
| Communication from the Commission on the legal nature of the Charter of fundamental rights of the European Union (2000). Research involving medical intervention |  |  |  | X |  |
| Working Party on the Protection of Individuals with regard to the Processing of Personal data (Article 29 Working Group) - Working Document on the processing of personal data relating to health in electronic health records (EHR), 15 February 2007, WP 131. Research involving data processing | X |  |  |  | Not relevant |
| Council of Europe European Social Charter Strasbourg (1996). Research involving medical intervention |  |  |  | X |  |
| Directive 2000/70/EC of the European Parliament and of the Council of 16 November 2000 amending Council Directive 93/42/ EEC as regards medical devices incorporating stable derivates of human blood or human plasma. Research involving medical intervention |  |  |  | X |  |
| Directive 2001/83/EC on the Community code relating to medicinal products for human use (the Community Code for medicinal products). Research involving medical intervention |  |  |  | X |  |
| Directive 2003/63/EC of 25 June 2003 amending Directive 2001/83/EC of the European Parliament and of the Council on the Community code relating to medicinal products for human use. Research involving medical intervention | X |  | X |  | Not relevant |
| Directive 90/385/EEC of 20 June 1990 on the approximation of the laws of the Member States relating to active implantable medical devices. Research involving medical intervention |  |  |  | X |  |
| Directive 95/46/EC of the European Parliament and of the Council of 24 October 1995 on the protection of individuals with regard to the processing of personal data and on the free movement of such data. Research involving data processing |  |  |  | X |  |
| ESOMAR - World Association of Opinion & Marketing Research Professionals (European Society for Opinion & Marketing Research). International Code of Marketing & Social Research Practices. Research ethics and social sciences | X |  |  |  | Not relevant |
| European Commission - DG ENTERPRISE - Detailed guidance on the collection, verification and presentation of adverse reaction reports arising from clinical trials on medicinal products for human use (revision 2) as required by Article 18 of Directive 2001/20/EC , 2006. Research involving medical intervention |  |  |  | X |  |
| European Commission - DG ENTERPRISE - Detailed guidance on the European clinical trials database (EUDRACT Database) as required by Article 11 and Article 17 of Directive 2001/20/EC , CT 5.1 Amendment describing the development of EudraCT Lot 1 for 1 May 2004 and CT 5.2 EudraCT core dataset, 2003. Research involving medical intervention |  |  |  | X |  |
| European Group on Ethics in Science and New Technologies (EGE) - Opinion n°20 -16/03/2005 - Ethical aspects of ICT Implants in the Human Body. Research involving medical intervention | X |  |  |  | Not relevant |
| EuroSOCAP-Project: European Guidance for Healthcare Professional on Confidentiality and Privacy in Healthcare (2006). Research involving medical intervention |  |  |  | X |  |
| Recommendation (2004) 10 of the Committee of Ministers to member states concerning the protection of the human rights and dignity of persons with mental disorder. Research involving medical intervention |  |  |  | X |  |
| Recommendation (90) 3 of the Committee of Ministers to member states concerning medical research on human beings. Research involving medical intervention |  |  |  | X |  |
| Recommendations, Resolutions and Opinions of the Parliamentary Assembly, resolutions from ministerial conferences, and principles set out in the report of the Ad hoc Committee of Experts on Progress in the Biomedical Sciences (CAHBI). Research involving medical intervention |  |  |  | X |  |
| Regulation (EC) 45/2001 of the European Parliament and of the Council of 18. December 2000 on the protection of individuals with regard to the processing of personal data by the Community institutions and bodies and on the free movement of such data. Research involving data processing |  |  |  | X |  |
| Regulation (EC) No 1901/2006 of the European Parliament and the Council, as amended, on medicinal products for paediatric use (herein the 'Paediatric Regulation'). Research involving children |  |  |  | X |  |
| UN Cartagena Protocol on Biosafety to the Convention on Biological Diversity. Research involving genetic modification |  |  |  | X |  |
| CHMP Guideline on conduct of Pharmacovigilance for medicines used by the paediatric population (June 2006) EMEA/CHMP/PhVWP/235910/2005- rev.1. Research involving children | X |  |  |  | Not relevant |
| ESOMAR. Interviewing children & young people. Research ethics and social sciences |  |  |  | X |  |
| Gesellschaft für Informatik, Ethical Guidelines. Research ethics and social sciences |  |  |  | X |  |
| Association for Institutional Research, Code of Ethics. Research ethics and social sciences |  |  |  | X |  |
| Article 29 Working Group - Opinion 1/2002 on the CEN/ISSS Report on Privacy Standardisation in Europe, May 2002. Research involving data processing |  |  |  | X |  |
| Article 29 Working Group - Working Document: Processing of Personal Data on the Internet, February 1999. Research involving data processing |  |  |  | X |  |
| Committee for Medicinal Products for Human Use (CHMP-EMEA) Guideline on clinical trials in small populations, CHMP/EWP/83561/2005. Research involving medical intervention |  |  |  | X |  |
| European Commission - DG ENTERPISE - Detailed guidance for the request for authorisation of a clinical trial on a medicinal product for human use to the competent authorities, notification of substantial amendments and declaration of the end of the trial (revision 2), as required by Article 9 (8) of Directive 2001/20/EC , 2005. Research involving medical intervention |  |  |  | X |  |
| European Commission - DG ENTERPISE - Detailed guidance on the application format and documentation to be submitted in an application for an Ethics Committee opinion on the clinical trial on medicinal products for human use (revision 1) as required by Article 8 of Directive 2001/20/EC , 2006. Research involving medical intervention |  |  |  | X |  |
| Nuremberg Code. Research ethics and social sciences |  |  |  | X |  |
| Optional Protocol to the International Covenant on Civil and Political Rights. Research involving medical intervention |  |  |  | X |  |
| UNESCO Universal Declaration on the Human Genome and Human Rights 1997. Research involving data processing |  |  |  | X |  |
| Universal Declaration of Human Rights. United Nations on 10 December 1948. Research involving medical intervention |  |  |  | X |  |
| WHO (2000) Operational Guidelines for Ethics Committees that Review Biomedical Research. Geneva: World Health Organisation. Research ethics and social sciences |  |  |  | X |  |
| The ethical justification for inclusion of neonates in pragmatic randomized clinical trials for emergency newborn care | X |  |  |  | Not relevant |
| WHO Guidelines for Good Clinical Practice (GCP) for Trials on Pharmaceutical Products. Annex 3 of The Use of Essential Drugs. Sixth Report of the WHO Expert Committee. Geneva: World Health Organization, 1995: 97-137. Research involving medical intervention |  |  |  | X |  |
| World Medical Association, Declaration of Lisbon on the Rights of the Patient. Adopted by the 34th World Medical Assembly, Lisbon, Portugal, September/October 1981 and amended by the 47th General Assembly, Bali, Indonesia, September 1995. Research involving medical intervention |  |  |  | X |  |
| United Nations Convention on the Rights of the Child of 20 November 1989. Research involving children |  |  |  | X |  |
| MRS. Qualitative Research Guidelines. Research ethics and social sciences | X |  |  |  | Not relevant |
| MRS. Questionnaire Design Guidelines. Research ethics and social sciences |  |  |  | X |  |
| MRS. The Responsibilities of Interviewers. Research ethics and social sciences |  |  |  | X |  |
| UNICEF Evaluation Office, Children Participating in Research, Monitoring and Evaluation (M&E) - Ethics and Your Responsibilities as a Manager, Evaluation Technical Notes, No.1, April 2002. Research ethics and social sciences | X |  |  |  | Not relevant |
| Screening for Intimate Partner Violence, Elder Abuse, and Abuse of Vulnerable Adults. US Preventive Services Task Force. Final Recommendation Statement | X |  |  |  | Not relevant |
| UK Evaluation Society, Good Practice Guidelines. Research ethics and social sciences |  |  |  | X |  |
| UK Medical Research Council (MRC), Personal Information in Medical Research. Research involving medical intervention |  |  |  | X |  |
| Ethical Practice: Principles and Guidelines for Research with Vulnerable Individuals and Families PRAXIS: Research from the Centre for Children & Families in the Justice System. Research ethics and social sciences |  |  |  | X |  |
| Guidance on the Privacy and Electronic Communications Regulations 2003. Research ethics and social sciences |  |  |  | X |  |
| Council of American Survey Research Organisations (CASRO), CASRO Code of Standard and Ethics for Survey Research. Research ethics and social sciences |  |  |  |  |  |
| 25/05/2000. Optional Protocol to the Convention on the Rights of the Child on the involvement of children in armed conflicts. Research involving children | X |  |  |  | Not relevant |
| National Committee for Ethics in Social Science Research in Health (NCESSRH), Ethical Guidelines for Social Science research in Health. Research ethics and social sciences |  |  |  | X |  |
| Ethical Principles of Research in the Humanities and Social and Behavioural Sciences and Proposals for Ethical Review (2009) |  |  |  | X |  |
| National Statement on Ethical Conduct in Human Research, Qualitative Methods (2015) |  |  |  | X |  |
| Geriatric Research Policy: Japan Clinical Oncology Group (JCOG) policy | X |  | X |  | Not relevant |
| Osteoporosis in Frail Older Adults: Recommendations for Research from the ICFSR Task Force 2020 |  | X | X |  | Not relevant |
| Designing pharmaceutical trials for sarcopenia in frail older adults: EU/US Task Force recommendations |  |  | X |  | Not relevant |
| Developing ethics guidance for HIV prevention research: the HIV Prevention Trials Network approach | X |  |  |  | Not relevant |
| Integrating Comprehensive Geriatric Assessment for people with COPD and frailty starting pulmonary rehabilitation: the Breathe Plus feasibility trial protocol |  |  | X |  | Not relevant |
| Ethics in occupational health: deliberations of an international workgroup addressing challenges in an African context | X | X |  |  | Not relevant |
| Public Health Service Act (1993). General | X |  |  |  | Not relevant |
| Guidelines for Assessing the Sources of Risk and Vulnerability | X |  |  |  | Not relevant |
| Reichsrundschreiben 1931: pre-Nuremberg German regulations concerning new therapy and human experimentation | X |  |  |  | Not relevant |
| Guidelines for the use of placebo controls in clinical trials of psychopharmacologic agents. Loma Linda University Institutional Review Board | X |  |  |  | Not relevant |
| Principles underlying a model policy on relationships between staff and service recipients in a mental health system | X |  |  |  | Not relevant |
| HIV and Aging: Overcoming Challenges in Existing HIV Guidelines to Provide Patient-Centered Care for Older People with HIV | X |  | X |  | Not relevant |
| Modernizing Clinical Trial Eligibility Criteria: Recommendations of the American Society of Clinical Oncology-Friends of Cancer Research Organ Dysfunction, Prior or Concurrent Malignancy, and Comorbidities Working Group |  |  | X |  | Not relevant |
| Guidelines for support of orphaned and vulnerable children being cared for by their grandparents in the informal settlements of Mbabane, Swaziland | X |  |  |  | Not relevant |
| Cardiovascular diseases in women: a statement from the policy conference of the European Society of Cardiology | X |  |  |  | Not relevant |
| Nutrition and Physical Activity Clinical Practice Guidelines for Older Adults Living with Frailty | X |  | X |  | Not relevant |
| The Asia-Pacific Clinical Practice Guidelines for the Management of Frailty | X |  | X |  | Not relevant |
| Physical Frailty: ICFSR International Clinical Practice Guidelines for Identification and Management | X |  | X |  | Not relevant |
| European Diabetes Working Party for Older People 2011 Clinical Guidelines for Type 2 Diabetes Mellitus. Executive Summary | X |  | X |  | Not relevant |
| TCES – North West London Safeguarding Children & Vulnerable Young Adults Policy and Procedures | X |  |  |  | Not relevant |
| TCES – East London Safeguarding Children & Vulnerable Young Adults Policy and Procedures | X |  |  |  | Not relevant |
| AGS Position Statement: Making Medical Treatment Decisions for Unbefriended Older Adults | X |  |  |  | Not relevant |
| Dementia Research Fit for the Planet: Reflections on Population Studies of Dementia for Researchers and Policy Makers Alike | X |  | X |  | Not relevant |
| Multilevel Interventions Targeting Obesity: Research Recommendations for Vulnerable Populations | X |  |  |  | Not relevant |
| End-of-Life Communication Needs for Adolescents and Young Adults with Cancer: Recommendations for Research and Practice | X |  |  |  | Not relevant |
| Use of Performance-Enhancing Substances. American Academy of Pediatrics. Policy Statement. | X |  |  |  | Not relevant |
| Screening for and Managing the Person with Frailty in Primary Care: ICFSR Consensus Guidelines | X |  | X |  | Not relevant |
| Exercise, aging and frailty: guidelines for increasing function. | X |  | X |  | Not relevant |
| Best practice guidelines for the management of frailty: a British Geriatrics Society, Age UK and Royal College of General Practitioners report | X |  | X |  | Not relevant |
| National response to orphans and other vulnerable children in sub-Saharan Africa: The OVC Policy and Planning Effort Index, 2004 | X |  |  |  | Not relevant |
| Health Care Policy For Medically Fragile Children | X | X |  |  | Not relevant |

- 382 documents excluded.
- 79 final documents included.
