# Supplementary material for: Vulnerability in research ethics: A systematic review of policy guidelines and documents
Source: PLoS One. 2025 Jul 1;20(7):e0327086. doi: 10.1371/journal.pone.0327086 (PMC12212517; doi:10.1371/journal.pone.0327086)
Supplement: S3 Table — (DOCX) [file pone.0327086.s003.docx]

**S3 Table: Comprehensive overview integrating the most relevant findings**

| **RESEARCH QUESTIONS** | **NUMBER OF DOCUMENTS** |
| --- | --- |
| *Meaning and definition of vulnerability* | |
| Diminished ability to safeguard one's own interests | 1. (docs n° 30, *59* and *64*) |
| Increased likelihood of incurring additional harm/risk | 1. (docs n° *55* and *84*) |
| Inability to provide a valid informed consent | 1. (doc n° *81*) |
| A condition of disadvantage that depends on individual or group circumstances | 1. (doc n° *85*) |
| Limited decision-making capacity | 1 (doc n° *61*) |
| *Identification of vulnerable group/vulnerable populations* | |
| Group-based approach: lists of vulnerable groups/populations, without further explanationChildrenPeople with learning disabilities or cognitive impairmentPrisonersPeople with mental disabilitiesElderlySubordinatesPregnant womenPeople with serious illnesses | 12 (docs n° *16*, 26, *31*, *32*, *38*, *46*, *58*, *67*, *69*, *78*, *84* and *85)*  6 (docs n° *26*, *31*, 32, *67*, 77 and *78)*  6 (docs n° *17*, *26*, *67*, *79*, *85* and *88)*  6 (docs n° *17*, *26*, *31*, *69*, *75* and *85)*  5 (docs n° *31*, *32*, *38*, *75* and *78)*  5 (docs n° *26*, *32*, *58*, *69* and *78)*  5 (docs n° *17*, *67*, *75*, *79* and *88)*  3 (docs n° *67*, *75* and *84)* |
| Inability to provide free informed consent | 10 (docs n° *15*, 22, *23*, *25*, *27*, *35*, *62*, *64*, 70 and *71)* |
| Vulnerable individuals as exposed to undue influence | 7 (docs n° *20*, *28*, *44*, *53*, *56*, *86* and *89)* |
| Inability to protect one’s own interests | 3 (docs n° *29*, *59* and *76)* |
| Combination of different definitions | 10 (docs n° *12*, *24*, *42*, *48*, *51*, 60, *61*, *63*, *65* and *83*) |
| Original definition of vulnerable group/population | 7 (docs n° 30, *33*, 52, *54*, *66*, *73* and 80) |
| *Normative justifications for vulnerability* | |
| Only consent-based reason for vulnerability | 32 (docs n° *21-23*, *25*, *28*, *33*-*35*, *37*-*41*, *43*-*45*, *47*, 50, *52-54*, *56*, *57*, *66*, *68*, *73*, *78*, *79*-*81*, *86*, and *89*) |
| Only harm-based reason for vulnerability | 3 (docs n° *18*, *19* and *88)* |
| Only justice-based reason for vulnerability | 0 |
| “Consent-based” + “harm-based” reasons for vulnerability | 21 (docs n° *13, 16, 26, 27, 29, 36, 42, 48, 58-63, 65, 69-71, 76, 83,* and *84)* |
| “Consent-based” + “justice-based” reasons for vulnerability | 6 (docs n° *1*, *13*, *16*, *32*, *82* and *85)* |
| “Consent-based” + “harm-based” + “justice-based” reasons for vulnerability | 6 (docs n° 20, *24*, 30, *51*, *55* and *62)* |
| *Provisions for vulnerable populations: first-level* | |
| Participation determined by a free and informed choice | 15 (docs n° *21, 25*, *27*, *28*, 30, 32, *33*, *36*, *47*, *52*, *54*, *61*, *66*, *76* and *78*) |
| Demonstrate the appropriateness of the inclusion criteria adopted in the research protocol | 13 (docs n° *1*, *12*, *27*, *29*, 30, *35-37*, *42*, *59*, *63*, *65* and *75*) |
| Fair distribution of burdens and benefit | 3 (docs n° *35*, *58* and *85*) |
| Grant the right to confidentiality of information | 3 (docs n° *24*, *32* and *69*) |
| People should benefit from the knowledge, practices and interventions resulting from research | 2 (docs n° *75* and *80*) |
| Ability to provide consent assessed on the basis of the personal characteristics of each individual | 1 (doc n° *47*) |
| *Provisions for vulnerable populations: Broad-grained second-level* | |
| Introduction of specific protections to safeguard the rights, safety and well-being of vulnerable participants | 35 (docs n° *16*, *17*, *19*, 20, *22*, *25*-*27*, *30*, *31*, *34*, *35*, *38*, *40*, *42*, *43*, *47*, *48*, *51*, *53*, *55*, *59-61*, *63*, *64*, *67*, *73*, *79*, 80, *85*-*89*) |
| Vulnerable people not exploited | 11 (docs n° *17*, *24*, *48*, *49*, *52*, *59*, *62*, *70*, *71*, *84* and *88*) |
| Justify the exclusion of vulnerable subjects | 7 (docs n° *17*, *37*, *55*, *67*, *69*, *76* and *85*) |
| Consider all factors contributing to vulnerability beforehand | 7 (docs n° *24*, *26*, *27*, *48*, *55*, *59* and *85*) |
| Avoid an excessive and systematic exclusion | 3 (docs n° *18*, *37* and *55*) |
| Exclusion under special conditions | 2 (docs n° *38* and *40*) |
| *Provisions for vulnerable populations: fine-grained second level* | |
| Research on vulnerable subjects only when it cannot be conducted on non-vulnerable individuals, and when it responds to their specific needs | 15 (docs n° *16*, *19*, *22*, *24*, *30*, *55*, *58-61,* *64*, *65*, *76*, *81* and *82*) |
| Proxy consent or consent provided by a legal guardian necessary when subjects are unable to consent first-hand | 13 (docs n° *21*, *22*, *26*, *27*, *32*, *36*, *43*, *51*, *54*, *55*, *66*, *69* and *80*) |
| Involve at least one member who has experience in working with vulnerable people and/or their advocate in the ethical committee | 12 (docs n° *14*, *29*, 30, *46*, *52*, *55*, 60, *64*, *65*, *67*, *86* and *89*) |
| Research on vulnerable individuals only if it can be conducted in the same way on legally competent individuals | 6 (docs n° *23*, *38*, *48*, 69, *81* and *82*) |
| No more than minimal risk | 6 (docs n° *27*, *48*, *55*, *66*, *78* and *85*) |
| Research involving minors only when there are tangible benefits for the child and when parental consent has been sought | 5 (docs n° *28*, *32*, *46*, *59* and *60*) |
| Constantly monitor the study and its effects | 5 (docs n° *24*, *51*, *77*, *82* and *88*) |
| The least vulnerable of a specific category subjects should be included | 3 (docs n° *26*, *37* and *48*) |
| Respect the tradition of societies where proxy consent is culturally widespread, but requiring individual consent | 1 (doc n° *28*) |
