# Supplementary material for: Vulnerability in research ethics: A systematic review of policy guidelines and documents
Source: PLoS One. 2025 Jul 1;20(7):e0327086. doi: 10.1371/journal.pone.0327086 (PMC12212517; doi:10.1371/journal.pone.0327086)
Supplement: S1 Appendix — (DOCX) [file pone.0327086.s006.docx]

**S4 Appendix: QUAGOL Scheme**

12. A Model Regulatory Program for Medical Devices: An International Guide

2001

KEYWORDS:

| Vuln- | Frag- | Frail- |
| --- | --- | --- |
| x |  |  |

*Undue influence occurs through an offer of an excessive, unwarranted, inappropriate or improper reward or other overture in order to obtain compliance. Also, inducements that would ordinarily be acceptable may become undue influences if the subject is especially vulnerable.*

*Assessment of the justifiability of research should reflect at least the following considerations: when vulnerable populations are involved in research, the appropriateness of involving them should itself be demonstrated. A number of variables go into such judgments, including the nature and degree of risk, the condition of the particular population involved, and the nature and level of the anticipated benefits.*

*One special instance or injustice results from the involvement of vulnerable subjects. Certain groups, such as racial minorities, the economically disadvantaged, the very sick and the institutionalized may continually be sought as research subjects, owing to their ready availability in settings where research is conducted. Given their dependent status and their frequently compromised capacity for free consent, they should be protected against the danger of being involved in research solely for administrative convenience, or because they are easy to manipulate as a result of their illness or socioeconomic condition.*

13. A Proposal for the Retrospective Identification and Categorization of Older People With Functional Impairments in Scientific Studiesd: Recommendations of the Medication and Quality of Life in Frail Older Persons (MedQoL) Research Group

2018

KEYWORDS:

| Vuln- | Frag- | Frail- |
| --- | --- | --- |
| x |  | x |

*It is accepted that frailty can be considered a vulnerable state that often results in disability, loss of autonomy, and dependency.*

14. AGS Report on Engagement Related to the NIH Inclusion Across the Lifespan Policy

2019

KEYWORDS:

| Vuln- | Frag- | Frail- |
| --- | --- | --- |
| x |  | x |

*The Inclusion Across the Lifespan workshop emphasized the need for a paradigm shift from “protecting vulnerable subjects from research” to “protecting vulnerable subjects through research.” It is important that institutional review board members, researchers, and potential research subjects understand that underrepresenting older adults and those with multimorbidity in clinical research results in potentially unsafe and inappropriate care decisions.*

1. Belmont Report

1979

KEYWORDS:

| Vuln- | Frag- | Frail- |
| --- | --- | --- |
| x |  |  |

*Assessment of the justifiability of research should reflect at least the following considerations: when vulnerable populations are involved in research, the appropriateness of involving them should itself be demonstrated. A number of variables go into such judgments, including the nature and degree of risk, the condition of the particular population involved, and the nature and level of the anticipated benefits.*

*One special instance of injustice results from the involvement of vulnerable subjects. Certain groups, such as racial minorities, the economically disadvantaged, the very sick, and the institutionalized may continually be sought as research subjects, owing to their ready availability in settings where research is conducted. Given their dependent status and their frequently compromised capacity for free consent, they should be protected against the danger of being involved in research solely for administrative convenience, or because they are easy to manipulate as a result of their illness or socioeconomic condition.*

15. Best Practices for Protecting Privacy in Health Research

2005

KEYWORDS:

| Vuln- | Frag- | Frail- |
| --- | --- | --- |
| x |  |  |

*Ethical consideration around research involving those who are not competent to give a free and informed consent on their own behalf must seek to balance (1) the vulnerability that arises from their incompetence with (2) the injustice that would arise from their exclusion from the benefits of research*.

16. Clinical Investigation of Medicinal Products in the Paediatric Population (E11)

1999

KEYWORDS:

| Vuln- | Frag- | Frail- |
| --- | --- | --- |
| x |  |  |

*The pediatric population represents a vulnerable subgroup. Therefore, special measures are needed to protect the rights of pediatric study participants and to shield them from undue risk.*

*Information that can be obtained in a less vulnerable, consenting population should not be obtained in a more vulnerable population or one in which the patients are unable to provide individual consent.*

17. Clinical Trials and Biomedical Research

2006

KEYWORDS:

| Vuln- | Frag- | Frail- |
| --- | --- | --- |
| x |  |  |

*Researchers must ensure that the vulnerable not be exploited and that eligible candidates who may benefit from participation not be excluded without good cause.*

*Safeguards for vulnerable subjects When vulnerable groups, such as prisoners, pregnant mothers, mentally disabled, and the economically and educationally disadvantaged are the participants selected for the research, particular cognisance should be taken of their rights and the safe-guarding of their rights. Special attention is given to situations where the consent from the next of kin, guardian or legal representatives, are sought.*

18. Conducting Science in Disasters: Recommendations from the NIEHS Working Group for Special IRB Considerations in the Review of Disaster Related Research

2016

KEYWORDS:

| Vuln- | Frag- | Frail- |
| --- | --- | --- |
| x |  | x |

*Although the Code of Federal Regulations does establish research protections for certain groups such as children, prisoners, women, and fetuses, there is no explicit protection for potentially vulnerable disaster survivor research participants.*

*Justice demands that research be carried out for the benefit of the population as a whole; therefore, systematic exclusion of protected or vulnerable groups from disaster research studies should be avoided. Failure to include these groups leaves a knowledge gap in our understanding of the impact of disasters across the entire population.*

*IRBs can play useful roles in achieving careful, balanced, thoughtful procedures that both consider the value of the research to advance science and reduce suffering—and that also consider the potential for harm based on the unique vulnerabilities of disaster survivors in a disaster aftermath.*

19. Declaration of Helsinki

2024

KEYWORDS:

| Vuln- | Frag- | Frail- |
| --- | --- | --- |
| x |  |  |

*Vulnerable Groups and Individuals*

*Some individuals, groups, and communities are in a situation of more vulnerability as research participants due to factors that may be fixed or contextual and dynamic, and thus are at greater risk of being wronged or incurring harm. When such individuals, groups, and communities have distinctive health needs, their exclusion from medical research can potentially perpetuate or exacerbate their disparities. Therefore, the harms of exclusion must be considered and weighed against the harms of inclusion. In order to be fairly and responsibly included in research, they should receive specially considered support and protections.*

*Medical research with individuals, groups, or communities in situations of particular vulnerability is only justified if it is responsive to their health needs and priorities and the individual, group, or community stands to benefitt from the resulting knowledge, practices, or interventions. Researchers should only include those in situations of particular vulnerability when the research cannot be carried out in a less vulnerable group or community, or when excluding them would perpetuate or exacerbate their disparities.*

*Those persons incapable of giving free and informed consent are in situations of particular vulnerability and are entitled to the corresponding safeguards. In addition to receiving the protections for the particularly vulnerable, those incapable of giving consent must only be included if the research is likely to either personally benefit them or if it entails only minimal risk and minimal burden.*

20. Doing No Harm and Getting It Right: Guidelines for Ethical Research with Immigrant Communities

2013

KEYWORDS:

| Vuln- | Frag- | Frail- |
| --- | --- | --- |
| x | x |  |

*Under Common Rule guidelines, extra protections for the rights and welfare of these vulnerable research participants are stressed, as they are particularly susceptible to “coercion and undue influence” as well as to breaches of confidentiality.*

*While immigrant populations have not been specifically designated as “vulnerable”, we argue that many immigrant children, adolescent, and their families fall into this designation for a variety of reasons. Many immigrant children, adolescents, and their families face a number of additional vulnerabilities including high rates of poverty and low levels of parental education.*

*We need to be ready to protect vulnerable participants beyond the requirements of lax IRBs who may have not yet given consideration to this population as well as to anticipate the requirements of hypervigilant IRBs who may arguably be taking on overly restrictive precautions.*

*An additional precaution that has become more prevalent to protect confidentiality for vulnerable populations has been the Certificate of Confidentiality (COC) administered by the National Institutes of Health (NIH). The COC aims to protect the privacy of research subjects by protecting investigators and institutions from being compelled to release information that could be used to identify subjects with a research project.*

21. Doing the Right Thing: Outlining the DWP's approach to ethical and legal issues in social research

2003

KEYWORDS:

| Vuln- | Frag- | Frail- |
| --- | --- | --- |
| x |  | x |

*For some projects, for example where the contractor is using a field force of interviewers, it may be necessary for the interviewer briefing to cover issues such as the importance of ensuring that consent to participate is an informed choice and/or dealing sensitively with vulnerable groups.*

*These are groups where research participants are particularly vulnerable due to factors such as age, disability, physical or mental ill-health. Where there are particular difficulties gaining fully informed consent, it may be necessary to use a proxy, such as a carer, instead.*

22. Ethical Aspects of Clinical Research in Developing Countries

2003

KEYWORDS:

| Vuln- | Frag- | Frail- |
| --- | --- | --- |
| x |  |  |

*Persons who are incapable of giving legal consent to clinical trials should be given special protection. Children represent a vulnerable population with developmental, physiological and psychological differences from adults, which make age- and development- related research important for their benefit.*

*In the case of other persons incapable of giving their consent, such as persons with dementia, psychiatric patients, etc., inclusion in clinical trials in such cases should be on an even more restrictive basis. Moreover, in such cases the written consent of the patient's legal representative, given in cooperation with the treating doctor, is necessary before participation in any such clinical trial.*

23. Ethical considerations for Clinical Trials on Medical Products conducted with the Paediatric Population

2008

KEYWORDS:

| Vuln- | Frag- | Frail- |
| --- | --- | --- |
| x |  |  |

*The neonate represents the most vulnerable of all paediatric age groups and requires even more careful review. Children represent a vulnerable population with developmental, physiological and psychological differences from adults, which make age- and development- related research important for their benefit.*

*The recommendations in the document aim to contribute to the promotion and protection of the dignity, the well-being and the rights of children (minors) all of whom are vulnerable and unable to give informed consent.*

*Because of the special protection they deserve, children should not be the subject of clinical trials when the research can be done in legally competent subjects. If research with children proves necessary, the least vulnerable among them should usually be included (i.e. older children). If there is a necessity to subject children to a clinical trial, the choice of subsets of the paediatric population to be included should be made on the basis of the likely target population for the medicine being tested, the possibility of extrapolation, and the scientific validity of such an approach*

24. Ethical Considerations in Biomedical HIV Prevention Trials

2012

KEYWORDS:

| Vuln- | Frag- | Frail- |
| --- | --- | --- |
| x |  |  |

*Generally, early clinical phases of biomedical HIV prevention research should be conducted in communities that are less vulnerable to harm or exploitation, usually within the sponsor country.*

*Guidance Point 8: Vulnerable Populations The research protocol should describe the social contexts of a proposed research population (country or community) that create conditions for possible exploitation or increased vulnerability among potential trial participants, as well as the steps that will be taken to overcome these and protect the rights, the dignity, the safety, and the welfare of the participants.*

*Establishing a biomedical HIV prevention product development programme that entails the conduct of some, most, or all of its clinical trial components in a country or community that is relatively vulnerable to harm or exploitation is ethically justified if:*

- *the product is a vaccine anticipated to be effective against a strain of HIV that is an important public health problem in the country;*
- *the country and the community either have, or with assistance can develop or be provided with, adequate scientific and ethical capability and administrative and health infrastructure for the successful conduct of the proposed research;*
- *community members, policy makers, ethicists, and investigators in the country have determined that their residents will be adequately protected from harm and exploitation, and that the biomedical HIV prevention product development programme is necessary for and responsive to the health needs and priorities in their country;*
- *all other conditions for ethical justification as set forth in this document are satisfied.*

*Social and cultural factors should be considered to determine the vulnerability within the community of individuals who are either included or excluded. In particular, gender-sensitive approaches are key when designing recruitment procedures and special attention needs to be paid to the inclusion or exclusion of pregnant women.*

*Examples of populations that may have an increased vulnerability include women, children and adolescents, men who have sex with men, injecting drug users, sex workers, transgender persons, indigenous populations, the poor, the homeless, and communities from resource-poor settings in high-income and low- and middle-income countries. At the same time, it is precisely these populations who stand to benefit most from the successful development of a new biomedical HIV prevention product or method. For these reasons, it is imperative to ensure protection of the rights of participants in biomedical HIV prevention trials, and respect for their dignity, safety, and welfare. A social and political analysis should be carried out early on in planning the research process, to assess determinants of vulnerability, such as poverty, gender, age, ethnicity, sexuality, health, employment, education, and legal conditions in potential participating communities. Research protocols might also include ongoing independent monitoring of a trial in relation to its impact on the vulnerabilities of communities participating in the study.*

*The following are individuals or groups who should be given extra consideration with regard to their ability to voluntarily participate in biomedical HIV prevention trials:*

- *persons who are junior or subordinate members of hierarchical structures, who may be vulnerable to undue influence or coercion and may fear retaliation if they refuse cooperation with authorities, including members of the armed forces, students, government employees, prisoners, and refugees;*
- *persons who engage in illegal or socially stigmatised activities, who are vulnerable to undue influence and threats presented by possible breaches of confidentiality and action by law enforcement authorities, including sex workers, injecting drug users, and men who have sex with men;*
- *persons who are impoverished or dependent on welfare programmes, who are vulnerable to being unduly influenced by offers of what others may consider modest material or health inducements.*

*All participants are entitled to confidentiality of information disclosed or discovered in the recruitment and informed consent processes, and during conduct of the trial. Community involvement should not compromise the confidentiality of study participants. This is of particular importance with respect to participants from vulnerable populations, women and adolescents, who may be socially susceptible to stigma and discrimination.*

*People who inject drugs suffer several layers of vulnerability. Criminalization of their drug use renders them vulnerable to punitive, often harsh, law enforcement practices including incarceration. They may experience additional vulnerability because of generalized stigma and discrimination, including from some health care professionals and policy-makers; personal mental health issues, preceding or resulting from their drug use; poverty; racism, if they are members of certain racially-defined groups; and marginalization. HIV prevention trials should not be conducted in compulsory drug detention centres*.

25. Ethical Guidelines

2003

KEYWORDS:

| Vuln- | Frag- | Frail- |
| --- | --- | --- |
| x |  |  |

*A major limitation upon gaining informed consent lies with “vulnerable” populations. Such groups include children, those with an intellectual disability, or those in a dependent relationship to the researcher or commissioning body. College students, for example, are a frequently studied group who may find difficulty in resisting cooperation. In conducting research with vulnerable populations, extra care must be taken to protect their rights and ensure that their compliance is freely entered in to.*

26. Ethical Guidelines for Conducting Research Studies Involving Human Subjects

2013

KEYWORDS:

| Vuln- | Frag- | Frail- |
| --- | --- | --- |
| x |  |  |

*Vulnerable groups:*

*Rights and welfare of mentally challenged and mentally differently able persons who are incapable of giving informed consent or those with behavioural disorders must be protected. Appropriate proxy consent from the legal guardian should be taken after the person is well informed about the study, need for participation, risks and benefits involved and the privacy and confidentiality procedures. The entire consent process should be properly documented. Involvement of participants such as prisoners, students, subordinates, employees, and service personnel etc. who have reduced autonomy as research participants, since the consent provided may be under duress or various other compelling reasons required adequate justification.*

*Careful consideration should be given to protect the dignity, safety and welfare of the participants when the social contexts of the proposed research can create foreseeable conditions for exploitation of the participants or increase their vulnerability to harm. Generally, early clinical phases of research, particularly of drugs, vaccines and devices, should be conducted in communities that are less vulnerable to harm or exploitation.*

*For choosing vulnerable group like children, care should be taken to choose the particular age with regard to gender, ethnic background and health profile for testing vaccines for this age especially if they are from over-researched community.*

*While obtaining data/samples from vulnerable subgroups with reduced autonomy, Ethics Committee should ensure that informed consent be obtained from legally authorized representatives in the presence of impartial witness. The risks and benefits should be adequately explained.*

1. Ethical Guidelines for Research on Human Subject in Thailand

2007

KEYWORDS:

| Vuln- | Frag- | Frail- |
| --- | --- | --- |
| x |  |  |

*Principle of Respect for Person The principle covers the following aspects: respect for vulnerable persons. Respect for human dignity leads to an ethical requirement for vulnerable people who have inferior or lack physical capacities or have diminished capacities for making a reasonable decision, such as children, pregnant women, psychiatric patients, unconscious patients, and prisoners. These vulnerable people need to be protected from being forced to participate in a research involuntarily. In practice, a special treatment is needed for the protection of their benefits.*

*Research Studies in Vulnerable Subjects Vulnerable groups of people are those who need to depend on others and are unable to express their opinion freely or to make their own decisions. These include hospitalized patients, prisoners, children, the mentally impaired, critically ill patients, psychotic patients, pregnant women, and the economically disadvantaged. They are easily taken advantage of. As a consequence, the protection of vulnerable groups of people is of prime importance. Investigators should not select these groups of people simply because of easy management or convenience for the conduct of a research study due to their economic or health constraints. However, if a valid need exists to conduct a study on these people, some recommendations should be followed:*

- *An irrefutable rationale for conducting research in these population groups should be clearly explained in the protocols.*
- *Precautions against possible physical and mental harm should be exercised especially when the study is conducted on children.*
- *The research procedures used in the study should be appropriate for the specific groups of these people.*
- *In a study involving pregnant women, adequate information on the safety and impacts to the fetus should beprovided.*
- *In a study involving minors, psychiatric patients, or the incompetent, the informed consent should be obtained from their parents, or guardians or legal representatives.*
- *It should be ensured that parents, guardians, or legally acceptable representatives are fully informed about the study.*
- *The rights of the minors and the economically disadvantaged should be respected for making their voluntary decisions.*
- *It should be shown that the research participants have freedom in voluntarily participating in a research study, including for example a research study conducted on prisoners, inmates, and refugees.*
- *Precautions against harm and protection of confidentiality should be strictly exercised when conducting research studies in subjects with illegal careers, such as sex workers or illegal drug users.*
- *In the case where a study is conducted on the vulnerable people without direct health benefit to them, the possible risks should not be greater than the minimal risk normally found in a general physical or psychiatric examination, or unless the research ethics committee allows for a greater-than-minimal risk.*

1. Ethics Guidelines for Human Biomedical Research

2015

KEYWORDS:

| Vuln- | Frag- | Frail- |
| --- | --- | --- |
| x |  |  |

*For research involving vulnerable persons not lacking mental capacity (for example, prisoners, uniformed personnel, and employees), consent should be taken by independent third parties, whenever possible. When it is not possible for consent to be taken by an independent third party, the IRB may give directions for the consent to be taken by the researcher so long as there are provisions to manage the conflict of interest and sufficient safeguards to protect the welfare and interests of the participants.*

*Consent Involving Vulnerable Persons While it is usual to treat the individual as an autonomous agent for purposes of taking consent, provision has to be made when considering research participants who are vulnerable. Such participants include:*

- *persons lacking mental capacity (such as the intellectually disabled, people who are incapacitated through accident, injury or illness, and others as defined in the Mental Capacity Act);*
- *those whose autonomy might be prejudiced by being under the influence of, or the control of, or obligated to, third parties;*
- *minors.*

*Consent for Research Involving Vulnerable Persons Not Lacking Mental Capacity Vulnerable research participants not only include those who are lacking mental capacity, but also those whose autonomy might be prejudiced by being under the influence or control of, or by being obligated to, third parties. Potentially vulnerable participants might include, but are not limited to: Prisoners, Uniformed personnel, especially junior ranks, Patients, especially if the intending researcher is their attending physician; and Employees, junior collaborators, or students. A further issue of vulnerability arises in societies where social proxy arrangements are widespread, for example, where a village headman might be thought to have the authority to give consent on behalf of a village, or a husband on behalf of a wife. Not all societies treat their individual members as autonomous. This can become an issue if researchers based in Singapore seek to conduct research in places where social proxy arrangements are widespread. In such cases, while local customs are to be respected, they cannot supersede a requirement for individual consent.*

*Children present certain consent issues if involved in research, and they are categorised as a vulnerable class of research participants. In some jurisdictions a distinction is made between consent and assent, such that if parents consent, research can proceed provided children assent, i.e. agree. The assent of a child is not comparable to the informed consent of an adult. It is perhaps better regarded as a mechanism for engaging the child in the research process, in such a way as to respect the child’s right to object, and to entitle them to as reasonable an explanation as may be reasonable, consistent with the child’s level of understanding, but without an implication that the child is giving informed consent. In clinical research that has a reasonable expectation of benefitting a child, the research might be allowed to proceed even without the child’s assent, if the parents give consent, but in general, researchers should respect refusal by a child.*

1. Ethics in clinical research: the Indian perspective

2011

KEYWORDS:

| Vuln- | Frag- | Frail- |
| --- | --- | --- |
| x |  |  |

*VULNERABLE POPULATION Persons who are relatively or absolutely incapable of protecting their own interests are termed as vulnerable research population. The very poor, illiterate patients, children, individuals with questionable capacity to give consent (including psychiatric patients), prisoners, foetuses, pregnant women, terminally ill patients, students, employees, comatose patients, tribals and the elderly are examples of vulnerable population. Declaration of Helsinki states that ‘Medical research involving a underprivileged or vulnerable population or community is only justified if the research is responsive to the health needs and priorities of that population or community and if there is a reasonable likelihood that this population or community stands to benefit from the results of the research.’ It is the responsibility of the EC to see whether the inclusion of vulnerable populations in the study is justifiable or the population is just being exploited to generate clinical data. To prevent even minor exploitation the EC should consult the representative of vulnerable population that is to be researched upon while reviewing the protocol.*

1. Ethics in Health Research: Principles, Structures, and Processes

2015

KEYWORDS:

| Vuln- | Frag- | Frail- |
| --- | --- | --- |
| x |  |  |

*The rationale for the inclusion and exclusion criteria must be clear, explicit and reasonable. If vulnerable participants are to be included, an adequate justification should be provided; protective safeguards and measures should be explained.*

*Personal circumstances, such as mental or intellectual impairment, acute illness, advanced age, and pregnancy and childbirth may increase vulnerability. Persons may be factually incapable or less capable of understanding information and processing it to reach a decision e.g. about whether to participate in research. Environmental circumstances may also increase vulnerability such as very poor socio-economic conditions, low levels of formal education and literacy, or restricted access to health care services. Such persons may be more easily persuaded to agree to participate without a properly considered understanding of the implications.*

*Particular caution should be exercised before undertaking research involving participants in such communities, and RECs should ensure that:*

- *persons in these communities are not being involved in research merely because they are expediently accessible, while the research could be carried out in a less vulnerable community;*
- *the research is relevant to the health needs and priorities of the community in which it is to be carried out;*
- *research participants know they will take part in research; and that the research will be carried out only with their consent. Particular attention should be given to the content, language(s) and procedures used to obtain informed consent.*

*Factual information is required before deciding that a community is too vulnerable to be invited to choose whether to participate in research. In order to ensure optimal protection of vulnerable participants, the REC may impose additional protective measures for the informed consent process; or require increased monitoring and interim reporting on participants’ welfare; or require post-recruitment reviews of the effectiveness of the protective measures imposed. Other measures may also be appropriate. Note that the decision to impose additional measures should flow from an assessment of the nature of the research and the circumstances of the potential participants. In other words, additional protective measures should not be automatic just because a vulnerable group will be recruited; rather, the decision should be based on the particular circumstances of the proposal before the REC. For example, an automatic assumption that impoverished people cannot choose responsibly whether to participate in research is disrespectful because it denies their autonomy.*

*If compliance with the additional measures is poor and participants’ welfare is negatively affected, approval for the study may be withdrawn, temporarily or permanently, as the case may be. Groups of participants discussed here include:*

- *minors (children and adolescents)*
- *women*
- *adults with incapacity to provide informed consent*
- *persons in dependent relationships*
- *persons highly dependent on medical care*
- *persons with physical disabilities*
- *prisoners*
- *collectivities.*

*Prisoners The chief reason to consider prisoners as a vulnerable class of persons is the potential effect of incarceration on the voluntariness of the decision to participate in research. Neither coercion (direct threat of negative sanction) nor undue influence is acceptable in the informed consent process.*

*The REC should include, at least on an ad hoc basis, a member with experience and knowledge of working with prisoners when deliberating on the protocol.*

*Vulnerability – diminished ability to fully safeguard one’s own interests in the context of a specific research project; may be caused by limited capacity or limited access to social goods like rights, opportunities and power.*

1. EU-Code for Ethics for Socio-Economic Research

2004

KEYWORDS:

| Vuln- | Frag- | Frail- |
| --- | --- | --- |
| x |  |  |

*Special care should be taken to protect the interests of members of vulnerable groups such as children, older people and those with learning or other disabilities.*

*Issues of social harm are not restricted to women: other vulnerable groups might include torture victims, the elderly, people with an incomplete grasp of the language in which they are being interviewed, or people with mental disabilities.*

1. Framework for Research Ethics

2015

KEYWORDS:

| Vuln- | Frag- | Frail- |
| --- | --- | --- |
| x |  |  |

*Research potentially requiring a full ethics review Research involving potentially vulnerable people, for example children and young people, those with a learning disability or cognitive impairment, or potentially vulnerable individuals in a dependent or unequal relationship*

*Work with potentially vulnerable groups In most cases, researchers working with vulnerable people will need to secure Disclosure and Barring Service (DBS) clearance. The DBS offers organisations a means to check the criminal record of researchers to ensure that they do not have a history that would make them unsuitable for work involving children and vulnerable adults.*

*Proxy consent can be obtained by a person authorised to act on behalf of a vulnerable person. Where proxy consent for research participants is necessary, the best interests of the vulnerable person should be of the highest importance. Proxy consent should only be used when participants are unable to consent themselves or where it is legally necessary. Care should be taken when consent cannot be sought from the participants, and it should not be assumed that agreement cannot be sought from children because of their age.*

*Limits to confidentiality Researchers should, when eliciting consent, make clear the limits to confidentiality, particularly when working with potentially vulnerable individuals or groups - for example when undertaking research with children, families and vulnerable populations, or individuals involved in illegal activities.*

*Where participants are in a potentially vulnerable or dependent position (eg children) it is important to ensure that they have the time and opportunity to access support in their decision-making, for example by discussing their choice with a trusted adult. Where consent is sought from children it is normally good practice to secure permission from a responsible adult in addition to child consent. Where participants are not literate verbal consent may be obtained, but this should wherever possible include a recorded written witness sign-off.*

*How do you obtain consent from potentially vulnerable people? In cases where research involves potentially vulnerable groups, for example children, older persons or adults with learning disabilities should be avoided wherever possible, and every effort should be made to develop methods of seeking consent that are appropriate to the groups being studied, using expert advice, support and training where necessary.*

1. Framework of Guidelines for Research in the Social Sciences and Humanities in Malawi

2011

KEYWORDS:

| Vuln- | Frag- | Frail- |
| --- | --- | --- |
| x |  |  |

*All persons have the right to individual autonomy and self determination. Some of them are vulnerable in the sense that they have a compromised autonomy related to decisions about research participation to a degree that would violate the principle of respect for persons. Therefore, any individual who is invited to participate in a research study shall be given an adequate description of the study that is clear and complete enough for the individual to judge whether she or he wants to participate. The informed consent process is designed to provide potential participants with readily understandable information in an amount and timing appropriate to achieve the participant’s understanding. Consent shall be obtained from each research participant who is legally, mentally and physically able. For those that are not, including minors, permission shall be sought from parents or legal guardians or any of their legally authorized representatives as the situation may apply.*

*Examples of vulnerable populations include pregnant women, prisoners, orphans, people living with HIV and AIDS, refugees, persons with mental disabilities, the illiterate, and women and men who, in some settings, may have to ask their spouses before consenting to participate in a research.*

1. Good Clinical Practice Guidelines

2020

KEYWORDS:

| Vuln- | Frag- | Frail- |
| --- | --- | --- |
| x |  |  |

*Vulnerable Subjects Individuals whose willingness to volunteer in a clinical trial may be unduly influenced by the expectation, whether justified or not, of benefits associated with participation, or of a retaliatory response from senior members of a hierarchy in case of refusal to participate. Examples are members of a group with a hierarchical structure, such as medical, pharmacy, dental, and nursing students, subordinate hospital and laboratory personnel, employees of the pharmaceutical industry, members of the armed forces, and persons kept in detention. Other vulnerable subjects include patients with incurable diseases, persons in nursing homes, unemployed or impoverished persons, patients in emergency situations, ethnic minority groups, homeless persons, nomads, refugees, minors, and those incapable of giving consent.*

*An IRB/IEC should safeguard the rights, safety, and wellbeing of all trial subjects. Special attention should be paid to trials that may include vulnerable subjects.*

1. Good Clinical Practice Guidelines for Clinical Research in India

2001

KEYWORDS:

| Vuln- | Frag- | Frail- |
| --- | --- | --- |
| x |  |  |

*Vulnerable groups Effort may be made to ensure that individuals or communities invited for research be selected in such a way that the burdens and benefits of the research are equally distributed:*

- *rights and welfare of mentally challenged and mentally differently able persons who are incapable of giving informed consent or those with behavioural disorders must be protected;*
- *Adequate justification is required for the involvement of subjects such as prisoners, students, subordinates, employees, service personnel etc. who have reduced autonomy as research subjects.*

1. Guidance synthesis. Medical research for and with older people in Europe: proposed ethical guidance for good clinical practice: ethical considerations

2013

KEYWORDS:

| Vuln- | Frag- | Frail- |
| --- | --- | --- |
| x |  | x |

*Vulnerability is a condition, which represents ‘Those who are relatively (or absolutely) incapable of protecting their own interests’ (CIOMS. 2002) but may also reflect some more subtle issues particular to the study population.*

*Assent from Older and Vulnerable Participants Where there may be doubt that the older patient has fully understood the nature, purpose and implications of involvement in a clinical trial, it will be useful to check this matter with a simple tool. Then if there is a failure to understand, their assent will not be sufficient to allow participation in that research, unless it is supplemented by the assent or consent of a proxy or the legal representative, as appropriate in that jurisdiction. Whenever older people are unable to consent, their assent should still be sought systematically using appropriate information, in addition to the consent of their legal or authorised representative. The consent/assent process and assessment of capacity to consent should always be performed in a supportive and caring environment with respect for patients’ dignity and rights.*

1. Guide for research ethics committee members

2010

KEYWORDS:

| Vuln- | Frag- | Frail- |
| --- | --- | --- |
| x |  |  |

*Particular attention must be paid to dependent and vulnerable, whose proposed participation in a research project must always be justified specifically. In general, potential research participants must be the least vulnerable necessary to achieve the goals of the research.*

*Some groups of people may be especially vulnerable to coercion – e.g. those deprived of liberty, military service personnel, or those who are vulnerable within a given society because of prevailing social hierarchy.*

*Whilst restriction of research in this group is still regarded as a measure of human rights protection in order to avoid misuse/abuse of such vulnerable people, prohibiting their participation completely may have negative consequences.*

1. Guide to Internet Research Ethics

2018

KEYWORDS:

| Vuln- | Frag- | Frail- |
| --- | --- | --- |
| x |  |  |

*Children and adolescents who participate in research are entitled to special protection. So are other weak and vulnerable groups, such as the elderly or patients.*

*This requires comprehensive precautions to ensure that no children or vulnerable persons are recruited into studies intended for adults who do have the capacity to consent. In some cases, when the information is especially sensitive and the informants are vulnerable, these problems of obtaining and securing consent may indicate that the researcher ought to abstain from studies of that particular online community.*

1. Guideline for Application to Conduct Clinical Trials in Liberia

2014

KEYWORDS:

| Vuln- | Frag- | Frail- |
| --- | --- | --- |
| x |  |  |

*Study participants - Individuals whose willingness to volunteer in a clinical trial may be unduly influenced by the expectation, whether justified or not, of benefits associated with participation, or of a retaliatory response from senior members of a hierarchy in case of refusal to participate. Examples are members of a group with a hierarchical structure, such a medical, pharmacy, dental and nursing students, subordinate hospital and laboratory personnel, employees of the pharmaceutical industry, members of the armed forces, and persons kept in detention. Other vulnerable study participants include patients with incurable diseases, persons in nursing homes, unemployed or impoverished persons, and patients in emergency situations, ethnic minority groups, homeless persons, nomads, refugees, minors, and those incapable of giving consent.*

1. Guideline for Good Clinical Practice (GCP) in Sierra Leone

2018

KEYWORDS:

| Vuln- | Frag- | Frail- |
| --- | --- | --- |
| x |  |  |

*Vulnerable Subjects/population- Individuals whose willingness to volunteer in a clinical trial may be unduly influenced by the expectation, whether justified or not, of benefits associated with participation, or of a retaliatory response from senior members of a hierarchy in case of refusal to participate. Examples are members of a group with a hierarchical structure, such a medical, pharmacy, dental and nursing students, subordinate hospital and laboratory personnel, employees of the pharmaceutical industry, members of the armed forces, and persons kept in detention. Other vulnerable study participants include patients with incurable diseases, persons in nursing homes, unemployed or impoverished persons, and patients in emergency situations, ethnic minority groups, homeless persons, nomads, refugees, minors, and those incapable of giving consent.*

*Steps must be taken to overcome these conditions, and to promote and protect the dignity, safety and welfare of participants. The vulnerability factors and steps that will be taken to offset these should be addressed in the study design and clearly outlined in the research protocol. It is imperative that sound study designs, and use of universally accepted ethical standards are applied in both vulnerable and non-vulnerable communities.*

*An IRB/IEC should safeguard the rights, safety, and well-being of all trial subjects. Special attention should be paid to trials that may include vulnerable subjects.*

*Because of their extreme vulnerability unconscious persons should be excluded from all but minimally invasive observational research.*

*Because of their extreme vulnerability, patients in emergency situations should be excluded from all but minimally invasive observational research.*

1. Guideline for Regulating the Conduct of Clinical Trials Using Medicines in Human Participants

2012

KEYWORDS:

| Vuln- | Frag- | Frail- |
| --- | --- | --- |
| x |  |  |

*Vulnerable Subjects Individuals whose willingness to volunteer in a clinical trial may be unduly influenced by the expectation, whether justified or not, of benefits associated with participation, or of a retaliatory response from senior members of a hierarchy in case of refusal to participate. Examples are members of a group with a hierarchical structure, such a medical, pharmacy, dental and nursing students, subordinate hospital and laboratory personnel, employees of the pharmaceutical industry, members of the armed forces, and persons kept in detention. Other vulnerable study participants include patients with incurable diseases, persons in nursing homes, unemployed or impoverished persons, and patients in emergency situations, ethnic minority groups, homeless persons, nomads, refugees, minors, and those incapable of giving consent.*

1. Guidelines for Conducting Clinical Trials of Medicines, Food Supplements, Vaccines, and Medical Devices in Sierra Leone

2014

KEYWORDS:

| Vuln- | Frag- | Frail- |
| --- | --- | --- |
| x |  |  |

*“Vulnerable population”*

*An individual whose willingness to volunteer in a clinical trial may be unduly influenced by the expectations, whether justified or not, of benefits associated with participation, or of a retaliatory response from senior members of a hierarchy in case of refusal to participate. Examples are pregnant women, cognitively impaired subjects, children and prisoners.*

*Clinical trials or study in vulnearable persons Special justification is required for inviting vulnerable individuals to serve as research subjects and, if they are selected, the means of protecting their rights and welfare must be strictly applied. Vulnerable persons are those who are relatively (or absolutely) incapable of protecting their own interests. More formally, they may have insufficient power, intelligence, education, resources, strength, or other needed attributes to protect their own interests.*

43. Guidelines for Good Clinical Practice E6 and Integrated Addendums E6(R3)

2025

KEYWORDS:

| Vuln- | Frag- | Frail- |
| --- | --- | --- |
| x |  |  |

*Vulnerable Participants*

*Individuals whose willingness to volunteer in a clinical trial may be unduly influenced by the expectation, whether justified or not, of benefits associated with participation or of a retaliatory response from senior members of a hierarchy in case of refusal to participate. Examples are members of a group with a hierarchical structure, such as medical, pharmacy, dental and nursing students; subordinate hospital and laboratory personnel; employees of the pharmaceutical industry; members of the armed forces; and persons kept in detention. Other vulnerable participants may include persons in nursing homes, unemployed or impoverished persons, patients in emergency situations, ethnic minority groups, homeless persons, nomads, refugees, minors and those incapable of giving consent.*

*The purpose of an IRB/IEC is to safeguard the rights, safety and well-being of all trial participants. Appropriate consideration should be given to trials that intend to recruit vulnerable participants.*

1. Guidelines for Including People with Disabilities in Research

2002

KEYWORDS:

| Vuln- | Frag- | Frail- |
| --- | --- | --- |
| x |  |  |

*People with mental health difficulties involuntarily held in institutions may be more vulnerable to coercion or undue influence due to the unequal power relationship between doctors and patients.*

*Freedman also suggests that people with learning disabilities may also be vulnerable to non-voluntary consent due to “their general lack of experience with decision-making, reliance upon family and staff, desire to please others, and potential susceptibility to undue influence or coercion from others (e.g. caregivers, therapists, researchers).”*

1. Guidelines for Phase I Clinical Trials

2018

KEYWORDS:

| Vuln- | Frag- | Frail- |
| --- | --- | --- |
| x |  |  |

*Vulnerable subjects Investigators must be wary of recruiting vulnerable trial subjects, such as the unemployed, or employees of the company or students of the institution that is sponsoring or carrying out the trial. Employees and students are, or may feel, vulnerable to pressure from someone who can influence their careers. Should such subjects decide to take part in the trial, they must be dealt with like other subjects in the trial, and not be allowed to let their normal work interfere with the trial. The investigator should forewarn employees, in a written agreement, of the possible implications of having their personal data processed at work by their colleagues.*

1. Guidelines for Research Among Children and Young People

2011

KEYWORDS:

| Vuln- | Frag- | Frail- |
| --- | --- | --- |
| x |  |  |

*In order to develop appropriate approaches to engage in research those CYP with particular needs or identified as ‘vulnerable’, it is particularly useful to consult with practitioners and gatekeepers who know them well. Where relevant, we also recommend briefly reviewing any recent research among similar groups of CYP to see how other researchers have engaged with them, and the challenges they faced. For example, it has been noted that young refugees and asylum seekers may be uncomfortable with the use of digital voice recorders in the research process because it can remind them of their experiences in detention or immigration centres.*

*For particularly vulnerable 16–18 year olds (for example if they have a learning disability), or if the research is on an exceptionally sensitive or troubling topic, you may also consider it appropriate to seek parental consent.*

1. Guidelines for Research Ethics in the Social Sciences, Law, and the Humanities

2016

KEYWORDS:

| Vuln- | Frag- | Frail- |
| --- | --- | --- |
| x |  |  |

*Disadvantaged and vulnerable groups have a particular need for protection. Specific attention may be required in research across cultures or on cultural heritage. Public offices and private organisations have a joint responsibility to ensure that their participation in research is in accordance with recognised norms of research ethics.*

*The research community has a social responsibility to gain experience with and develop knowledge about members of vulnerable groups. Historically, disadvantaged and vulnerable groups have often been subject to abuses of power and unethical research. Conducting research on weak and vulnerable groups simply because these groups are easily accessible is irresponsible. Researchers must always ensure that research is performed in accordance with recognised norms of research ethics concerning respect, protection, and justice, particularly when acquiring free and informed consent. The capacity of participants to consent should be evaluated based on individual competence, not on group characteristics. Members of disadvantaged and vulnerable groups may wish not to be subjects of research, for instance for fear of stigmatisation or other negative consequences. At the same time, excessive protection of weak and vulnerable groups is inappropriate. This might result in their perspectives being excluded in research, and society might not gain knowledge about important topics. When performing research on weak and vulnerable groups, researchers must avoid using classifications or terms that invite unreasonable generalisations, are defamatory and/or could lead to group stigmatisation. Researchers must be clear about their roles and responsibilities both in research and in dissemination.*

1. Guidelines on Ethics for Health Research in Tanzania

2009

KEYWORDS:

| Vuln- | Frag- | Frail- |
| --- | --- | --- |
| x |  |  |

*Vulnerable subjects in health research are individuals whose capacity and willingness to volunteer in a clinical trial or health research may be unduly influenced by the expectation(s), whether justified or not, of benefits associated with participation, or of a retaliatory response from senior members of a hierarchy in the case of refusal to participate (e.g. student, military, subordinate staff, persons with incurable diseases, unemployed, patients in emergency situations, refugees, minors, impoverished persons and those incapable of giving consent).*

*Care must therefore be taken to ensure that such vulnerability is not exploited*.

*Selection of subjects is equitable. In making this assessment IREC should take into account the purposes of the research and the setting in which the research will be conducted and should be particularly cognizant of the special problems of research involving vulnerable populations*

*Some or all of the subjects such as children, pregnant women, prisoners, handicapped or mentally disabled persons, or economically or educationally disadvantaged persons are likely to be vulnerable to coercion*

*Where relevant, the research protocol must describe the social contexts of a proposed research population, e.g. country or community that create conditions for possible exploitation or increased vulnerability among potential research subjects, and strategies that will be taken to overcome these conditions and protect the dignity, safely and welfare of the participants*

*Consent from vulnerable groups in the society There are circumstances where prospective candidates are unable to freely consent to research procedures for one reason or another. They include children, mentally handicapped, prisoners, detainees, refugees, severely ill or unconscious patients and the elderly. Research that can be carried out on subjects who can consent should not be carried out on individuals who have no capacity to understand, or freedom to refuse is limited. It has always been argued that it is unethical to include in a study individuals whose capacity to consent is limited. However, there are those who have argued that excluding the entire groups of such people from a study of the basis of limited capacity to consent is unethical, discriminatory, and it means there is failure in the system to seek measure to improve their conditions. It is however, generally accepted that carrying out research in groups of people with limited capacity to consent is not unethical as long as it is governed by strict safeguards including: (i) Critical review by IRECs or NREC; (ii) The research should not be contrary to the interests of the subject; (iii) The research should not pose risks to the subjects or pose minimal risk; (iv) The research must be impossible to carry out in subjects with capacity to consent; and (v) Must be designed to have maximum benefits to other in the same category as the subject.*

*Prisoners and people living in detention are vulnerable in the sense that they are vulnerable to suggestions especially where hope for freedom has been advanced.*

*However, it should be born in the mind that students are a vulnerable group especially where the training institution is carrying out the research*

*Some countries or communities often described as “developing” have been perceived as inappropriate participants for some phases of clinical trials researches due to perceived increased level of vulnerability to exploitation or harm.*

1. Guidelines on Ethics for Medical Research, Reproductive Biology and Genetic Research

2002

KEYWORDS:

| Vuln- | Frag- | Frail- |
| --- | --- | --- |
| x |  |  |

*Given the vulnerable population, the Editorial Committee's decision has been to emphasise the principle of autonomy - particularly from the perspective of 'non-exploitation' of research participants*

1. Guidelines on Regulating the Conduct of Clinical Trials in Human Participants

2016

KEYWORDS:

| Vuln- | Frag- | Frail- |
| --- | --- | --- |
| x |  |  |

*Vulnerable Subjects Individuals whose willingness to volunteer in a clinical trial may be unduly influenced by the expectation, whether justified or not, of benefits associated with participation, or of a retaliatory response from senior members of a hierarchy in case of refusal to participate. Examples are members of a group with a hierarchical structure, such as medical, pharmacy, dental, and nursing students, subordinate hospital and laboratory personnel, employees of the pharmaceutical industry, members of the armed forces, and persons kept in detention. Other vulnerable subjects include patients with incurable diseases, persons in nursing homes, unemployed or impoverished persons, patients in emergency situations, ethnic minority groups, homeless persons, nomads, refugees, minors, and those incapable of giving consent.*

1. Handbook for Good Clinical Research Practice (GCP): Guidance for Implementation

2005

KEYWORDS:

| Vuln- | Frag- | Frail- |
| --- | --- | --- |
| x |  |  |

*In general, all individuals, including healthy volunteers, who participate as research subjects should be viewed as intrinsically vulnerable. When some or all of the subjects, such as children, prisoners, pregnant women, handicapped or mentally disabled persons, or economically or educationally disadvantaged persons are likely to be more vulnerable to coercion or undue influence, additional safeguards should be included in the study to protect the rights and welfare of these subjects. These safeguards may include, but are not limited to: special justification to the ethical review committee that the research could not be carried out equally well with less vulnerable subjects; seeking permission of a legal guardian or other legally authorized representative when the prospective subject is otherwise substantially unable to give informed consent; including an impartial witness to attend the informed consent process if the subject or the subject’s legally authorized representative cannot read; and/or additional monitoring of the conduct of the study.*

*Subjects may also become more or less vulnerable throughout a study as circumstances about their health status and lives change.*

*“Vulnerable persons are those who are relatively (or absolutely) incapable of protecting their own interests. More formally, they may have insufficient power, intelligence, education, resources, strength, or other needed attributes to protect their own interests”. Examples of vulnerable persons include, but are not limited to: children, individuals with diminished mental capacity, prisoners, institutionalized persons (including orphans), patients in emergency situations, the economically disadvantaged, individuals who cannot give consent.*

*One special instance of injustice results from the involvement of vulnerable subjects. Certain groups, such as racial minorities, the economically disadvantaged, the very sick, and the institutionalized may continually be sought as research subjects, owing to their ready availability in settings where research is conducted. Given their dependent status and their frequently compromised capacity for free consent, they should be protected against the danger of being involved in research solely for administrative convenience, or because they are easy to manipulate as a result of their illness or socioeconomic condition.*

1. Implementing Regulations of the Law of Ethics of Research on Living Creatures

2022

KEYWORDS:

| Vuln- | Frag- | Frail- |
| --- | --- | --- |
| x |  |  |

*Vulnerable groups: groups of individuals in need of additional protection due to their lack of legal capacity, their questionable or diminished capacity or their lack of freedom to choose.*

*The local committee shall ensure especially that minors, legally incompetent or disabled persons, or any other persons from (vulnerable groups) are never abused under any circumstance.*

*If the research subject proposed for discussion by the local committee is related to any category of the (vulnerable groups), the committee chairman shall invite a specialist with experience in this field to take part in discussing said subject.*

*Before consenting to conduct a research project, the local committee shall ensure that research subjects have been selected based on their understanding of research objectives, place, time and method of conducting research, with special additional attention in the cases in which the participation of persons requiring additional protection is requested, such as vulnerable groups.*

1. Institutional Review Board (IRB) Policies and Procedures Handbook

2020

KEYWORDS:

| Vuln- | Frag- | Frail- |
| --- | --- | --- |
| x |  |  |

*The Board shall safeguard the dignity, rights, safety, and well-being of all study participants/subjects and communities, paying special attention to studies that involve vulnerable participants (i.e., children, pregnant women, prisoners, etc.).*

*A determination that when some or all subjects are likely to be vulnerable to coercion or undue influence (such as children, prisoners, individuals with impaired decision-making capacity, or economically or educationally disadvantaged persons), additional safeguards have been included in the study to protect the rights and welfare of subjects.*

*Due to the vulnerability of prisoners, research involving prisoners should be reviewed by the full convened IRB.*

1. International Code of Marketing & Social Research Practices

2016

KEYWORDS:

| Vuln- | Frag- | Frail- |
| --- | --- | --- |
| x |  |  |

*Vulnerable people means individuals who may have limited capacity to make voluntary and informed decisions, including those with cognitive impairments or communication disabilities*.

*Children, young people and other vulnerable individuals*

- *Researchers must obtain the consent of the parent or responsible adult when collecting personal data from children or anyone for whom a legal guardian has been appointed*
- *Researchers must take special care when considering whether to involve children and young people in research. The questions asked must take into account their age and level of maturity.*
- *When working with other vulnerable individuals, researchers must ensure that such individuals are capable of making informed decisions and are not unduly pressured to cooperate with a research request*

1. International Ethical Guidelines for Research Involving Humans (CIOMS)

2016

KEYWORDS:

| Vuln- | Frag- | Frail- |
| --- | --- | --- |
| x | x | x |

*According to the Declaration of Helsinki, vulnerable groups and individuals “may have an increased likelihood of being wronged or of incurring additional harm.” This implies that vulnerability involves judgments about both the probability and degree of physical, psychological, or social harm, as well as a greater susceptibility to deception or having confidentiality breached. It is important to recognize that vulnerability involves not only the ability to provide initial consent to participate in research, but also aspects of the ongoing participation in research studies. In some cases, persons are vulnerable because they are relatively (or absolutely) incapable of protecting their own interests. This may occur when persons have relative or absolute impairments in decisional capacity, education, resources, strength, or other attributes needed to protect their own interests. In other cases, persons can also be vulnerable because some feature of the circumstances (temporary or permanent) in which they live makes it less likely that others will be vigilant about, or sensitive to, their interests. This may happen when people are marginalized, stigmatized, or face social exclusion or prejudice that increases the likelihood that others place their interests at risk, whether intentionally or unintentionally. Although research ethics committees can require special protections only for potential participants collectively for a particular project, researchers and others involved in research must take into account factors that render individual participants vulnerable and take appropriate steps to mitigate those factors. A traditional approach to vulnerability in research has been to label entire classes of individuals as vulnerable. The account of vulnerability in this Guideline seeks to avoid considering members of entire classes of individuals as vulnerable. However, it is useful to look at the specific characteristics that may render individuals vulnerable, as this can aid in identifying the special protections needed for persons who may have an increased likelihood of being wronged or of incurring additional harm as participants in research. Different characteristics may also co-exist, making some individuals more vulnerable than others. This is highly dependent on the context. For example, persons who are illiterate, marginalized by virtue of their social status or behaviour, or living in an authoritarian environment, may have multiple factors that make them vulnerable. Some characteristics can make it reasonable to assume that certain individuals are vulnerable, for example: Capacity to consent. One widely accepted criterion of vulnerability is limited capacity to consent or decline to consent to research participation.*

*Individuals in hierarchical relationships: the characteristic of vulnerability in this case is the possibility of diminished voluntariness of the consent of potential participants who are in a subordinate relationship. Examples are medical and nursing students, subordinate hospital and laboratory personnel, workers in settings where research studies are conducted, and members of the armed forces or police. Their agreement to volunteer may be unduly influenced, whether justified or not, by the expectation of preferential treatment if they agree to participate in the study or by fear of disapproval or retaliation if they refuse. The research protocol must include a description of provisions to protect such individuals from being conscripted into research.*

*Institutionalized persons: residents of nursing homes, mental institutions, and prisons are often considered vulnerable because in a confined setting they have few options and are denied certain freedoms that non-institutionalized persons enjoy. Also, they may be in a dependent relationship with caregivers or guardians. One protection for institutionalized individuals is the appointment of an advocate of some sort to the research ethics committee when such proposals are under review.*

*Women: although women in general must not be considered vulnerable, specific circumstances in which women could be vulnerable in research include: studies with female or transsexual sex workers; research on sexual and intimate partner violence; studies with trafficked women, refugees and asylum seekers; studies of abortion in jurisdictions where abortion is illegal; and research with women who live in a cultural context where they are not permitted to consent on their own behalf for participation in research, but require permission from a spouse or male relative. When women in such situations are potential participants in research, researchers need to exercise special care.*

*Pregnant women: pregnant women must not be considered vulnerable simply because they are pregnant. Specific circumstances, such as risks to the fetus, may require special protections.*

*Other potentially vulnerable individuals: among members of groups that have traditionally been considered vulnerable, the following are frequently mentioned: people receiving welfare benefits or social assistance and other poor people and the unemployed; people who perceive participation as the only means of accessing medical care; some ethnic and racial minorities; homeless persons, nomads, refugees or displaced persons; people living with disabilities; people with incurable or stigmatized conditions or diseases; people faced with physical frailty, for example because of age and co-morbidities, individuals who are politically powerless; and members of communities unfamiliar with modern medical concepts. Furthermore, in some contexts vulnerability might be related to gender, sexuality and age. To the extent that these and other people have one or more of the characteristics discussed above, research ethics committees must review the need for special protection of their rights and welfare, and include such protections when necessary. However, researchers and research ethics committees must avoid making judgments regarding the exclusion of such groups based on stereotypes. One proposed mechanism that can be used to avoid stereotyping is consultation with relevant stakeholders, where feasible, before, during and after the conduct of the research. Special protections for these groups can include allowing no more than minimal risks for procedures that offer no potential individual benefits for participants; supplementing the participant’s agreement by the permission of family members, legal guardians, or other appropriate representatives; or requiring that the research be carried out only when it is targeted at conditions that affect these groups. Research ethics committees need to be sensitive to not overly excluding people and allow them to participate by requiring that special protections be put in place.*

*Group vulnerability: despite the importance of avoiding classification of entire groups as inherently vulnerable, circumstances exist that require research ethics committees to pay special attention to research involving certain groups. In some resource-limited countries or communities, lack of access to medical care, membership in ethnic and racial minorities, or other disadvantaged or marginalized groups can be factors that constitute vulnerability. As is true of the vulnerability of individuals, the judgment that groups are vulnerable is context dependent and requires empirical evidence to document the need for special protections.*

*Vulnerability of women: Some women become vulnerable in research because of heightened psychological, social, physical, or legal risks. Examples include surveys and interviews regarding intimate partner violence and rape; social and behavioural research involving sex workers or women who inject drugs; and studies that solicit information about sexual behaviour. When the research involves household surveys or interviews, researchers must take special care to ensure that the women are interviewed in a private place without the possibility of intrusion by other family members. In such studies, women must be given the option of conducting the interview in a setting of their choosing outside the home. Breach of confidentiality in these types of research could result in serious harms to women, even if the only information disclosed is their participation in the research. In studies involving women who have experienced gender-based violence, participation in interviews may cause emotional distress. Researchers must be prepared with referrals for psychological counselling if the need arises.*

*Fair selection of participants is essential. Especially in dire emergencies, well-off and well-connected patients must not be further privileged (for example, community leaders). Moreover, the exclusion of especially vulnerable populations must be justified.*

*When a proposed study involves vulnerable individuals or groups, as may be the case in research involving prisoners or illiterate persons, representatives of relevant advocacy groups should be invited to meetings where such protocols will be reviewed.*

1. Malaysian Phase I Clinical Trial Guidelines

2012

KEYWORDS:

| Vuln- | Frag- | Frail- |
| --- | --- | --- |
| x |  |  |

*Vulnerable subjects Investigators must be wary of recruiting vulnerable trial subjects, such as the homeless, or employees of the company or students of the institution that is sponsoring or carrying out the trial. Employees and students are, or may feel, vulnerable to pressure from someone who can influence their careers. Should such subjects decide to take part in the trial, they must be dealt with like other subjects in the trial, and not be allowed to let their normal work interfere with the trial. The investigator should forewarn employees, in a written agreement, of the possible implications of having their personal data processed at work by their colleagues.*

57. Medical Products in Human Medicine Act

2020

KEYWORDS:

| Vuln- | Frag- | Frail- |
| --- | --- | --- |
| x |  |  |

*"Vulnerable patient groups" shall mean persons whose wish to participate in a clinical test can be affected by the anticipation of benefits or affected by eventual penalty on the part of higher officials in the hierarchical structure associated with the person’s participation or refusal to participate in the clinical test. Examples of such group in the hierarchical structures shall be students in medicine, pharmacy, dental medicine or nursing, laboratory personnel, employees in the pharmaceutical industry, members of the armed forces or persons deprived of freedom. Other vulnerable groups shall be patients with incurable diseases, persons in hospices, unemployed and beggars, patients in critical conditions, waifs and strays, under-aged and minors and persons who are unable to give consent.*

58. Medical Research Involving Children

2004

KEYWORDS:

| Vuln- | Frag- | Frail- |
| --- | --- | --- |
| x |  |  |

*In the past, the concern to protect children from the potential harms of research may have denied them potential benefits. To ensure that this vulnerable group are not exploited, the General Medical Council advises that it is important to assess carefully the potential benefits and harm to children at all stages of any research. As the benefits of research are not predictable, the researcher must be satisfied that the research is not contrary to the child participant's interests.*

*The ethics committee must be satisfied that dependent persons and vulnerable groups will not be subjected to undue influence.*

59. National Ethical Guidelines for Biomedical and Health Research Involving Human Participants

2017

KEYWORDS:

| Vuln- | Frag- | Frail- |
| --- | --- | --- |
| x | x |  |

*Vulnerable individuals/groups should not be included in research to solely benefit others who are better-off than themselves.*

*Vulnerable groups and individuals may have an increased likelihood of incurring additional harm as they may be relatively (or absolutely) incapable of protecting their own interests. Characteristics that make individuals vulnerable are legal status – children; clinical conditions – cognitive impairment, unconsciousness; or situational conditions – including but not limited to being economically or socially disadvantaged, (for example, certain ethnic or religious groups, individuals/communities which have hierarchical relationships, institutionalized persons, humanitarian emergencies, language barriers and cultural differences). In general, such participants should be included in research only when the research is directly answering the health needs or requirements of the group. On the other hand, vulnerable populations also have an equal right to be included in research so that benefits accruing from the research apply to them as well. This needs careful consideration by researchers as well as the EC. The EC should determine vulnerability and ensure that additional safeguards and monitoring mechanisms are established. It should also advise the researcher in this regard.*

*Careful consideration should be given to protecting the dignity, rights, safety and well-being of the participants in cases where the social contexts of the proposed research can create foreseeable conditions for their exploitation or increase their vulnerability to harm.*

*For research that involves higher risk or vulnerable participants or if there is any other reason for concern, the EC at the time of initial review or continuing review can suggest that routine monitoring may be conducted at more frequent intervals. Researchers must justify the inclusion of a vulnerable population in the research. Vulnerable persons may require repeated education/information about the research, benefits, risks and alternatives, if any.*

*Vulnerable populations have an equal right to be included in research so that benefits accruing from the research apply to them as well. If any vulnerable group is to be solely recruited then the research should answer the health needs of the group.*

*Children are individuals who have not attained the legal age of consent (up to 18 years). At younger ages, children are considered vulnerable because their autonomy is compromised as they do not have the cognitive ability to fully understand the minute details of the study and make decisions. At older ages, although they may attain the cognitive ability to understand the research, they still lack legal capacity to consent. Therefore, the decision regarding participation and withdrawal of a child in research must be taken by the parents/ LAR in the best interests of their child/ward.*

*Terminally ill patients or patients who are in search of new interventions having exhausted all available therapies are vulnerable as they are ready to give consent for any intervention that can give them a ray of hope.*

*Other vulnerable groups include the economically and socially disadvantaged, homeless, refugees, migrants, persons or populations in conflict zones, riot areas or disaster situations. Additional precautions should be taken to avoid exploitation/retaliation/ reward/credits and other inducements when such individuals are to be recruited as research participants.*

*In some circumstances participants become more vulnerable in research because of heightened psychological, social, physical or legal risks. Breach of confidentiality in these types of research may cause serious harm to vulnerable participants. It is important to protect study participants from potential future risks and harm by establishing culturally sensitive and context specific safeguards.*

*Vulnerability in research pertains to individuals who are relatively or absolutely incapable of protecting their own interests because of personal disability, environmental burdens or social injustice, lack of power, understanding or ability to communicate or are in a situation that prevents them from doing so.*

60. National Ethical Guidelines for Biomedical Research Involving Children

2017

KEYWORDS:

| Vuln- | Frag- | Frail- |
| --- | --- | --- |
| x | x |  |

*Some research populations (such as children) are particularly vulnerable and have increased likelihood of incurring additional and greater harm.*

*Vulnerable means an individual or group of people who are not in a position to make autonomous decisions regarding participation in research, for example, children, students, prisoners, mentally challenged individuals and others. This set of participants cannot give or refuse consent for themselves and they may be at risk of coercion or undue influence. All vulnerable groups need specifically considered protection. In vulnerable populations, biomedical research is justified only if it is based on the health priorities of that population.*

*Overuse of these vulnerable groups is a special concern when they are unlikely to benefit from the knowledge gained from research.*

*Cognitively impaired children or children with developmental disorders form one of the most vulnerable populations. In fact, their parents are also vulnerable and there is a high likelihood of therapeutic misconception. The potential benefits and risks must be explained carefully to parents so that they understand the proposed research.*

*Neonates represent the most vulnerable group within the paediatric population. Study protocols in this population should take into account this, and the potential long-term effects of interventions, including developmental effects. ECs’ reviewing any research proposed in neonates should have an advisory member with expertise in neonatal research/care. ECs’ should carefully scrutinize all research proposed in neonates for potential risks. Risks if any should be carefully weighed against possible benefits in this fragile population.*

61. National Ethical Guidelines for Health and Health-Related Research

2017

KEYWORDS:

| Vuln- | Frag- | Frail- |
| --- | --- | --- |
| x |  | x |

*Vulnerable participants shall require special protection because of certain characteristics or situations that render them as such. Vulnerable participants are those who are relatively or absolutely incapable of deciding for themselves whether or not to participate in a study for reasons such as physical and mental disabilities, poverty, asymmetric power relations, and marginalization, among others and who are at greater risk for some harms. Vulnerable groups shall not be included in research unless such research: is necessary to promote the welfare of the population represented and cannot be performed on non-vulnerable persons or groups.*

*Soliciting the participation of prisoners and other institutionalized persons, or of indigent groups. The marginalized status of these samples, and their restricted autonomy, make them vulnerable to coercion. Researchers shall take more care to uphold their autonomous right to decide to participate in a study.*

*Minors are considered as belonging to a vulnerable population*

*Persons with disabilities are classified as vulnerable participants, and the informed consent process shall ensure freedom from manipulation and coercion; with special consideration to this population’s special needs*

*The vulnerability of terminally-ill patients, or those with chronic disease without effective treatment, shall be seriously addressed. Such patients shall be accorded special protection to prevent exploitation and abuse.*

*Vulnerability – the state of being relatively or absolutely incapable of deciding for oneself whether or not to participate in a study, for reasons such as physical and mental disabilities, poverty, asymmetric power relations, and marginalization, among others.*

*Vulnerable Persons or Groups – individuals or groups which require special protection because of certain characteristics or situations that render them relatively or absolutely incapable of deciding for themselves whether or not to participate in a study.*

62. National Guidelines for Ethical Conduct of Research Involving Human Subjects

2008

KEYWORDS:

| Vuln- | Frag- | Frail- |
| --- | --- | --- |
| x |  |  |

*Protection of persons with impaired or diminished autonomy, which requires that those who are dependent or vulnerable be afforded full security against harm or abuse.*

*Members of vulnerable groups have the same entitlement to access to the benefits of investigational interventions that show promise of therapeutic benefit as non vulnerable groups particularly when no superior or equivalent approaches to therapy are available.*

*Special justification is required for inviting vulnerable individuals to serve as research subjects and, if they are selected, the means of protecting their rights and welfare must be strictly applied. Individuals conventionally considered vulnerable are those with limited capacity or freedom to consent or to decline to consent. They include children, and persons who because of mental or behavioural disorders are incapable of giving informed consent.*

*Research involving vulnerable groups include: children, women, pregnant women.*

63. National Guidelines for Ethics Committees Reviewing Biomedical and Health Research During Covid-19 Pandemic

2020

KEYWORDS:

| Vuln- | Frag- | Frail- |
| --- | --- | --- |
| x |  |  |

*Vulnerable Persons are individuals belonging to certain groups of persons who are relatively or absolutely incapable of protecting their own interests such as: COVID-19 patients may be additionally vulnerable of being stigmatized due to the contagious nature of the disease. Socially, economically or politically disadvantaged individuals such as the stranded migrant workers who are susceptible to being exploited; Incapable of making a voluntary informed decision or whose autonomy is compromised temporarily or permanently; Able to give consent, but voluntariness/understanding compromised due to their situation; Unduly influenced either by the expectation of benefits or fear of retaliation in case of refusal to participate which may lead them to give consent; Terminally ill patients ready to consent in search of new interventions.*

*Additional safeguards are required for research to address the needs of participants and justify inclusion of vulnerable persons.*

64. National Guidelines for Research Involving Humans as Research Participants

2014

KEYWORDS:

| Vuln- | Frag- | Frail- |
| --- | --- | --- |
| x |  |  |

*Individuals and groups conventionally considered vulnerable are those with limited capacity or freedom to consent or decline consent. These include, but are not limited to, children, mature and emancipated minors, street children, prisoners, the homeless, refugees, adults staying on the street, internally displaced persons, substance abusers, handicapped (mentally and physically), armed forces, terminally ill and pregnant women.*

*Vulnerable groups and individuals need special considerations to ensure their protection. Researchers, whose research involves vulnerable groups and individuals, shall specify how they will address particular vulnerabilities.*

*Certain communities may also be vulnerable. Characteristics that constitute vulnerability in such communities include one or more of the following: Limited economic empowerment; Conflict and post-conflict situations; Inadequate protection of human rights; Discrimination on the basis of health status; Limited availability of health care and treatment options; Communities in acute disaster and disease epidemics.*

*Research can only be conducted in this group and individuals if the objectives of the research project cannot be addressed using non-vulnerable groups and individuals. RECs may co-opt a person knowledgeable about and has experience working with the vulnerable group and individuals.*

*Vulnerability refers to a substantial incapacity to protect one’s own interests owing to such impediments as lack of capability to give informed consent, lack of alternative means of obtaining medical care or other expensive necessities, or being a junior or subordinate member of a hierarchical group.*

65. National Health Research Ethics Review Guideline, Fourth Edition

2014

KEYWORDS:

| Vuln- | Frag- | Frail- |
| --- | --- | --- |
| x |  |  |

*Justice demands equitable selection of participants, i.e., avoiding populations that may be unfairly coerced into participating, including but not limited to, prisoners, pregnant women, people with mental and physical disabilities, immigrants, refugees, ethnic minorities, marginalized groups and institutionalized persons, including children*. *There must be a justification for inclusion of these vulnerable groups in the research. There should be no disproportionate use of vulnerable populations. The same recruitment approach should be used in all populations.*

*If an IRB regularly reviews research that involves vulnerable populations, such as children, prisoners, pregnant women, men/women in uniform or persons with disabilities, marginalized group, refuges, minorities, the IRB shall involve or co-opt one or more individuals who are knowledgeable about and experienced in working with these research participants.*

*Vulnerable population involvement requires further explanation to justify that without these vulnerable populations involvement there is no other way of obtaining the relevant data.*

*Vulnerable populations are those segments of the population whose capacity to safeguard their welfare, demand their rights and satisfy their interests, is compromised. Because of these limitations, they cannot provide or refuse consent. Vulnerable populations are particularly subject to undue influence, manipulation, coercion, and intimidation.*

*Vulnerable population-A group identified by one or more common characteristics associated with reduced capacity to offer free and informed consent.*

66. National Statement on Ethical Conduct in Human Research

2018

KEYWORDS:

| Vuln- | Frag- | Frail- |
| --- | --- | --- |
| x |  |  |

*Young people of developing maturity, who are able to understand the relevant information but whose relative immaturity means that they remain vulnerable. The consent of these young people is required, but is not sufficient to authorise research.*

*A review body may also approve research to which only the young person consents if it is satisfied that he or she is mature enough to understand the relevant information and to give consent, although vulnerable because of relative immaturity in other respects.*

67. Nigerian Code of Health Research Ethics

2007

KEYWORDS:

| Vuln- | Frag- | Frail- |
| --- | --- | --- |
| x |  |  |

*If HREC wishes to review research that involves vulnerable participants, such as children, prisoners, pregnant women, physically and psychologically disabled persons, the HREC shall co-opt one or more individuals knowledgeable about and experienced in working with these participants for the review process.*

*Children, pregnant women, socially, culturally, economically, politically, educationally, physically and psychologically disadvantaged groups, groups with constrained autonomy and other vulnerable populations should not be excluded from research without explicit reasons for doing so; particularly from studies that can advance their health and well-being. However specific safeguards should be included to protect the vulnerable, appropriate to degree of risk.*

68. Note for guidance on Good Clinical Practice (CPMP/ICH-135/95)

2000

KEYWORDS:

| Vuln- | Frag- | Frail- |
| --- | --- | --- |
| x |  |  |

*Vulnerable Subjects*

*Individuals whose willingness to volunteer in a clinical trial may be unduly influenced by the expectation, whether justified or not, of benefits associated with participation, or of a retaliatory response from senior members of a hierarchy in case of refusal to participate. Examples are members of a group with a hierarchical structure, such as medical, pharmacy, dental, and nursing students, subordinate hospital and laboratory personnel, employees of the pharmaceutical industry, members of the armed forces, and persons kept in detention. Other vulnerable subjects include patients with incurable diseases, persons in nursing homes, unemployed or impoverished persons, patients in emergency situations, ethnic minority groups, homeless persons, nomads, refugees, minors, and those incapable of giving consent.*

69. Personal Information in Biomedical Research

2007

KEYWORDS:

| Vuln- | Frag- | Frail- |
| --- | --- | --- |
| x |  |  |

*Vulnerability may be thought to exist if one’s ability to give voluntary consent is compromised or if one would be at heightened risk of adverse consequences from the research. Three common categories of vulnerable person are children and adolescents; the mentally impaired;persons in dependent relationships.*

*When vulnerable persons are involved in research, they are entitled, as a general rule, to the same considerations of privacy and confidentiality protection as any other research participants, and this principle needs to be kept in mind in the conduct of the research.*

*Vulnerable persons raise particular ethical issues in research, especially where consent is concerned. This is because their interests must be considered, if necessary by proxy, and their participation sought only when other research participants are unavailable or unsuitable.*

*Emphasizing the twin responsibilities of researchers both to use and protect should help to defend vulnerable persons against disrespect, abuse and even exploitation.*

70. Policy for the Protection and Welfare of Vulnerable Adults and the Management of Allegations of Abuse

2022

KEYWORDS:

| Vuln- | Frag- | Frail- |
| --- | --- | --- |
| x |  |  |

*Vulnerable Person is an adult who is restricted in capacity to guard himself/herself against harm or exploitation or to report such harm or exploitation. This may arise as a result of physical or intellectual impairment and risk of abuse may be influenced by both context and individual circumstances. Avista is committed to the safeguarding of vulnerable persons from abuse. It acknowledges that all adults have the right to be safe and to live a life free from abuse.*

*All staff should be aware that safeguarding vulnerable persons is an essential part of their duty. Staff must be alert to the fact that abuse can occur in a range of settings and, therefore, must make themselves aware of the signs of abuse and the appropriate procedures to report such concerns or allegations of abuse.*

*Vulnerable person does not or is unable to consent to an activity or other barriers to consent exist, for example, where the person may be experiencing intimidation or coercion. For a valid consent to be given, consent must be full, free and informed. It is important that a vulnerable person is supported in making his/her own decisions about how he/she wishes to deal with concerns or complaints. The vulnerable person should be assured that his/her wishes concerning a complaint will only be overridden if it is considered essential for his/her own safety or the safety of others or arising from legal responsibilities.*

71. Policy Statement Regarding Enrollment of Children in Research in Nigeria

2016

KEYWORDS:

| Vuln- | Frag- | Frail- |
| --- | --- | --- |
| x |  |  |

*Children are considered a vulnerable group in research because their intellectual and emotional capacities are limited and therefore, they are considered legally ‘incompetent’ to give valid informed consent.*

*As a vulnerable group, it is standard best practice in research ethics that children are provided additional protection to minimize or eliminate any potential for their vulnerability to be unduly exploited.*

72. Qualitative methods in end-of-life research: Recommendations to enhance the protection of human subjects

2003

KEYWORDS:

| Vuln- | Frag- | Frail- |
| --- | --- | --- |
| x |  |  |

*Patients near the end of life should not be defined as “vulnerable” or in need of special protections simply because of their closeness to death. Dying patients should not be excluded from research simply because they are near the end of life. Similarly, bereaved family members should not be defined a priori as vulnerable.*

73. Recommendation (99) 4 on principles concerning the legal protection of incapable adults

1999

KEYWORDS:

| Vuln- | Frag- | Frail- |
| --- | --- | --- |
| x |  |  |

*Medical developments in the treatment of mental illness have also meant that a large number of patients who would formerly have required institutional care can now live in the community. Some such people may be vulnerable and in need of measures of protection.*

*There may be people – sometimes called "vulnerable adults" – who can make decisions but who are unable, because of some mental or physical disorder, to understand or to express them or act upon them. For such people decision-making is possible but effective decision-making is not. One example of a person who may be able to make decisions, but who may be as vulnerable and as in need of protection by representation or assistance as a person who cannot, would be a person who is totally unable to communicate or to express decisions.*

74. Regulation (EU) 2017/745 of the European Parliament and of the Council of 5 April 2017 on medical devices

2017

KEYWORDS:

| Vuln- | Frag- | Frail- |
| --- | --- | --- |
| x |  |  |

*Argumentation takes into account if the intended use of such devices includes treatment of children or treatment of pregnant or breastfeeding women or treatment of other patient groups considered particularly vulnerable.*

*Information on vulnerable subjects involved such as children, pregnant women, immuno-compromised or, elderly subjects*

75. Regulation No. 536/2014 of the European Parliament and of the Council on Clinical Trials on Medicinal Products for Human Use, Repealing Directive 2001/20/EC

2014

KEYWORDS:

| Vuln- | Frag- | Frail- |
| --- | --- | --- |
| x |  | x |

*In order to improve treatments available for vulnerable groups such as frail or older people, people suffering from multiple chronic conditions, and people affected by mental health disorders, medicinal products which are likely to be of significant clinical value should be fully and appropriately studied for their effects in these specific groups, including as regards requirements related to their specific characteristics and the protection of the health and well-being of subjects belonging to these groups.*

*Specific considerations for vulnerable populations*

*Where the subjects are minors, specific consideration shall be given to the assessment of the application for authorisation of a clinical trial on the basis of paediatric expertise or after taking advice on clinical, ethical and psychosocial problems in the field of paediatrics.*

*Where the subjects are incapacitated subjects, specific consideration shall be given to the assessment of the application for authorisation of a clinical trial on the basis of expertise in the relevant disease and the patient population concerned or after taking advice on clinical, ethical and psychosocial questions in the field of the relevant disease and the patient population concerned.*

*Where the subjects are pregnant or breastfeeding women, specific consideration shall be given to the assessment of the application for authorisation of a clinical trial on the basis of expertise in the relevant condition and the population represented by the subject concerned.*

76. Regulation Relating to Research with Human Participants No. R719

2014

KEYWORDS:

| Vuln- | Frag- | Frail- |
| --- | --- | --- |
| x |  |  |

*"Vulnerable persons" means those persons at increased risk of research-related harm, or who are limited in their freedom to make choices, or relatively incapable of protecting their own interests.*

*Research with vulnerable persons must involve vulnerable persons only when non-vulnerable persons are not appropriate for inclusion, not systematically avoid inclusion of vulnerable participants because to do so is unfairly discriminatory and vulnerable persons are potential beneficiaries of relevant research, be responsive to the health needs and the priorities of vulnerable persons and receive special attention in ethical review to ensure that research-related risks are assessed and minimized and that appropriate consent procedures are followed.*

77. Research Consent for Cognitively Impaired Adults. Recommendations for Institutional Review Boards and Investigators

2004

KEYWORDS:

| Vuln- | Frag- | Frail- |
| --- | --- | --- |
| x |  |  |

*Adults with cognitive impairment are considered a vulnerable population.*

*IRBs should include in their application forms a “check-off box” in the section on “vulnerable populations” where investigators are asked to address this issue.*

78. Research Ethics Framework (REF)

2005

KEYWORDS:

| Vuln- | Frag- | Frail- |
| --- | --- | --- |
| x |  |  |

*The following research would normally be considered as involving more than minimal risk: research involving vulnerable groups – for example, children and young people, those with a learning disability or cognitive impairment, or individuals in a dependent or unequal relationship.*

*In certain cases, research that involves vulnerable people may require CRB Disclosures. The CRB offers organisations a means to check the background of researchers to ensure that they do not have a history that would make them unsuitable for work involving children and vulnerable adults.*

*In cases where research involves vulnerable groups such as children, older persons or adults with learning difficulties, every effort should be made to secure their informed consent*

79. Research Ethics Policy and Procedures

2011

KEYWORDS:

| Vuln- | Frag- | Frail- |
| --- | --- | --- |
| x |  |  |

*Research Ethics Committees must make every reasonable effort to consider and protect the unique needs and rights of vulnerable groups, including, among others, prisoners, persons with disability, persons with reduced capacity for consent, and pregnant women. When considering projects involving vulnerable groups, Committees should make every effort to consult with advocates or members of the relevant group to inform the deliberations.*

80. Research Governance Framework

2008

KEYWORDS:

| Vuln- | Frag- | Frail- |
| --- | --- | --- |
| x |  |  |

*Persons with impaired or diminished autonomy be respected and protected: that is, people who are dependent or vulnerable, with less capacity to make personal choices, have their human condition respected equally to others’ with full capacity, and that additional protections be in place to safeguard them against harm or abuse; these include children, prisoners, people with cognitive disabilities, and people with severe illnesses.*

*Therefore, risk to vulnerable populations may be justified when research is intended to develop knowledge with the prospect of delivering health-related benefits for that particular population.*

*Informed consent of vulnerable populations: when a prospective subject is not capable of informed consent, satisfactory assurance that permission will be obtained from a duly authorized person, or, in the case of a child who is sufficiently mature to understand the implications of informed consent but has not reached the legal age of consent, that knowing agreement, or assent, will be obtained, as well as the permission of a parent, or a legal guardian or other duly authorized representative.*

81. Resolution CNS No. 466/2012 on Guidelines and Rules for Research Involving humans Subjects

2012

KEYWORDS:

| Vuln- | Frag- | Frail- |
| --- | --- | --- |
| x |  |  |

*Vulnerability – the condition of individuals or groups that, for any reason whatsoever, have their self-judgment capability reduced or disabled, or by any means they are prevented to resist to the opposition, especially when it comes to the informed consent form.*

*Individuals or vulnerable groups shall not participate in the research when the expected information may be obtained with participants fully capable, unless the research may bring benefits to the individuals or vulnerable groups.*

82. South African Good Clinical Practice: Clinical Trial Guidelines

2020

KEYWORDS:

| Vuln- | Frag- | Frail- |
| --- | --- | --- |
| x |  |  |

*External circumstances like low levels of literacy and formal education, advanced age, young age, personal and socio-economic circumstances, including significant poverty and poor access to health care, may increase vulnerability of South Africans. Certain groups of persons are vulnerable due to diminished health, loss of liberty or other health-related personal circumstances, including adults with diminished decisional capacity, persons with mental illness, mental disability, or persons who have substance abuse problems, persons in dependent relationships, incarcerated offenders and persons highly dependent on medical care.*

*Vulnerable persons should not be recruited merely because they are conveniently accessible if the research could be conducted with less vulnerable participants. The research for which vulnerable persons are to be recruited should be relevant to the health needs and priorities of their communities or groupings. RECs may impose additional measures to protect the welfare of vulnerable participants. For example, they may impose additional protective measures for the informed consent process, or require increased monitoring and interim reporting on the participants’ welfare.*

*Minors (younger than 18 years) are regarded as vulnerable persons due to their lack of legal capacity. Minors should participate in research only where their participation is indispensable to the research, i.e. the research cannot deliver the desired outcomes if adult participants were to be used instead.*

*Vulnerable persons include women, older persons, children and persons with disabilities*

*Vulnerable Participants Individuals whose willingness to volunteer in a clinical trial may be unduly influenced by the expectation, whether justified or not, of benefits associated with participation, or of a retaliatory response from senior members of a hierarchy in case of refusal to participate. Examples are members of a group with a hierarchical structure, such as medical, pharmacy, dental, and nursing students, subordinate hospital and laboratory personnel, employees of the pharmaceutical industry, members of the armed forces, and persons kept in detention. Other vulnerable subjects include patients with incurable diseases, persons in nursing homes, unemployed or impoverished persons, patients in emergency situations, ethnic minority groups, homeless persons, nomads, refugees, minors, and those incapable of giving consent.*

83. Standards and Operational Guidance for Ethics Review of Health-Related Research with Human Participants

2011

KEYWORDS:

| Vuln- | Frag- | Frail- |
| --- | --- | --- |
| x |  |  |

*Vulnerable persons are those who are relatively (or absolutely) incapable of protecting their own interests. More formally, they may have insufficient power, intelligence, education, resources, strength, or other needed attributes to protect their own interests.*

*Individuals whose willingness to volunteer in a clinical trial may be unduly influenced by the expectation, whether justified or not, of benefits associated with participation, or of a retaliatory response from senior members of a hierarchy in case of refusal to participate. Examples are members of a group with a hierarchical structure, such as medical, pharmacy, dental, and nursing students, subordinate hospital and laboratory personnel, employees of the pharmaceutical industry, members of the armed forces, and persons kept in detention. Other vulnerable subjects include patients with incurable diseases, persons in nursing homes, unemployed or impoverished persons, patients in emergency situations, ethnic minority groups, homeless persons, nomads, refugees, minors, and those incapable of giving consent.* *This list may not be exhaustive as there may be circumstances in which other groups are considered vulnerable, women for example, in an orthodox patriarchical society.*

84. The ethics of research related healthcare in developing countries

2002

KEYWORDS:

| Vuln- | Frag- | Frail- |
| --- | --- | --- |
| x |  |  |

*In a social context where women and children are vulnerable, they may be excluded from participating in research that is likely to benefit them or, conversely, exploited for research purposes precisely because they are vulnerable.*

*We suggest that, as a matter of moral principle, the more powerful have a duty to refrain from exploiting to their own advantage the vulnerability of the weaker. Since those with power may always be tempted to misuse it, perhaps even for what they perceive as benevolent reasons, it is important to insist on this principle. We have a number of points to make about the principle. First, it can be regarded as a further implication of the principle of respect for persons, for in exploiting others we fail to give proper weight to their interests. Secondly, like the requirement of sensitivity to cultural differences, the duty not to exploit the vulnerable merits special attention in the context of developing countries, not least because outsiders and local citizens may well differ on just what counts as exploiting or taking advantage of the weakness of others. Thirdly, it is important that the duty not to exploit the vulnerable be observed uniformly by all individuals and organisations involved in research, to avoid unfairness and the danger of undermining the principle in practice. If only some sponsors act in accordance with the principle of non-exploitation, then such scrupulous sponsors would be disadvantaged in relation to unscrupulous sponsors. Fourthly, although the duty not to exploit the vulnerabilities of others falls on all, the nature of the obligation may change depending on who is involved. Hence, the principle of not exploiting the vulnerable does not mean that we simply take the current context of research related to healthcare in the developing world as unchallengeable and unalterable. Just as it is unacceptable that local political and economic elites should seek to pursue their own goals at the expense of populations participating in research, it is unacceptable that researchers should select populations which are economically or politically weak, and therefore vulnerable to exploitation, in order to test therapies more cheaply in order to benefit other, wealthier communities.*

*Vulnerability: whether guaranteeing substantial benefits for taking part in research is more likely to constitute an undue inducement because prospective participants are especially vulnerable, for example because they have a terminal or chronic illness.*

85. Tri-Council Policy Statement: Ethical Conduct for Research Involving Humans, 2nd Edition

2022

KEYWORDS:

| Vuln- | Frag- | Frail- |
| --- | --- | --- |
| x |  |  |

*Vulnerability is often caused by limited decision-making capacity, or limited access to social goods, such as rights, opportunities and power. Individuals or groups whose circumstances may make them vulnerable in the context of research have historically included children, the elderly, students, women, prisoners, those with mental health issues and those with diminished capacity for self-determination. Ethnocultural minorities and those who are institutionalized are other examples of groups who have, at times, been treated unfairly and inequitably in research, or have been excluded from research opportunities. People or groups whose circumstances cause them to be vulnerable or marginalized may need to be afforded special attention in order to be treated justly in research.*

*Researchers and REBs should consider whether the prospective participants (as individuals, groups or populations) are in circumstances that may make them vulnerable in the context of research. The existence of such circumstances may require greater effort to minimize risks to participants and/or maximize potential benefits.*

*Some groups have been unfairly included in research because they are convenient populations for research (e.g., prisoners, students, people with limited financial resources or those in other circumstances of vulnerability).*

*In addition to the vulnerability that arises from their developmental stage, children may also lack the decision-making capacity to decide whether to participate in research.*

*Individuals or groups whose circumstances may make them vulnerable in the context of research should not be inappropriately included or automatically excluded from participation in research on the basis of their circumstances.*

*Individuals should not automatically be considered vulnerable simply because of assumptions made about the vulnerability of the group to which they belong. Their particular circumstances shall be considered in the context of the proposed research project.*

*Researchers and REBs should recognize and address changes in a participant’s circumstances that may create, heighten, or attenuate their vulnerability, and provide special protections or consideration. An equitable distribution of research benefits can help ensure that individuals, groups, and communities whose circumstances may make them vulnerable in the context of research are not inappropriately included in research based on these circumstances.*

*Studies may involve participants whose circumstances make them highly vulnerable in the context of research because of the social and/ or legal stigmatization that is associated with their activity or identity, and who may have little trust in the law, social agencies or institutional authorities.*

*Individuals or groups may experience vulnerability to different degrees and at different times, depending on their circumstances.*

86. U.S. 45 CFR 46

2018

KEYWORDS:

| Vuln- | Frag- | Frail- |
| --- | --- | --- |
| x |  |  |

*If an IRB regularly reviews research that involves a category of subjects that is vulnerable to coercion or undue influence, such as children, prisoners, individuals with impaired decision-making capacity, or economically or educationally disadvantaged persons, consideration shall be given to the inclusion of one or more individuals who are knowledgeable about and experienced in working with these categories of subjects.*

*When some or all of the subjects are likely to be vulnerable to coercion or undue influence additional safeguards have been included in the study to protect the rights and welfare of these subjects.*

87. Universal Declaration on Bioethics and Human Rights

2005

KEYWORDS:

| Vuln- | Frag- | Frail- |
| --- | --- | --- |
| x |  |  |

*In applying and advancing scientific knowledge, medical practice and associated technologies, human vulnerability should be taken into account. Individuals and groups of special vulnerability should be protected and the personal integrity of such individuals respected.*

88. Universal Declaration on Bioethics and Human Rights: perspectives from Kenya and South Africa

2008

KEYWORDS:

| Vuln- | Frag- | Frail- |
| --- | --- | --- |
| x |  |  |

*Vulnerability—Article 8 This article holds that ‘‘individuals and groups of special vulnerability should be protected and the personal integrity of such individuals respected’’. The Kenyan human subjects guidelines give special instructions concerning research with underdeveloped communities, prisoners, married women in rural areas and pregnant or lactating women, while the vaccines guidelines state that the vulnerable and poor must be protected from exploitation. The South African guidelines invite ethics committees to be ‘‘especially vigilant when considering research proposals involving vulnerable populations’’ and contain detailed provisions for research involving pregnant women, foetuses, prisoners and vulnerable communities.*

89. Updating protections for human subjects involved in research. Project on Informed Consent, Human Research Ethics Group

1998

KEYWORDS:

| Vuln- | Frag- | Frail- |
| --- | --- | --- |
| x | x |  |

*Students are in an inherently subordinate position to their instructors and are therefore vulnerable to coercion. Obviously these considerations apply to others in status relationship, such as laboratory workers.*

*Institutional review boards need to ensure the protection of those with significant disabilities being recruited into research who might be vulnerable to undue influence.*

*Current regulations provide that IRBs include persons knowledgeable in the areas in which an IRB reviews proposals involving vulnerable subject.*
