# Supplementary material for: Vulnerability in research ethics: A systematic review of policy guidelines and documents
Source: PLoS One. 2025 Jul 1;20(7):e0327086. doi: 10.1371/journal.pone.0327086 (PMC12212517; doi:10.1371/journal.pone.0327086)
Supplement: S4 Table — (DOCX) [file pone.0327086.s004.docx]

**S5 Table: list of included documents with relative link**

| **N°*** | **NAME** | **LINK** |
| --- | --- | --- |
| 12 | A Model Regulatory Program for Medical Devices: An International Guide | https://iris.paho.org/bitstream/handle/10665.2/51975/9275123454_eng.pdf?sequence=1&isAllowed=y |
| 13 | A Proposal for the Retrospective Identification and Categorization of Older People with Functional Impairments in Scientific Studies: Recommendations of the Medication and Quality of Life in Frail Older Persons (MedQoL) Research Group | https://doi.org/10.1016/j.jamda.2018.11.008 |
| 14 | AGS Report on Engagement Related to the NIH Inclusion Across the Lifespan Policy | [https://doi.org/10.1111/jgs.15784](https://doi.org/10.1111/jgs.15784;best) |
| 1 | The Belmont Report. Principles and guidelines for the protection of human subjects of research | https://www.hhs.gov/ohrp/regulations-and-policy/belmont-report/read-the-belmont-report/index.html |
| 15 | Best Practices for Protecting Privacy in Health Research | https://cihr-irsc.gc.ca/e/documents/et_pbp_nov05_sept2005_e.pdf |
| 16 | Clinical Investigation of Medicinal Products in the Paediatric Population (E11) | https://www.ema.europa.eu/en/ich-e11r1-guideline-clinical-investigation-medicinal-products-pediatric-population-scientific-guideline |
| 17 | Clinical Trials and Biomedical Research | https://mmc.gov.my/wp-content/uploads/2019/11/Clinical-TrialsBiomedical-Research.pdf |
| 18 | Conducting Science in Disasters: Recommendations from the NIEHS Working Group for Special IRB Considerations in the Review of Disaster Related Research | <https://doi.org/10.1289/EHP2378> |
| 19 | Declaration of Helsinki | https://www.wma.net/policies-post/wma-declaration-of-helsinki/ |
| 20 | Doing No Harm and Getting It Right: Guidelines for Ethical Research with Immigrant Communities | https://onlinelibrary.wiley.com/doi/10.1002/cad.20042 |
| 21 | Doing the Right Thing: Outlining the DWP's approach to ethical and legal issues in social research | https://webarchive.nationalarchives.gov.uk/ukgwa/+/http://research.dwp.gov.uk/asd/asd5/wp11.pdf |
| 22 | Ethical Aspects of Clinical Research in Developing Countries | https://op.europa.eu/en/publication-detail/-/publication/6339dcbf-c156-4e7f-9e43-9928acf82118 |
| 23 | Ethical considerations for Clinical Trials on Medical Products conducted with the Paediatric Population | https://health.ec.europa.eu/document/download/c1f2ff4c-63d0-4118-a6d6-a78197f04922_en |
| 24 | Ethical Considerations in Biomedical HIV Prevention Trials | https://www.unaids.org/sites/default/files/media_asset/jc1399_ethical_considerations_en_0.pdf |
| 25 | Ethical Guidelines | https://the-sra.org.uk/common/Uploaded%20files/ethical%20guidelines%202003.pdf |
| 26 | Ethical Guidelines for Conducting Research Studies Involving Human Subjects | https://bmrcbd.org/application_form/EthicalGideline/files/basic-html/page1.html |
| 27 | Ethical Guidelines for Research on Human Subject in Thailand | https://www.fercit.org/file/Guideline_English_version.pdf |
| 28 | Ethics Guidelines for Human Biomedical Research | https://www.bioethics-singapore.gov.sg/files/publications/reports/ethics-guidelines-for-human-biomedical-research-full-report.pdf |
| 29 | Ethics in clinical research: the Indian perspective | <https://doi.org/10.4103/0250-474x.91564> |
| 30 | Ethics in Health Research: Principles, Structures, and Processes | https://knowledgehub.health.gov.za/elibrary/ethics-health-research-principles-processes-and-structures |
| 31 | EU-Code for Ethics for Socio-Economic Research | https://www.employment-studies.co.uk/system/files/resources/files/412.pdf |
| 32 | Framework for Research Ethics | https://www.polis.cam.ac.uk/system/files/documents/esrc-framework-for-research-ethics.pdf |
| 33 | Framework of Guidelines for Research in the Social Sciences and Humanities in Malawi | https://www.ncst.mw/wp-content/uploads/2014/03/NATIONAL-FRAMEWORK-OF-GUIDELINES-IN-SSH.pdf |
| 34 | Good Clinical Practice Guidelines | https://nafdac.gov.ng/wp-content/uploads/Files/Resources/Guidelines/CTD_Guidelines/NAFDAC-Good-Clinical-Practices-Guidelines-2020.pdf |
| 35 | Good Clinical Practice Guidelines for Clinical Research in India | https://cdsco.gov.in/opencms/opencms/en/Home/ |
| 36 | Guidance synthesis. Medical research for and with older people in Europe: proposed ethical guidance for good clinical practice: ethical considerations | Doi:[10.1007/s12603-013-0340-0](http://dx.doi.org/10.1007/s12603-013-0340-0) |
| 37 | Guide for research ethics committee members | https://www.coe.int/en/web/human-rights-and-biomedicine/guide-for-research-ethics-committees-members |
| 38 | Guide to Internet Research Ethics | https://www.forskningsetikk.no/en/guidelines/social-sciences-and-humanities/a-guide-to-internet-research-ethics/ |
| 39 | Guideline for Application to Conduct Clinical Trials in Liberia | https://clinregs.niaid.nih.gov/sites/default/files/documents/liberia/G-LibClinTrial.pdf |
| 40 | Guideline for Good Clinical Practice (GCP) in Sierra Leone | https://clinregs.niaid.nih.gov/sites/default/files/documents/sierra_leone/PBSL-GCP-Guideline-V2.pdf |
| 41 | Guideline for Regulating the Conduct of Clinical Trials Using Medicines in Human Participants | https://www.moh.gov.bw/Publications/drug_regulation/CLINICAL%20TRIAL%20GUIDELINES%20botswana%20v4-060312.pdf |
| 42 | Guidelines for Conducting Clinical Trials of Medicines, Food Supplements, Vaccines, and Medical Devices in Sierra Leone | https://clinregs.niaid.nih.gov/sites/default/files/documents/sierra_leone/G-SLClinTrial-PBSL.pdf |
| 43 | Guidelines for Good Clinical Practice E6 (and Integrated Addendums E6(R2)-(R3)) | https://database.ich.org/sites/default/files/ICH_E6%28R3%29_Step4_FinalGuideline_2025_0106.pdf |
| 44 | Guidelines for Including People with Disabilities in Research | https://canfasd.ca/wp-content/uploads/publications/Guidelines-for-Including-People-with-Disabilities-in-Research.pdf |
| 45 | Guidelines for Phase I Clinical Trials | https://www.abpi.org.uk/media/tnnpj2lb/guidelines-for-phase-i-clinical-trials-2018-edition-20180626.pdf |
| 46 | Guidelines for Research Among Children and Young People | https://info.lse.ac.uk/staff/divisions/research-and-innovation/research/Assets/Documents/PDF/NCB-guidelinesCYP-2011.pdf |
| 47 | Guidelines for Research Ethics in the Social Sciences and the Humanities | https://www.forskningsetikk.no/en/guidelines/social-sciences-and-humanities/guidelines-for-research-ethics-in-the-social-sciences-and-the-humanities/ |
| 48 | Guidelines on Ethics for Health Research in Tanzania | https://clinregs.niaid.nih.gov/sites/default/files/documents/tanzania/G-EthicsHR.pdf |
| 49 | Guidelines on Ethics for Medical Research, Reproductive Biology and Genetic Research | https://repository.library.georgetown.edu/handle/10822/517510?show=full |
| 50 | Guidelines on Regulating the Conduct of Clinical Trials in Human Participants | https://www.zamra.co.zm/wp-content/uploads/2023/05/Guidelines-on-Application-for-Clincal-Trial-Authorisation.pdf |
| 51 | Handbook for Good Clinical Research Practice (GCP): Guidance for Implementation | https://iris.who.int/bitstream/handle/10665/43392/924159392X_eng.pdf |
| 52 | Implementing Regulations of the Law of Ethics of Research on Living Creatures | https://researchcompliance.kaust.edu.sa/IBEC/guidelines/Implementing%20Regulations%20of%20the%20Law%20of%20Ethics%20of%20Research%20on%20Living%20Creatures_%20Version%203_2022.pdf |
| 53 | Institutional Review Board (IRB) Policies and Procedures Handbook | https://clinregs.niaid.nih.gov/sites/default/files/documents/liberia/G-UL-PIRE-IRB_2020.pdf |
| 54 | International Code of Marketing & Social Research Practices | https://www.ipsos.com/sites/default/files/2017-11/ICCESOMAR_Code_English.pdf |
| 55 | International Ethical Guidelines for Research Involving Humans (CIOMS) | https://cioms.ch/wp-content/uploads/2017/01/WEB-CIOMS-EthicalGuidelines.pdf |
| 56 | Malaysian Phase I Clinical Trial Guidelines | https://www.ummc.edu.my/files/ethic/MCHRS/1%20General/Malaysian%20Phase%20I%20Clinical%20Trial%20Guidelines.pdf |
| 57 | Medical Products in Human Medicine Act | https://www.bda.bg/images/stories/documents/legal_acts/20210208_ZLPHM_English.pdf |
| 58 | Medical Research Involving Children | https://www.shu.ac.uk/~/media/home/research/files/ethics/medical-research-involving-children.pdf |
| 59 | National Ethical Guidelines for Biomedical and Health Research Involving Human Participants | https://ethics.ncdirindia.org/asset/pdf/ICMR_National_Ethical_Guidelines.pdf |
| 60 | National Ethical Guidelines for Biomedical Research Involving Children | https://thsti.res.in/pdf/National_Ethical_Guidelines_for_BioMedical_Research_Involving_Children.pdf |
| 61 | National Ethical Guidelines for Health and Health-Related Research | https://www.pchrd.dost.gov.ph/wp-content/uploads/2022/03/Annex-5.-National-Ethical-Guidelines-for-Health-and-Health-Related-Research-2017-1.pdf |
| 62 | National Guidelines for Ethical Conduct of Research Involving Human Subjects | https://www.researchgate.net/publication/280232379_Guidelines_for_Ethical_Conduct_of_Research_Involving_Human_Subjects_2008 |
| 63 | National Guidelines for Ethics Committees Reviewing Biomedical and Health Research During Covid-19 Pandemic | https://ijme.in/wp-content/uploads/2020/11/National-Guidelines_Meghna-A68-73.pdf |
| 64 | National Guidelines for Research Involving Humans as Research Participants | https://research.ciu.ac.ug/files/National_Guidelines_for_Research_Involving_Human_Participants.pdf |
| 65 | National Health Research Ethics Review Guideline, Fourth Edition | https://www.sewist.org.et/documents/29447/30917/7.+Ethiopian+National+Ethics+Guidelines.pdf/41c4d143-f80d-49eb-b879-6bef14a9da1c |
| 66 | National Statement on Ethical Conduct in Human Research | https://www.nhmrc.gov.au/about-us/publications/national-statement-ethical-conduct-human-research-2007-updated-2018 |
| 67 | Nigerian Code of Health Research Ethics | https://www.nhrec.net/nhrec/NCHRE_July%2007.pdf |
| 68 | Note for guidance on Good Clinical Practice (CPMP/ICH-135/95) | https://www.tga.gov.au/sites/default/files/ich13595an.pdf |
| 69 | Personal Information in Biomedical Research | https://www.bioethics-singapore.gov.sg/files/publications/reports/personal-informations-in-biomedical-research-full-report.pdf |
| 70 | Policy for the Protection and Welfare of Vulnerable Adults and the Management of Allegations of Abuse | https://avista.ie/wp-content/uploads/2022/08/DOCS-020-Policy-for-the-Protection-and-Welfare-of-Vulnerable-Adults-and-Management-of-Allegations-of-Abuse-2.pdf |
| 71 | Policy Statement Regarding Enrollment of Children in Research in Nigeria | https://nhrec.net/nhrec/Final%20NHREC%20Policy%20Statement%20on%20Enrollment%20of%20Children%20in%20Research.pdf |
| 72 | Qualitative methods in end-of-life research: Recommendations to enhance the protection of human subjects | https://www.sciencedirect.com/science/article/pii/S0885392403000605 |
| 73 | Recommendation (99) 4 on principles concerning the legal protection of incapable adults | https://www.coe.int/t/dg3/healthbioethic/texts_and_documents/rec(99)4e.pdf |
| 74 | Regulation (EU) 2017/745 of the European Parliament and of the Council of 5 April 2017 on medical devices | https://eur-lex.europa.eu/eli/reg/2017/745/oj/eng |
| 75 | Regulation No. 536/2014 of the European Parliament and of the Council on Clinical Trials on Medicinal Products for Human Use, Repealing Directive 2001/20/EC | https://eur-lex.europa.eu/eli/reg/2014/536/oj/eng |
| 76 | Regulations Relating to Research with Human Participants No. R719 | https://www.gov.za/documents/notices/national-health-act-regulations-research-human-participants-19-sep-2014 |
| 77 | Research Consent for Cognitively Impaired Adults. Recommendations for Institutional Review Boards and Investigators | https://www.researchgate.net/publication/232197811_Research_Consent_for_Cognitively_Impaired_Adults_Recommendations_for_Institutional_Review_Boards_and_Investigators |
| 78 | Research Ethics Framework (REF) | https://www.york.ac.uk/media/abouttheuniversity/governanceandmanagement/governance/ethicscommittee/hssec/documents/ESRC_Re_Ethics_Frame_.pdf |
| 79 | Research Ethics Policy and Procedures | https://www.uwi.edu/salises-mona/sites/salises-mona/files/PDF/policy_and_procedures_on_research_ethics2011.pdf |
| 80 | Research Governance Framework | https://www.gov.bm/sites/default/files/doh_research_governance_framework_2008_0.pdf |
| 81 | Resolution CNS No. 466/2012 on Guidelines and Rules for Research Involving humans Subjects | https://www.researchgate.net/publication/261609514_What_changes_in_Research_Ethics_in_Brazil_Resolution_no_46612_of_the_National_Health_Council |
| 82 | South African Good Clinical Practice: Clinical Trial Guidelines | https://www.sahpra.org.za/wp-content/uploads/2021/06/SA-GCP-2020_Final.pdf |
| 83 | Standards and Operational Guidance for Ethics Review of Health-Related Research with Human Participants | https://www.ncbi.nlm.nih.gov/books/NBK310666/ |
| 84 | The ethics of research related healthcare in developing countries | https://www.nuffieldbioethics.org/wp-content/uploads/Ethics-of-research-related-to-healthcare-in-developing-countries.pdf |
| 85 | Tri-Council Policy Statement: Ethical Conduct for Research Involving Humans | https://ethics.gc.ca/eng/policy-politique_tcps2-eptc2_2022.html |
| 86 | U.S. 45 CFR 46 | https://www.hhs.gov/ohrp/regulations-and-policy/regulations/45-cfr-46/index.html |
| 87 | Universal Declaration on Bioethics and Human Rights | https://www.unesco.org/en/ethics-science-technology/bioethics-and-human-rights |
| 88 | Universal Declaration on Bioethics and Human Rights: perspectives from Kenya and South Africa | https://pubmed.ncbi.nlm.nih.gov/18240025/ |
| 89 | Updating protections for human subjects involved in research. Project on Informed Consent, Human Research Ethics Group | https://pubmed.ncbi.nlm.nih.gov/9851484/ |
