# Supplementary material for: Vulnerability in research ethics: A systematic review of policy guidelines and documents
Source: PLoS One. 2025 Jul 1;20(7):e0327086. doi: 10.1371/journal.pone.0327086 (PMC12212517; doi:10.1371/journal.pone.0327086)
Supplement: S5 Table — (DOCX) [file pone.0327086.s005.docx]

**S6 Table: table of all data extracted from the primary research sources**

| **NAME** | **KEYWORDS** | | |
| --- | --- | --- | --- |
|  | **VULN-** | **FRAG-** | **FRAIL-** |
| A Model Regulatory Program for Medical Devices: An International Guide | x |  |  |
| A Proposal for the Retrospective Identification and Categorization of Older People With Functional Impairments in Scientific Studiesd: Recommendations of the Medication and Quality of Life in Frail Older Persons (MedQoL) Research Group | x |  | x |
| AGS Report on Engagement Related to the NIH Inclusion Acrossthe Lifespan Policy | x |  | x |
| Belmont Report. Principles and guidelines for the protection of human subjects of research | x |  |  |
| Best Practices for Protecting Privacy in Health Research | x |  |  |
| Clinical Investigation of Medicinal Products in the Paediatric Population (E11) | x |  |  |
| Clinical Trials and Biomedical Research | x |  |  |
| Conducting Science in Disasters: Recommendations from the NIEHS Working Group for Special IRB Considerations in the Review of Disaster Related Research | x |  | x |
| Declaration of Helsinki | x |  |  |
| Doing No Harm and Getting It Right: Guidelines for Ethical Research with Immigrant Communities | x | x |  |
| Doing the Right Thing: Outlining the DWP's approach to ethical and legal issues in social research | x |  | x |
| Ethical Aspects of Clinical Research in Developing Countries | x |  |  |
| Ethical considerations for Clinical Trials on Medical Products conducted with the Paediatric Population | x |  |  |
| Ethical Considerations in Biomedical HIV Prevention Trials | x |  |  |
| Ethical Guidelines | x |  |  |
| Ethical Guidelines for Conducting Research Studies Involving Human Subjects | x |  |  |
| Ethical Guidelines for Research on Human Subject in Thailand | x |  |  |
| Ethics Guidelines for Human Biomedical Research | x |  |  |
| Ethics in clinical research: the Indian perspective | x |  |  |
| Ethics in Health Research: Principles, Structures, and Processes | x |  |  |
| EU-Code for Ethics for Socio-Economic Research | x |  |  |
| Framework for Research Ethics | x |  |  |
| Framework of Guidelines for Research in the Social Sciences and Humanities in Malawi | x |  |  |
| Good Clinical Practice Guidelines | x |  |  |
| Good Clinical Practice Guidelines for Clinical Research in India | x |  |  |
| Guidance synthesis. Medical research for and with older people in Europe: proposed ethical guidance for good clinical practice: ethical considerations | x |  | x |
| Guide for research ethics committee members | x |  |  |
| Guide to Internet Research Ethics | x |  |  |
| Guideline for Application to Conduct Clinical Trials in Liberia | x |  |  |
| Guideline for Good Clinical Practice (GCP) in Sierra Leone | x |  |  |
| Guideline for Regulating the Conduct of Clinical Trials Using Medicines in Human Participants | x |  |  |
| Guidelines for Conducting Clinical Trials of Medicines, Food Supplements, Vaccines, and Medical Devices in Sierra Leone | x |  |  |
| Guidelines for Good Clinical Practice E6 (and Integrated Addendums E6(R2) | x |  |  |
| Guidelines for Including People with Disabilities in Research | x |  |  |
| Guidelines for Phase I Clinical Trials | x |  |  |
| Guidelines for Research Among Children and Young People | x |  |  |
| Guidelines for Research Ethics in the Social Sciences, Law, and the Humanities | x |  |  |
| Guidelines on Ethics for Health Research in Tanzania | x |  |  |
| Guidelines on Ethics for Medical Research, Reproductive Biology and Genetic Research | x |  |  |
| Guidelines on Regulating the Conduct of Clinical Trials in Human Participants | x |  |  |
| Handbook for Good Clinical Research Practice (GCP): Guidance for Implementation | x |  |  |
| Implementing Regulations of the Law of Ethics of Research on Living Creatures | x |  |  |
| Institutional Review Board (IRB) Policies and Procedures Handbook | x |  |  |
| International Code of Marketing & Social Research Practices | x |  |  |
| International Ethical Guidelines for Research Involving Humans | x | x | x |
| Malaysian Phase I Clinical Trial Guidelines | x |  |  |
| Medical Products in Human Medicine Act | x |  |  |
| Medical Research Involving Children | x |  |  |
| National Ethical Guidelines for Biomedical and Health Research Involving Human Participants | x | x |  |
| National Ethical Guidelines for Biomedical Research Involving Children | x | x |  |
| National Ethical Guidelines for Health and Health-Related Research | x |  | x |
| National Guidelines for Ethical Conduct of Research Involving Human Subjects | x |  |  |
| National Guidelines for Ethics Committees Reviewing Biomedical and Health Research During Covid-19 Pandemic | x |  |  |
| National Guidelines for Research Involving Humans as Research Participants | x |  |  |
| National Health Research Ethics Review Guideline, Fourth Edition | x |  |  |
| National Statement on Ethical Conduct in Human Research | x |  |  |
| Nigerian Code of Health Research Ethics | x |  |  |
| Note for guidance on Good Clinical Practice (CPMP/ICH-135/95) | x |  |  |
| Personal Information in Biomedical Research | x |  |  |
| Policy for the Protection and Welfare of Vulnerable Adults and the Management of Allegations of Abuse | x |  |  |
| Policy Statement Regarding Enrollment of Children in Research in Nigeria | x |  |  |
| Qualitative methods in end-of-life research: Recommendations to enhance the protection of human subjects | x |  |  |
| Recommendation (99) 4 on principles concerning the legal protection of incapable adults | x |  |  |
| Regulation (EU) 2017/745 of the European Parliament and of the Council of 5 April 2017 on medical devices | x |  |  |
| Regulation No. 536/2014 of the European Parliament and of the Council on Clinical Trials on Medicinal Products for Human Use, Repealing Directive 2001/20/EC | x |  | x |
| Regulations Relating to Research with Human Participants No. R719 | x |  |  |
| Research Consent for Cognitively Impaired Adults. Recommendations for Institutional Review Boards and Investigators | x |  |  |
| Research Ethics Framework (REF) | x |  |  |
| Research Ethics Policy and Procedures | x |  |  |
| Research Governance Framework | x |  |  |
| Resolution CNS No. 466/2012 on Guidelines and Rules for Research Involving humans Subjects | x |  |  |
| South African Good Clinical Practice: Clinical Trial Guidelines | x |  |  |
| Standards and Operational Guidance for Ethics Review of Health-Related Research with Human Participants | x |  |  |
| The ethics of research related healthcare in developing countries | x |  |  |
| Tri-Council Policy Statement: Ethical Conduct for Research Involving Humans | x |  |  |
| U.S. 45 CFR 46 | x |  |  |
| Universal Declaration on Bioethics and Human Rights | x |  |  |
| Universal Declaration on Bioethics and Human Rights: perspectives from Kenya and South Africa | x |  |  |
| Updating protections for human subjects involved in research. Project on Informed Consent, Human Research Ethics Group | x | x |  |

**Specifics**:

| **First extractor** | **External Check** | **Date of first Extraction** | **Date of Updates** |
| --- | --- | --- | --- |
| AG | GB + VS | November 2023 | 1. July 2024 2. May 2025 |
