# Supplementary material for: Vulnerability in research ethics: A systematic review of policy guidelines and documents
Source: PLoS One. 2025 Jul 1;20(7):e0327086. doi: 10.1371/journal.pone.0327086 (PMC12212517; doi:10.1371/journal.pone.0327086)
Supplement: S1 Checklist — (DOCX) [file pone.0327086.s007.docx]

**S7 Checklist: VULNERABILITY CHECKLIST**

**EXPLANATORY SECTION (for the experimenter own reading)**

Vulnerability in clinical research may be defined as *the increased risk of being wronged or of incurring additional harm (*Helsinki 2024).

In being enrolled as research participants, some individuals, groups, and/or communities may find themselves in *conditions of physical and/or psychological vulnerability*.

Conditions of vulnerability may be determined by factors which may be *fixed/inherent*, *contextual/dynamic*, or *both*.

- As an example of fixed/inherent condition of vulnerability, we may include “*mental disability*”. Research participants affected by mental disability may be incapable of protecting their own interests, may have a limited or absent capacity to consent/to refuse consent, etc.
- As an example of contextual/dynamic condition of vulnerability we may include “pregnancy”. There are some research settings where pregnancy represents a condition of vulnerability, for instance in pharmacological clinical trials, since the experimental compound may affect the fetal development and the health of the mother. Differently, there are other settings where pregnancy may or may not represent a condition of vulnerability: an observational study asking sensitive questions to pregnant woman (e.g. about their depression), may exacerbate pregnancy-related conditions of psychological vulnerability, while other observational studies asking routinary or less sensitive questions to pregnant woman may not have any impact in this regard.

Usually, conditions that define vulnerability may be grouped in the following three (potentially overlapping) categories:

- Consent-based conditions for vulnerability: vulnerability stems from a lack of capacity to provide free and informed consent with respect to participation in research, due to a variety of conditions, such as undue influence and reduced autonomy;
- Harm-based conditions for vulnerability: vulnerability follows from an unfair assessment between risks and benefits which may lead research participants to be exposed to a higher probability of incurring harm during research
- Justice-based conditions for vulnerability: vulnerability follows from not being able, by the side of participants, to benefit from a fair distribution of resources and services.

Typical categories of potentially vulnerable individuals/groups include*:

- Children
- People with learning disabilities or cognitive impairments
- People with mental disabilities
- The elderly
- Subordinates
- Pregnant women
- People with serious illnesses (e.g., people with a terminal illness, people with multiple chronic conditions, etc.)
- Institutionalized persons (e.g., people in nursing homes, prisoners)
- ethnic minority groups

** please note that this list is not exhaustive*

Clinical research with individuals, groups, or communities in situations of particular vulnerability is justified provided that the following conditions are all simoultaneously respected:

- The research is responsive to the health needs and priorities of the vulnerable individuals, groups, or communities;
- The vulnerable individual, group, or community stands to benefit from the resulting knowledge, practices, or interventions.
- When the research cannot be carried out in a less vulnerable group or community, or when excluding them would perpetuate or exacerbate their disparities.

PLEASE, NOT THAT, IN LINE WITH THE LAST VERSION OF THE HELSINKI DECLARATION (2024) AND OTHER RELEVANT DOCUMENTS (CIOMS 2016, etc.), **VULNERABILITY *PER SE* DOES NOT REPRESENT AN ETHICALLY JUSTIFIABLE REASON FOR EXCLUDING ENTIRE GROUPS OF INDIVIDUALS FROM RESEARCH**.

**SECTION THAT HAS TO BE FILLED IN BY THE EXPERIMENTER**

1. *Does your research include vulnerable participants and/or raise/create conditions of vulnerability?*

*If yes, please report the category of vulnerable research participant included in your study, explaining in details the fixed/inherent and/or contextual/dynamic vulnerability potentially affecting your study*.

_________________________________________________________________________________

_________________________________________________________________________________

_________________________________________________________________________________

_________________________________________________________________________________

_________________________________________________________________________________

_________________________________________________________________________________

_________________________________________________________________________________

1. *What are the practical measures you will put in place in order to address and/or contain fixed/inherent and/or contextual/dynamic vulnerabilities? (e.g., strengthen the informed consent process, foresee a legal guardian when subjects are unable to consent first-hand; constantly monitor the study and its effects, etc.)*

_________________________________________________________________________________

_________________________________________________________________________________

_________________________________________________________________________________

_________________________________________________________________________________

_________________________________________________________________________________

_________________________________________________________________________________

_________________________________________________________________________________

_________________________________________________________________________________

_________________________________________________________________________________
