# Supplementary material for: Vulnerability in research ethics: A systematic review of policy guidelines and documents
Source: PLoS One. 2025 Jul 1;20(7):e0327086. doi: 10.1371/journal.pone.0327086 (PMC12212517; doi:10.1371/journal.pone.0327086)
Supplement: Attachment — Submitted filename: Rebuttal Letter_July 2024.docx [file pone.0327086.s008.docx]

PONE-D-24-21976

Dear Dr. Sanchini,

Thank you for submitting your manuscript for review by PLOS ONE. After careful consideration, we have decided that your manuscript does not meet our standards for systematic and scoping reviews. We are therefore rejecting your manuscript.

PLOS ONE considers systematic reviews and meta-analyses for publication; however, these submissions must address a clear and defined research question; conduct a systematic and comprehensive literature review; and use clearly reported, reproducible, and systematic methods to identify, select, and critically appraise relevant research. Our author guidelines for systematic reviews are at [https://journals.plos.org/plosone/s/submission-guidelines#loc-systematic-reviews-and-meta-analyses](https://journals.plos.org/plosone/s/submission-guidelines#loc-systematic-reviews-and-meta-analyses).

In the case of your submission, we are concerned that the literature review was completed in  April 17, 2023. Systematic and scoping reviews should provide an up-to-date overview of the research topic covered. Since the literature search was completed over a year ago, we are concerned that this study does not meet this requirement.

As a result of these concerns, we cannot consider the manuscript for publication at PLOSONE at this time. However, if you are able to address the concerns discussed above, we may be able to consider a revised version of the manuscript. This would however require a substantial amount of work and thus, would be considered as a new submission. If you are able to revise the manuscript to respond to the concerns noted above, please refer to the original submission in the covering letter and accompany the submission by a rebuttal letter that responds to the comments made on the original manuscript.

I am sorry that we cannot be more positive on this occasion, but I hope you understand the reasons for this decision.

Yours sincerely,
 
Kalarani Anbarasan
Editorial Office
PLOS ONE

**

Milan, 11^th^ of July 2024

Dear Editor,

Following our submission of the systematic review titled “Vulnerability in research ethics. A systematic review of policy guidelines and documents” (PONE-D-24-21976), we thank you sincerely for having provided initial feedback on our manuscript and for having brought to our attention legitimate concerns regarding the timeliness of our work.

We certainly agree that systematic reviews normally require a very up-to-date overview of published literature. However, our work reviews a type of documents - policy guidelines - which are published much less frequently, compared to scientific papers.

Nevertheless, welcoming your observations, in order to verify that our work did not contain any obsolescence, we ran some checks on our data sources. Firstly, we looked up whether overview lists had been updated or new versions had been published; secondly, we re-performed database and grey literature searches, using the same keywords and search strings we used in April 2023.

The three overview lists of research ethics guidelines, which were our main data sources for the review, have not been updated. Therefore, our review still refers to the most up-to-date version of these listings.

The new search on databases and grey literature, which had allowed us to identify only a very small portion of the policy documents included in the review, this time around identified some new documents (respectively, 342 new documents were retrieved through database search, and 84 new documents were retrieved through grey literature search). However, after screening these documents, we could verify that none of them were relevant to our research questions.

In conclusion, no new policy document on the topic of vulnerability have been retrieved, and we may say that our systematic review does not present any obsolescence. All data and results are updated to July 7th 2024.

We have added a few lines in the Methodology section of our manuscript, reporting the verifications we have performed and we are hereby re-submitting our manuscript to your prestigious journal (lines 131-134).

We truly hope that we have now addressed the concerns you raised, and that, with these clarifications, you will consider our paper as ready to be submitted for the review process.

Kind regards,

Virginia Sanchini

*For the authors*
