# Supplementary material for: Vulnerability in research ethics: A systematic review of policy guidelines and documents
Source: PLoS One. 2025 Jul 1;20(7):e0327086. doi: 10.1371/journal.pone.0327086 (PMC12212517; doi:10.1371/journal.pone.0327086)
Supplement: Attachment — Submitted filename: Response to Reviewers.docx [file pone.0327086.s009.docx]

PONE-D-24-28751

Vulnerability in research ethics. A systematic review of policy guidelines and documents.

PLOS ONE

Dear Dr. Sanchini,

Thank you for submitting your manuscript to PLOS ONE. After careful consideration, we feel that it has merit but does not fully meet PLOS ONE’s publication criteria as it currently stands. Therefore, we invite you to submit a revised version of the manuscript that addresses the points raised during the review process.

Reviewers plenty of constructive comments and suggestions for improvement. You should take this as an opportunity to strengthen your article. However, you are not required to follow each and every recommendation and may disagree with reviewers on some aspects.

Comments from PLOS Editorial Office: We note that one or more reviewers has recommended that you cite specific previously published works. As always, we recommend that you please review and evaluate the requested works to determine whether they are relevant and should be cited. It is not a requirement to cite these works. We appreciate your attention to this request.

Please submit your revised manuscript by Mar 01 2025 11:59PM. If you will need more time than this to complete your revisions, please reply to this message or contact the journal office at [plosone@plos.org](mailto:plosone@plos.org). When you're ready to submit your revision, log on to <https://www.editorialmanager.com/pone/> and select the 'Submissions Needing Revision' folder to locate your manuscript file.

Please include the following items when submitting your revised manuscript:

- A rebuttal letter that responds to each point raised by the academic editor and reviewer(s). You should upload this letter as a separate file labeled 'Response to Reviewers'.
- A marked-up copy of your manuscript that highlights changes made to the original version. You should upload this as a separate file labeled 'Revised Manuscript with Track Changes'.
- An unmarked version of your revised paper without tracked changes. You should upload this as a separate file labeled 'Manuscript'.

If you would like to make changes to your financial disclosure, please include your updated statement in your cover letter. Guidelines for resubmitting your figure files are available below the reviewer comments at the end of this letter.

If applicable, we recommend that you deposit your laboratory protocols in protocols.io to enhance the reproducibility of your results. Protocols.io assigns your protocol its own identifier (DOI) so that it can be cited independently in the future. For instructions see: [https://journals.plos.org/plosone/s/submission-guidelines#loc-laboratory-protocols](https://eur02.safelinks.protection.outlook.com/?url=https%3A%2F%2Ftrack.editorialmanager.com%2FCL0%2Fhttps%3A%252F%252Fjournals.plos.org%252Fplosone%252Fs%252Fsubmission-guidelines%2523loc-laboratory-protocols%2F1%2F010f01947013f489-40176671-21ea-4f63-8c1c-fa586ee8bb80-000000%2FSO2wK63J_4tgF2uL1iEqt1vUN3EP9NCM2cjNAPrZhI4%3D194&data=05%7C02%7Cvirginia.sanchini%40unimi.it%7Cd2442a0340c1427c297708dd365007f3%7C13b55eef70184674a3d7cc0db06d545c%7C0%7C0%7C638726439962901693%7CUnknown%7CTWFpbGZsb3d8eyJFbXB0eU1hcGkiOnRydWUsIlYiOiIwLjAuMDAwMCIsIlAiOiJXaW4zMiIsIkFOIjoiTWFpbCIsIldUIjoyfQ%3D%3D%7C0%7C%7C%7C&sdata=ZoCA8lVrXkfko9hxDbKBA8PxddwInZNQyLd8emjEz44%3D&reserved=0). Additionally, PLOS ONE offers an option for publishing peer-reviewed Lab Protocol articles, which describe protocols hosted on protocols.io. Read more information on sharing protocols at [https://plos.org/protocols?utm_medium=editorial-email&utm_source=authorletters&utm_campaign=protocols](https://eur02.safelinks.protection.outlook.com/?url=https%3A%2F%2Ftrack.editorialmanager.com%2FCL0%2Fhttps%3A%252F%252Fplos.org%252Fprotocols%253Futm_medium%3Deditorial-email%2526utm_source%3Dauthorletters%2526utm_campaign%3Dprotocols%2F1%2F010f01947013f489-40176671-21ea-4f63-8c1c-fa586ee8bb80-000000%2FxGkOp0Rjx0vLPJPrIHJX8o2PQHQE9EX9ArRudXhKR1A%3D194&data=05%7C02%7Cvirginia.sanchini%40unimi.it%7Cd2442a0340c1427c297708dd365007f3%7C13b55eef70184674a3d7cc0db06d545c%7C0%7C0%7C638726439962928199%7CUnknown%7CTWFpbGZsb3d8eyJFbXB0eU1hcGkiOnRydWUsIlYiOiIwLjAuMDAwMCIsIlAiOiJXaW4zMiIsIkFOIjoiTWFpbCIsIldUIjoyfQ%3D%3D%7C0%7C%7C%7C&sdata=ZIkXYKnL2vSAmmpaAXjjApzSoYqYksUROAB4ttst2%2Fg%3D&reserved=0).

We look forward to receiving your revised manuscript.

Kind regards,

Alberto Molina Pérez, Ph.D.
Academic Editor
PLOS ONE

***R.*** *Dear Editor,*

*Thank you very much for having positively considered our manuscript and deem it to be a contribution that may advance the current state of bioethical knowledge regarding the concept of vulnerability in research ethics policy and guidelines.*

*We would like to thank the two Reviewers for their insightful and constructive comments that have helped us to further improve our manuscript.*

*We provide below a point-by-point response letter to the issues that were raised and that we have addressed in full in our revision.*

*As to the journal requirements, please find our reply below.*

*Very truly yours,*

*Virginia Sanchini*

*For the authors*

**

Journal Requirements:

When submitting your revision, we need you to address these additional requirements.

1. Please ensure that your manuscript meets PLOS ONE's style requirements, including those for file naming. The PLOS ONE style templates can be found at 
<https://journals.plos.org/plosone/s/file?id=wjVg/PLOSOne_formatting_sample_main_body.pdf> and 
<https://journals.plos.org/plosone/s/file?id=ba62/PLOSOne_formatting_sample_title_authors_affiliations.pdf>

***R.*** *We apologize for the oversights. We have now made style adjustments according to PLOS ONE’s requirements.*

2. Thank you for stating the following financial disclosure: This research is part the project “Emerging Technologies and vulnerabilities in aged care” (ElderTech), funded by Fondazione Cariplo, call “Social Research:
Science, Technology and Society”. Grant number: 2020-1322 
Please state what role the funders took in the study.  If the funders had no role, please state: ""The funders had no role in study design, data collection and analysis, decision to publish, or preparation of the manuscript."" 
If this statement is not correct you must amend it as needed. 
Please include this amended Role of Funder statement in your cover letter; we will change the online submission form on your behalf.

***R.*** *We have added this amended Role of Funder statement in the cover letter, as required.*

3. In the online submission form, you indicated that all data generated or analysed during this study are included in this published article.
Additionally, the full list of QUAGOL schema are not publicly available due to privacy
concern but are available from the corresponding author on reasonable request. 
All PLOS journals now require all data underlying the findings described in their manuscript to be freely available to other researchers, either 1. In a public repository, 2. Within the manuscript itself, or 3. Uploaded as supplementary information.
This policy applies to all data except where public deposition would breach compliance with the protocol approved by your research ethics board. If your data cannot be made publicly available for ethical or legal reasons (e.g., public availability would compromise patient privacy), please explain your reasons on resubmission and your exemption request will be escalated for approval.

***R.*** *We thank the Editor for this comment, which allows us to better clarify the nature of this work and the consequential choices we made about the availability of all generated data.*

*This work is a systematic review of policy documents, not clinical studies or scientific articles. Therefore, the primary data we analysed are all published regulatory documents, available online for public consultation. We have included all the links to the policy documents we analysed in Supporting Information section, S4 Table.*

*The full text of these policy documents is what underlies our findings. However, we also used QUAGOL schema to further extract data from our selected policy documents. The data extracted from our findings are reported in S5 Table and in S3 Appendix.*

4. As required by our policy on Data Availability, please ensure your manuscript or supplementary information includes the following:

A numbered table of all studies identified in the literature search, including those that were excluded from the analyses.

For every excluded study, the table should list the reason(s) for exclusion.

If any of the included studies are unpublished, include a link (URL) to the primary source or detailed information about how the content can be accessed.

***R.*** *We have added a table with the full list of the documents that underwent the screening process (1028 records) in S1 Table and S2 Table.*

*The reasons for exclusion are reported in the tables.*

*All the documents we analysed in our systematic review are published guidelines.*

A table of all data extracted from the primary research sources for the systematic review and/or meta-analysis. The table must include the following information for each study:

Name of data extractors and date of data extraction

Confirmation that the study was eligible to be included in the review.

All data extracted from each study for the reported systematic review and/or meta-analysis that would be needed to replicate your analyses.

If data or supporting information were obtained from another source (e.g. correspondence with the author of the original research article), please provide the source of data and dates on which the data/information were obtained by your research group.

***R.*** *As stated above, the data analysed in this systematic review consist of the policy documents themselves. Since this is not primary research, there were no studies to analyse and no data such as “sample size”, “location”, “research method”, etc. to be extracted from the documents under review. Information relevant to our research questions was obtained from the full texts of policy documents.*

*Considering that the full text of policy documents was the only actual extracted data, we have added the name of data extractors and the date of extraction in S5 Table.*

If applicable for your analysis, a table showing the completed risk of bias and quality/certainty assessments for each study or outcome.  Please ensure this is provided for each domain or parameter assessed. For example, if you used the Cochrane risk-of-bias tool for randomized trials, provide answers to each of the signalling questions for each study. If you used GRADE to assess certainty of evidence, provide judgements about each of the quality of evidence factor. This should be provided for each outcome.

An explanation of how missing data were handled.

***R.*** *These assessments do not apply to the type of documents we analysed.*

This information can be included in the main text, supplementary information, or relevant data repository. Please note that providing these underlying data is a requirement for publication in this journal, and if these data are not provided your manuscript might be rejected.  

[Note: HTML markup is below. Please do not edit.]

**Reviewers' comments:**

Reviewer's Responses to Questions

**Comments to the Author**

1. Is the manuscript technically sound, and do the data support the conclusions?

The manuscript must describe a technically sound piece of scientific research with data that supports the conclusions. Experiments must have been conducted rigorously, with appropriate controls, replication, and sample sizes. The conclusions must be drawn appropriately based on the data presented.

Reviewer #1: Yes

Reviewer #2: Yes

2. Has the statistical analysis been performed appropriately and rigorously?

Reviewer #1: Yes

Reviewer #2: N/A

3. Have the authors made all data underlying the findings in their manuscript fully available?

The [PLOS Data policy](https://eur02.safelinks.protection.outlook.com/?url=http%3A%2F%2Ftrack.editorialmanager.com%2FCL0%2Fhttp%3A%252F%252Fwww.plosone.org%252Fstatic%252Fpolicies.action%2523sharing%2F1%2F010f01947013f489-40176671-21ea-4f63-8c1c-fa586ee8bb80-000000%2FMmCzDwpTGVMIyWO-yQmDuKHtcU4TR5ZE9-EtRvSjXtc%3D194&data=05%7C02%7Cvirginia.sanchini%40unimi.it%7Cd2442a0340c1427c297708dd365007f3%7C13b55eef70184674a3d7cc0db06d545c%7C0%7C0%7C638726439962947234%7CUnknown%7CTWFpbGZsb3d8eyJFbXB0eU1hcGkiOnRydWUsIlYiOiIwLjAuMDAwMCIsIlAiOiJXaW4zMiIsIkFOIjoiTWFpbCIsIldUIjoyfQ%3D%3D%7C0%7C%7C%7C&sdata=IwGR%2FOA2%2B4g0RSoMsZZCP%2Fd3Ric03DBu1UjSx8ocHic%3D&reserved=0) requires authors to make all data underlying the findings described in their manuscript fully available without restriction, with rare exception (please refer to the Data Availability Statement in the manuscript PDF file). The data should be provided as part of the manuscript or its supporting information, or deposited to a public repository. For example, in addition to summary statistics, the data points behind means, medians and variance measures should be available. If there are restrictions on publicly sharing data—e.g. participant privacy or use of data from a third party—those must be specified.

Reviewer #1: Yes

Reviewer #2: Yes

4. Is the manuscript presented in an intelligible fashion and written in standard English?

PLOS ONE does not copyedit accepted manuscripts, so the language in submitted articles must be clear, correct, and unambiguous. Any typographical or grammatical errors should be corrected at revision, so please note any specific errors here.

Reviewer #1: Yes

Reviewer #2: Yes

5. Review Comments to the Author

Please use the space provided to explain your answers to the questions above. You may also include additional comments for the author, including concerns about dual publication, research ethics, or publication ethics. (Please upload your review as an attachment if it exceeds 20,000 characters)

**Reviewer #1**: The systematics appears to be well conducted and the search strategy with which the search was carried out appears to be complete with relevant terminology. In my opinion, the authors should better explain the concept of disability in light of recent literature, and how it relates to that of vulnerability (for example, see doi: 10.1016/j.arrct.2020.100079; doi: 10.1007/s11196-022-09946-x).

In health care, failure to recognize a disability makes the individual even more vulnerable, increasing his or her social disadvantage. Please elaborate on this ethical aspect in the Discussion section.

***R.*** *We thank the reviewer for this comment. We have added a few sentences and references to better specify this issue in the Discussion Section from line 603 to line 617.*

Finally, the problem of the use of new digital technologies in people who are vulnerable and require state-of-the-art health care could be a detriment to the person's own dignity and human relationships with health care providers. This concept should also be mentioned by the authors among the various ethical issues raised (for example, see doi: 10.1080/17483107.2023.2288241,doi: 10.3389/fmed.2024.1437280, doi: 10.1097/MD.0000000000036671).

***R.*** *We thank the reviewer for this comment. We have added a few sentences and references to better specify this issue in the Conclusion Section from line 781 to line 797.*

**Reviewer #2:** This manuscript presents a systematic review investigating how vulnerability is conceptualized and operationalized in research ethics policy documents. The authors analyzed 79 policy documents from guidelines/databases/grey lit, identifying recurring themes such as a tendency to define vulnerable groups rather than vulnerability itself, and a link between vulnerability and informed consent. The manuscript reveals inconsistencies in defining vulnerability (especially mostly failing to define) and identifying different vulnerable populations across different documents (resulting in unwieldy heterogeneity). There is much to like in the manuscript but, there is also room for improvement. I hope the following comments will be useful to the author(s) as they make revisions.

1. Motivation: It would be useful to motivate better why scholars and practitioners need to care about vulnerability as a regulatory category for research ethics.

a. This is especially important given that it has been critiqued as having some fundamental limitations (e.g., Levine et al 2004). In many ways, the authors’ systematic review serves to deepen concerns about the challenges with little guidance on how to improve (see my note below). They end the manuscript with a note about providing a starting point for development, but need to do more and then motivate the manuscript better. If the limitations are addressable, then the authors should preview this up front and develop in the manuscript.

b. An alternative perspective on this is that although current practice is not uniform or necessarily principled, it largely gets the job done. From this perspective, in practice vulnerability considerations act to add some redundancy to the broader principles (e.g., Belmont). So even if researchers do not address vulnerability carefully, it is not meant to be a fail-safe. In the manuscript the authors of course raise some rationale (e.g., include with protections versus exclude; etc), but could motivate the paper better.

***R.*** *We thank the reviewer for these comments, which gave us an opportunity to better motivate our work.*

*In our opinion the lack of clear, specific guidance on vulnerability results in ethics committees and researchers dealing with vulnerable research subjects in a sub-optimal way. Indeed, the ambiguity around vulnerability in research ethics policy documents has proven to be problematic in concrete scenarios.*

*Firstly, research ethics committees, in their documentation, usually require a statement about the enrolment of vulnerable populations, but they often have no expert knowledge of what this means exactly, so they rely on lists of vulnerable groups, which are the most practical guidance available. This, however, may imply that different research ethics committees use different lists, resulting in a differential treatment of research subjects, with potentially inequitable outcomes.*

*Secondly, researchers often have too little guidance on how to deal with vulnerable subjects, therefore they may end up excluding some populational groups defined as vulnerable, due to difficulties in managing them appropriately.*

*Finally, but most importantly, research subjects risk to suffer disparities or underrepresentation in research, the latter a practice that also more recent documents has strongly criticized (e.g. 2024 version of the Helsinki Declaration).*

*However, it is true, as the reviewer suggests, that our work clearly and unequivocally highlights the heterogeneity that exists among policy documents, and that this may deepen rather than taming existing concerns on the topic.*

*Despite what above, in our view this work could first serve experimenters to become more aware of the heterogeneity that exists in guidelines, so that, while regulatory actions are being carried out, they can start taking responsibility for informed, individual choices when dealing with vulnerable research subjects, in order to gradually move from a labelling approach to an analytical approach in the practice of their research.*

*On a higher level, this work may also serve members of research ethics committees to better navigate among different guidelines and thus refine their requests to experimenters in dealing with vulnerable research participants.*

*Nonetheless, we recognize that, in order to achieve this goal, more practical guidance is needed. Therefore, as the reviewer rightfully suggested, we committed to address some of the limitations of research ethics policy documents, by developing a preliminary tool for reflection on the management of vulnerable populations in research settings. We have drafted a checklist that could be used by experimenters and ethics committee members when dealing with and making decisions about vulnerable subjects (see S6 Checklist).*

2. Coding & Analysis

a. I really like the data coding and think that researchers need to engage seriously with more comprehensive understandings. I suspect that there is a lot that researchers could learn from the data beyond what the authors report. On that, I am actually unclear from the submission meta-information what data will be available. The authors mention privacy concerns, but it is unclear what privacy issues are in question given that these are mostly public documents? And data summarizing gated materials can be posted, even though the gated materials themselves cannot be. I encourage making as much data public as possible and more clearly discussing which data cannot be (and why).

***R.*** *The Reviewer is right in saying that all data underlying our work are already public. Indeed, we have included in our manuscript references and links of all the policy documents we analyzed, so they can be easily retrieved by researchers who want to learn more from the data (see Supporting Information section, S4 Table).*

*The full text of these policy documents is what underlies our findings. However, we also used QUAGOL schema to further extract data from our selected policy documents. The data extracted from our findings are reported in S5 Table and in S3 Appendix.*

b. It would be useful to understand better the implications of excluding non-English-language documents. The authors should consider randomly sampling a subset of papers, translating them, then coding and comparing against the English language documents.

***R.*** *We thank the Reviewer for this observation; however, we believe that excluding non-English language documents did not have relevant implications on our research. Originally, the focus of this work was on international documents. On second thoughts, we decided to include also documents which were translated in English to broaden the perspective and include documents which, thanks to the translation, might have had resonance outside national borders.*

*However, following the reviewer observation, we reported the fact of not having included non-English language documents as one of the limitations of this study.*

c. The top of page 15 has an error. It says that “seventy-two documents give a definition…” but this should be seventy-two docs that identify groups/populations as vulnerable (Q2). On the bottom of page 14, it says that only ten documents gave definitions (Q1).

***R.*** *We thank the reviewer for pointing out this mistake, which has been corrected in the revised manuscript.*

3. Value added and takeaways

a. There is clearly value in conducting the systematic review, especially because it is important to understand any boundary conditions of our current understanding. The current manuscript is thus valuable in helping us probe those boundaries at least to the extent that English language sources represent the broader population of documents.

b. In my view, however, the takeaways need more attention. The findings seem to echo what earlier studies have already found and discussed and the authors of this manuscript do not draw out novel implications, or if they did the novel implications currently get lost and need more attention. For examples, others have discussed:

i. categories rather than conceptualization or without justification (e.g., Luna 2009 and Findley et al 2024 who the authors do not cite);
ii. the reductionist view of vulnerability (e.g., Meek Lange et al. 2013 [Ref: #90]);
iii. the foundation versus reflective view of vulnerability (Bracken-Roche et al. 2017 [Ref: #91]);
iv. the disproportionate emphasis on consent (e.g. Hurst 2008 whom the authors do not cite)

***R.*** *The reviewer is right. We added relevant references (Luna 2009, Hurst 2008 and Findley et al 2024) within the Discussion section.*

c. Possible avenues for development:

i. I was quite intrigued by the authors note that the emphasis on categories is different from clinical ethics (page 29, including Reference #89). The authors seem to think that the difference is explained simply, which may be the case. But are there unexplored connections or opportunities for research ethics to learn from clinical ethics? It would be useful to unpack this possibility to a greater degree.

***R.*** *We thank the reviewer for these comments, and, above all, for his/her suggestions for improvements.*

*As to point* ***i****., the authors tacitly referred to the fact that clinical ethics (and philosophical) debates surrounding vulnerability mostly distinguish between vulnerability as a condition generally affecting human beings as such (namely, what has been labelled elsewhere as “basic human”, “intrinsic”, “ontological”, “existential”, etc., vulnerability), and vulnerability as a condition of minority and/or disadvantage affecting only some agents, due to specific situational conditions (namely, what has been labelled elsewhere as “situational”, “contingent”, “extrinsic”, etc., vulnerability). After careful consideration, we believe the reviewer is right in saying that (still unexplored) connections between conceptualisations of vulnerability in research ethics and conceptualisations of the same notion in clinical ethics may be useful, and that research ethics may be enriched by using debates and ways to frame the same notion belonging to clinical ethics. Therefore, we better clarified this connection in the Discussion section (“Vulnerability in research ethics and other domains of applied ethics”), and we also introduced the distinction between basic human (which we referred to as “fixed/inherent”) and situational (“contextual/dynamic”) vulnerability in the vulnerability checklist (see below, reply to point ii).*

ii. The authors conclude the manuscript with a note about the need to “draft new, comprehensive, and most importantly, unambiguous policy documents” (p. 34). While it is true that some do not see vulnerability as a big concern, many do see it as such but are unclear on how to attain the ideal approach. It would be helpful if the authors could develop this point further. As they say, this is a starting point, but it remains a highly murky starting point without more concrete guidance.

1. Did any of the policy/guidance documents stand out as “model” documents that could serve as a basis for advancing the conversation?
2. Did some combination of documents provide just the right mix of factors that could serve as a basis?
3. Is there some combination of the observations the authors make in their results discussion that, if put together, provide concrete steps?
4. Are there ways in which key components of clinical ethics could map to research ethics in ways that provide more guidance?
5. Are there ways the authors could improve on what Findley et al (PNAS; 2024) proposed? After coding a large set of legislative/regulatory documents for 100+ countries, they also proposed a “starting point” framework with concrete (though preliminary) ideas and discussion of stakeholders/incentives/norms.

I fear that without developing their final point, the authors of the current manuscript will simply be read as reinforcing conventional wisdom based on a larger corpus of documents.

***R.*** *We really thank the reviewer for this last comment, which prompted us to improve our work not just theoretically, but also from a practical standpoint. As reported in the Result section, what this systematic review has revealed is that there is a combination of documents which inspired most policy documents and guidelines, namely: the Belmont Report, the Helsinki Declaration, CIOMS Guidelines, and GCP Guidelines. This is an important finding per se, since it may suggest that a first step for seriously considering vulnerability in research ethics would be that experimenters are somehow exposed to this set of documents and prove to know them. However, to provide more concrete guidance, we also developed a checklist (what we defined as “Vulnerability Checklist”) which, in our view, should be filled in by the experimenters and submitted to the appointed research ethics committee, along with all the documentation required for submission. In this sense, we actually recognize that the TAPIR framework proposed by Findley and colleagues represents an extraordinary contribution for scholarly debates which are interested in a broader (not only research ethics but also social and political) and non-procedural definition of vulnerability. However, insofar as it is narrower and perhaps less demanding in its operationalization, we hope that our proposed checklist will be somehow able to complement Findley et al. more comprehensive effort. (Findley et al contribution, which was not present in the original reference list as published after the first submission of this review, has now been inserted in the current version of the manuscript).*
