# Supplementary material for: Vulnerability in research ethics: A systematic review of policy guidelines and documents
Source: PLoS One. 2025 Jul 1;20(7):e0327086. doi: 10.1371/journal.pone.0327086 (PMC12212517; doi:10.1371/journal.pone.0327086)
Supplement: Attachment — Submitted filename: Comments_FM.docx [file pone.0327086.s010.docx]

1. The authors have commendably addressed many of the previous concerns. However, I remain unconvinced that the revisions fully satisfy the points raised by Reviewer 2 regarding the conceptualisation and operationalisation of vulnerability. Specifically, while the authors motivated the significance of 'vulnerability' as a regulatory category in research ethics in response to the reviewer, simply noting this in the rebuttal may not be sufficient. Perhaps this crucial justification could be integrated directly into the manuscript's introduction or any other appropriate section. Without a robust and articulated rationale within the text itself, the paper's foundational argument regarding vulnerability remains potentially unpersuasive for both scholars and practitioners.
2. The proposed checklist warrants closer scrutiny. Does the data from the review inform its development, or is there evidence to support the utility of checklists in similar contexts? The authors may need to consider providing a compelling argument for how this checklist offers additional safeguards for vulnerable populations beyond the ethical considerations typically detailed in standard research protocols. As it stands, introducing a checklist risks appearing as an added burden on researchers without clearly demonstrating enhanced protection.
3. Finally, the assertion that research ethics committees often lack specific expertise regarding vulnerability, leading them to rely on lists of vulnerable groups, requires empirical support. It might be helpful to substantiate this claim with evidence from the existing literature on the functioning and expertise within research ethics review boards. Without such evidence, this statement risks undermining the credibility of current ethical review processes.
4. Line 32, 596, 599, - Use of the term/ phrase emerging themes

The current framing of theme emergence may inadvertently diminish the acknowledged role of the researcher in the analytical process. Some readers might contend that presenting themes as simply 'emerging' risks obscuring the active and interpretive work undertaken by the researcher in identifying, developing, and shaping these analytical categories. The phrasing could be perceived as downplaying the researcher's influence and agency in constructing meaning from the data.

To address this potential concern, the authors may consider explicitly articulating their role in the thematic analysis. Furthermore, incorporating a reflexivity statement would significantly strengthen the methodological rigour of the manuscript. By transparently reflecting on their potential biases, assumptions, and perspectives, the authors can provide readers with a clearer understanding of how their positionality may have shaped the data collection and interpretation process. This will enhance the trustworthiness and credibility of the findings.

5. Additional commemts

Line 632 – include page number on the reference after “to provide an analysis of vulnerability that does not render it vacuous, rescues its force and importance”

6. Line 769 -770 - The first sentence should be referenced.
7. 785-6 - Previous studies in older adults’ populations, consider “Previous studies in adult populations.”
