# Supplementary material for: Vulnerability in research ethics: A systematic review of policy guidelines and documents
Source: PLoS One. 2025 Jul 1;20(7):e0327086. doi: 10.1371/journal.pone.0327086 (PMC12212517; doi:10.1371/journal.pone.0327086)
Supplement: Attachment — Submitted filename: Response_to_reviewers_auresp_2.docx [file pone.0327086.s011.docx]

**Reviewer #3**

1. The authors have commendably addressed many of the previous concerns. However, I remain unconvinced that the revisions fully satisfy the points raised by Reviewer 2 regarding the conceptualisation and operationalisation of vulnerability. Specifically, while the authors motivated the significance of 'vulnerability' as a regulatory category in research ethics in response to the reviewer, simply noting this in the rebuttal may not be sufficient. Perhaps this crucial justification could be integrated directly into the manuscript's introduction or any other appropriate section. Without a robust and articulated rationale within the text itself, the paper's foundational argument regarding vulnerability remains potentially unpersuasive for both scholars and practitioners.

***R.*** *We thank the reviewer for this comment. The reviewer was right in pointing out that the paper would have benefit from better motivating the reasons why scholars and practitioners need to care about vulnerability as a regulatory category for research ethics. We have added a few sentences and references in the* ***Introduction*** *section, as requested (see lines 97-109).*

2. The proposed checklist warrants closer scrutiny. Does the data from the review inform its development, or is there evidence to support the utility of checklists in similar contexts? The authors may need to consider providing a compelling argument for how this checklist offers additional safeguards for vulnerable populations beyond the ethical considerations typically detailed in standard research protocols. As it stands, introducing a checklist risks appearing as an added burden on researchers without clearly demonstrating enhanced protection.

***R.*** *We thank the reviewer for this comment. In the* ***Discussion*** *section we explained in details to what extent the proposed checklist was informed and shaped by our research findings, therefore making explicit all the connections between study results and the checklist content (see lines 829-866).*

*However, although this tool was created by the authors of the review—who, through their diverse roles as researchers, study coordinators, and members of ethics committees, can in some way be considered representative of the perspectives that such a checklist should embody— the proposed checklist can only be regarded as a preliminary tool. Its actual usefulness, appropriateness, and effectiveness would require a study in which the tool is tested, something we propose to undertake in the near future should the reviewers consider the tool as potentially useful.*

3. Finally, the assertion that research ethics committees often lack specific expertise regarding vulnerability, leading them to rely on lists of vulnerable groups, requires empirical support. It might be helpful to substantiate this claim with evidence from the existing literature on the functioning and expertise within research ethics review boards. Without such evidence, this statement risks undermining the credibility of current ethical review processes.

***R.*** *The reviewer is right. This statement was based on our daily experience as researchers but also as ethics committees’ members. However, since we were not able to find published evidence on this issue, we did not include in the revised manuscript this sentence, as it cannot be generalizable, and, as rightly pointed out by the reviewer, may undermine the credibility of research ethics committee’ members.*

4. Line 32, 596, 599 - Use of the term/ phrase emerging themes

The current framing of theme emergence may inadvertently diminish the acknowledged role of the researcher in the analytical process. Some readers might contend that presenting themes as simply 'emerging' risks obscuring the active and interpretive work undertaken by the researcher in identifying, developing, and shaping these analytical categories. The phrasing could be perceived as downplaying the researcher's influence and agency in constructing meaning from the data.

To address this potential concern, the authors may consider explicitly articulating their role in the thematic analysis. Furthermore, incorporating a reflexivity statement would significantly strengthen the methodological rigour of the manuscript. By transparently reflecting on their potential biases, assumptions, and perspectives, the authors can provide readers with a clearer understanding of how their positionality may have shaped the data collection and interpretation process. This will enhance the trustworthiness and credibility of the findings.

***R.*** *We thank the reviewer for this very important comment. We intervened in a twofold manner. First, we made explicit that we organized the Result Section on the basis of the four research questions reported in the Materials and Method Section (see lines 275-279). Second, we briefly articulated our role in identifying themes/patterns then discussed in the Discussion section. These considerations were incorporated both in the* ***revised abstract*** *and in the revised main text (see* ***Discussion*** *section, lines 630-638).*

5. Additional comments

Line 632 – include page number on the reference after “to provide an analysis of vulnerability that does not render it vacuous, rescues its force and importance”

***R.*** *Done.*

6. Line 769 -770 - The first sentence should be referenced.

***R.*** *We thank the reviewer for this comment. However, after a careful assessment, we decided to delete this sentence and the related passage since no longer consistent with the reasoning flow of the revised manuscript.*

785-6 - Previous studies in older adults’ populations, consider “Previous studies in adult populations.”

***R.*** *Done.*

**Reviewer #4**

This manuscript clearly reports a systematic review that focuses on vulnerability in research ethics theory and practice. Specifically, the paper focuses on the identification and definition of vulnerable populations as contained in the selected policy documents. It is impressive that several sources of the ethics literature were employed in this review.

More so, the application of the PRISMA guidelines (PRISMA-Ethics guidelines) to review the selected policy documents for a non-empirical (quantitative or qualitative) ethics literature is commendable.

It is noteworthy that virtually all international guidelines and policies (in English language) that include vulnerability language were included in the review. So, actors in the drafting, implementing, and reviewing of human research protection policies regarding vulnerability across local and international research landscapes could consult the paper for reference or as a guide.
In this paper, the authors began their review by clearly stating the problem of disagreement in the meaning of vulnerability which several published literatures have equally echoed. It is important to underline the perennial challenge in arriving at a harmony in the identification, use, and application of vulnerability in research ethics. Although the paper focuses on research ethics which is clearly understandable, the restriction of the scope within the research ethics field. So, it did not cover clinical, environmental and other fields where the concept of vulnerability applies. Hopefully, readers interested in vulnerability as a general concept could expand the current understanding which is limited to research ethics. This rigorous work should be regarded as a good starting point for systematic reviews on the topic and so, could make a good resource for policy makers and those interested in vulnerability in research.

***R.*** *We thank the reviewer for his appreciation of our work, and we hope he will be satisfied with the revised text.*

Major Issues:
The purpose of a systematic review as opposed to other forms of review or studies is to provide a summary of the existing body of and quality of the available evidence on any topic and so, the quality or the certainty of that evidence becomes a priority in the review process. So, besides the detailed review process, the quality of the review comes first in the judgment of whether the manuscript is publishable or not. Therefore, the bulk of this review centred on how the paper complied with the stated recommended format for reporting systematic reviews (of ethics and non-ethics literature).
It is of note that the authors reviewed detailed application of the subject, pointing out the different definitions and descriptions of vulnerability. This is commendable, however, beginning with the abstract, the authors should pay attention to the suggestions and where necessary provide clear rationale for not including those suggestions. The purpose of highlighting these methodological issues is to ensure that what is published afterwards can be trusted evidence which why a systematic review is recommended in the first instance.

1. The authors did not include important information in the abstract. The abstract is an important section that gives an overview of the strength of evidence of the review. Although the body of the manuscripts contain some information that is not clearly stated in the abstract, it will be helpful if the authors review each section of the abstract to see if the abstract meets either the PRISMA guidelines or the PRISMA-Ethics guidelines. <https://osf.io/f3ys5/> (DOI 10.17605/OSF.IO/F3YS5).

***R.*** *The reviewer is right in pointing out that our original abstract was not enough informative. We completely* ***revised the abstract*** *according to PRISMA-Ethics guidelines (see the detailed reply below).*

2. Where necessary, the authors should provide line by line the reason(s) for each recommendation for reporting abstract of a systematic review of the ethics literature using PRISMA-Ethics or the original PRISMA guidelines.

The following are some examples:

i. The abstract did not clearly state the objective of the systematic review. The abstract only contains, in the appropriate section, the problem the authors considered in the review but not the objective of the review. The rationale should include the problem and why it is being addressed, including the objective of the review. This information should be clearly stated in the body of the manuscript.

***R.*** *We inserted the objective of the systematic review in the* ***revised abstract****. We also included a better description of the rationale of this review in the revised body of the manuscript (see* ***Introduction*** *section, lines 97-109).*

ii. It is challenging to judge the completeness of the literature search and how current the search was since important missing items such as inclusion and exclusion criteria, the rationale for selecting the databases, the source of the grey literature, the date of the search, the search itself (how and how many authors performed the search per stage). The authors are advised to check for the explanation and checklist of the PRISMA-Ethics/PRISMA guidelines to include missing information (in the abstract and the main section of the paper) about the search strategy.

***R.*** *We inserted the missing information in the abstract, as requested.*

iii. The authors should state if automation tools, Ai tools and other tools were used at any stage in the review and clearly state the tool(s), where and how they were used.

***R.*** *We have specified in the abstract that no automation tool or other tool was used at any stage of the review.*

iv. Although the limitations of the study were highlighted in the main section of the manuscript, this was missing in the abstract.

***R.*** *We have added a sentence mentioning the limitation of our study in the abstract, as rightly pointed out.*

v. There is no argument whether this review requires an assessment of risk of bias or grading of level of evidence; it is not likely that it requires any of these assessments but the lack of clarity as how the authors addressed sources of reporting/ selection bias and the lack of description of the steps used in each stage of the review weakens the strength of the evidence. The authors, therefore, should clearly state how they addressed different types of potential biases in this review.

***R.*** *To address and minimise potential sources of bias in the selection and analysis of documents, we set up several strategies.*

*First, the authors a priori defined inclusion and exclusion criteria, as reported in the “Materials and Method” Section.*

*Secondly, authors independently screened the included documents. Document selection was conducted by the two co-first authors (AG and GB), in consultation with the last author (VS).*

*Data extraction was performed using the standardised QUAGOL methodology, applied on the whole set of documents.*

*Given the specific type of the sources (policy documents and published guidelines), in our view, no formal risk of bias tool (e.g., AGREE II, AACODS) was applicable.*

*However, the credibility of the sources was assessed based on issuing authority, publication date, scope, and relevance to our research questions.*

*Limitations of this approach are reported in the “Strength and limitations” Section.*

vi. QUAGOL coding results are noted but the five steps employed by the authors are critical in judging the quality of the methods. To improve the quality of this review, the authors should include the description of the stages utilized for the QUAGOL method.

***R.*** *We thank the reviewer for this comment. We have added a brief description of the stages of the QUAGOL methodology followed in this review in the “Materials and Method” Section (see lines 221-228) as well as in the* ***revised abstract****.*

vii. It is not clear why grey literature was conducted only in Google Scholar while there are online sources of grey literature. It is possible that, besides using just a few databases in the search strategy, more grey literature could have been enlisted in the review where Google scholar not used as the only source of grey literature.

***R.*** *We consulted a grey literature database exclusively to conduct an additional verification, aimed at ensuring the inclusion of all relevant international documents either originally published in English or translated into English on the topic. While the three compiled lists, when considered jointly, should be comprehensive, we nonetheless opted to strengthen the rigor of our approach by supplementing them with a targeted search both in a bibliographic database and in grey literature, using Google Scholar, which is our standard tool for such tasks. Should the reviewer consider it necessary, we remain available to conduct an additional search in a different grey literature database, although we are confident that no further relevant documents would be identified.*

viii. In principle, systematic review protocols should be registered. Registration helps to maintain transparency and to avoid unnecessary study duplication by other authors. The authors might find it plausible to register the protocol used in this review. Again, increased transparency in the entire review process helps to judge the quality of evidence provided by this review.

**R.** The reviewer is right in highlighting the importance of registering systematic reviews in public databases, as this contributes to greater transparency. We attempted to register our systematic review in PROSPERO as suggested; however, during the submission process, we realized that it was no longer possible to proceed due to time constraints. As stated in point 5 of the PROSPERO guidelines: *"Please note: Reviews that have progressed beyond the point of completing data extraction at the time of initial registration are not eligible for inclusion in PROSPERO."* We noticed that this applies to other databases as well. We apologize for this and will take it into consideration for future systematic review projects.

Minor issues
1. When reviewing international policies, it is important that within the review period, there is a cross-checking of each document for updates because some inf might have changed, modified or deleted. In this review, one of the consolidated guidelines from the Office of Human Research Protections (OHRP) (see page 4, line 106-7, reference 6, page38, line.844-46), the International Compilation of Human Research Standards, has been updated. So, the 2021 edition cited in this review should be replaced with the 2024 edition. (<https://www.hhs.gov/ohrp/international/compilation-human-research-standards/index.html>).

***R.*** *The reviewer is right. As stated in the Materials and Methods section of the manuscript, at the time of submission we conducted a verification by repeating the search. In the case of the lists, we checked whether any document had been updated and, if so, identified which documents had changed. We found that five documents had been updated in the meantime—for example, the Declaration of Helsinki. The reviewer’s observation likely stems from our oversight in not updating the corresponding reference in the bibliography. We apologize for this mistake. Given that the paper underwent a second round of review, we took the opportunity to recheck all the sources once again to ensure that no outdated data remained.*

2. Although this is not the focus of the study, this review on vulnerability could have been more robust if other views or notions for or against the labelling of research participants as vulnerable or in what better ways the term or concept could be used such as vulnerability as a dynamic state, a situation applicable in context and having a spectrum where there are individuals or groups at one point of the spectrum at a given time and circumstance, the under and over-inclusiveness of the term, are included in the discussion section. More so, the notion of vulnerable people as stigmatizing and those providing the reason for additional protection (such as the research ethics committees) becoming paternalistic and consequently, unfairly excluding these labeled participants, should have been highlighted. It is important to note that not only is labelling important but the situation too. More so, reviewed guidelines focus on the protection from valid informed consent may not be sufficient in protecting vulnerable participants.

Reference: Rogers WA. Vulnerability. In: Laurie G, Dove E, Ganguli-Mitra A, et al., eds. The Cambridge Handbook of Health Research Regulation. Cambridge Law Handbooks. Cambridge University Press; 2021:17-26.

***R.*** *The reviewer is correct in noting that the discussion lacked sufficient elaboration on the limitations of the labelling approach. We thank the reviewer for the suggested reading, which has helped us better delineate the boundaries of this approach. We have incorporated and discussed these limitations in the revised* ***Discussion section*** *(see lines 647-648, 668-670, 707-719, 727-743).*
